# Mechanism of 3‐Methylglutaconyl CoA Decarboxylase AibA/AibB: Pericyclic Reaction versus Direct Decarboxylation

**DOI:** 10.1002/anie.202008919

**Published:** 2020-10-16

**Authors:** Xiang Sheng, Fahmi Himo

**Affiliations:** ^1^ Department of Organic Chemistry Arrhenius Laboratory Stockholm University SE-10691 Stockholm Sweden

**Keywords:** decarboxylation, enzymes, pericyclic reactions, quantum chemistry, reaction mechanisms

## Abstract

The enzyme 3‐methylglutaconyl coenzyme A (CoA) decarboxylase (called AibA/AibB) catalyzes the decarboxylation of 3‐methylglutaconyl CoA to generate 3,3‐dimethylacrylyl‐CoA, representing an important step in the biosynthesis of isovaleryl‐coenzyme A in *Myxococcus xanthus* when the regular pathway is blocked. A novel mechanism involving a pericyclic transition state has previously been proposed for this enzyme, making AibA/AibB unique among decarboxylases. Herein, density functional calculations are used to examine the energetic feasibility of this mechanism. It is shown that the intramolecular pericyclic reaction is associated with a very high energy barrier that is similar to the barrier of the same reaction in the absence of the enzyme. Instead, the calculations show that a direct decarboxylation mechanism has feasible energy barriers that are in line with the experimental observations.

## Graphical abstract

The reaction mechanism of AibA/AibB is studied by means of quantum chemical calculations. The intramolecular decarboxylation through a pericyclic transition state is ruled out on the basis of its high energy barrier. Instead, the direct decarboxylation involving an enolate intermediate is shown to have feasible barriers.

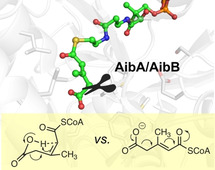

Isovaleryl‐coenzyme A (IV‐CoA) is an important metabolite in myxobacteria because it is a precursor for various compounds of life importance, such as iso‐fatty acids, which are the major acyl constituents of membrane lipids.^[1]^ IV‐CoA is generally derived from the degradation of leucine. However, very recently, an alternative biosynthesis pathway (Scheme 1) that requires acetyl‐CoA as the starting component was discovered in *Myxococcus xanthus*, providing a complementary strategy to ensure the production of IV‐CoA‐derived metabolites when the general route is inactive.[^2^, ^3^, ^4^, ^5^] The AibA/AibB enzyme was identified as a key component in this alternative pathway, catalyzing the decarboxylation of 3‐methylglutaconyl CoA (MG‐CoA) to generate 3,3‐dimethylacrylyl‐CoA (DMA‐CoA).

**Scheme 1:**
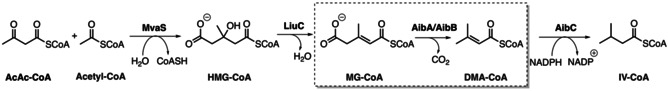
Alternative isovaleryl‐coenzyme A (IV‐CoA) biosynthesis pathway in *Myxococcus xanthus*.

The AibA/AibB‐catalyzed reaction was initially proposed to follow a two‐step direct decarboxylation mechanism involving an enolate intermediate (Scheme 2 a).^[4]^ Sequence comparison of AibA/AibB with its closest structural homologues, glutaconate CoA transferases (Gcts), revealed that a conserved catalytic glutamate residue in Gcts is replaced by a cysteine (Cys56_B_) in AibA/AibB. Mutation of this cysteine to glutamate or aspartate reduced the enzyme activity significantly, to 13 % and 1.2 % of the wild‐type for Cys56Glu and Cys56Asp mutants, respectively. Cys56_B_ was therefore suggested to be directly involved in the catalysis, acting as a general acid to protonate the enolate intermediate (Scheme 2 a).^[4]^ However, very low amounts of recombinant protein were obtained in that study, indicating that the low activities of the mutants might be due to misfolding of the protein.^[5]^

**Scheme 2:**
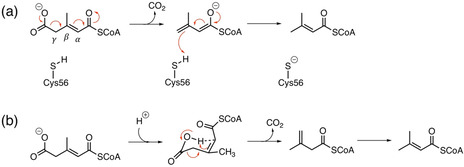
Proposed mechanisms for AibA/AibB: a) direct decarboxylation,^[4]^ b) intramolecular decarboxylation.^[5]^

A number of crystal structures of AibA/AibB were subsequently solved, and Cys56_B_ was seen to be the only possible general acid to participate in the reaction.^[5]^ However, the thiol group of Cys56_B_ was found to point away from the substrate, forming a hydrogen bond with Glu72_B_. A particularly interesting structure of AibA/AibB is that in complex with 3‐methylglutaconate (PDB: 5MZZ), which represents the acyl group of the MG‐CoA substrate. The ligand was found to adopt a bent conformation, and its γ‐carboxylate group, which corresponds to the carboxylate group of MG‐CoA, was located in an apparently hydrophobic cavity without forming any polar interactions with the enzyme.^[5]^

To further validate the role of Cys56_B_ in the catalysis, additional mutagenesis analysis was performed, and, interestingly, replacement of Cys56_B_ by Ala, Ser, Asn or Val did not eliminate the decarboxylation activity of the enzyme.^[5]^ The previously suggested direct decarboxylation mechanism, involving the cysteine residue, was thus doubted, and instead, an intramolecular decarboxylation mechanism was proposed that does not involve the participation of any residues from the enzyme (Scheme 2 b).^[5]^ In this mechanism, the acyl moiety of the substrate is assumed to adopt a bent conformation in the active site, and its carboxylate moiety is postulated to be in the protonated form due to the assumed hydrophobic nature of the binding pocket.^[5]^ The reaction proceeds through a six‐membered pericyclic transition state, which is a concerted step involving C−C bond cleavage and proton transfer from carboxy group to the α‐carbon. The resulting intermediate then isomerizes to yield the final product. This hypothesis, if correct, would make AibA/AibB unique among decarboxylases, as no other enzyme is known to exhibit such kind of reaction mechanism.^[6]^

In the present work, we examine the energetic feasibility of this novel pericyclic reaction mechanism by means of density functional theory (DFT) calculations.^[7]^ We compare it with the direct decarboxylation mechanism, and also with the uncatalyzed reaction in solution. We will show that the intramolecular pericyclic mechanism is associated with a prohibitively high barrier, and that the enzyme in that case does not provide much catalytic power compared to the solution reaction. The originally proposed direct decarboxylation mechanism, on the other hand, has quite feasible energy barriers that are consistent with the experimental observations.

The dispersion‐corrected B3LYP‐D3(BJ) hybrid functional[^8^, ^9^] was employed in the calculations, and a model of the active site consisting of 300 atoms (Figure 1) was constructed on the basis of the structure of AibA/AibB in complex with 4′‐diphospho pantetheine and acetate (PDB: 5MZX).^[5]^ Detailed information about the methods and the model is given in the Supporting Information (SI).

**Figure 1:**
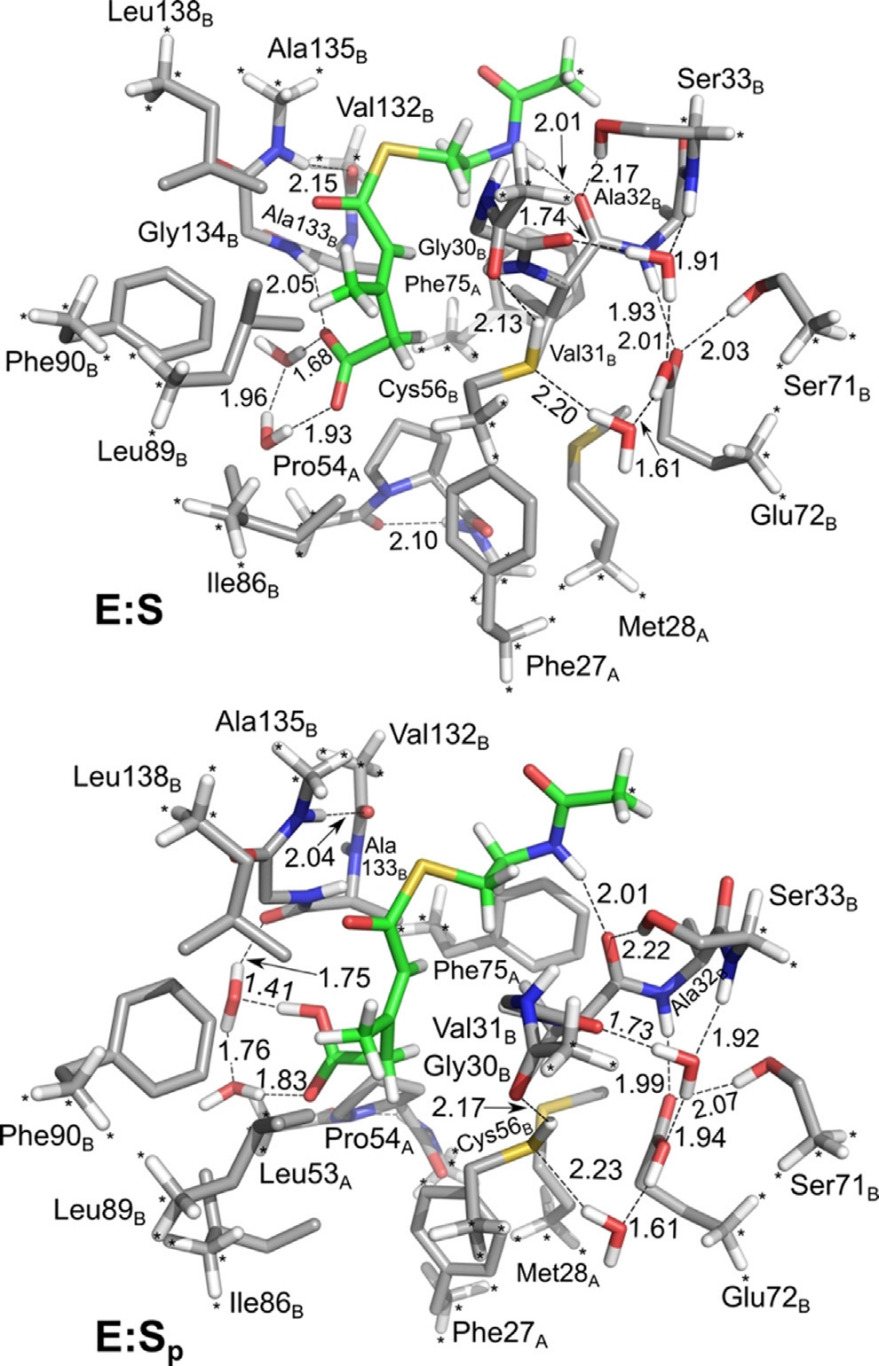
Optimized structures of the active site model. In **E:S** the carboxylate group of the substrate is in the deprotonated form, while in **E:S_p_** it is in the protonated form. For clarity, most of the hydrogen atoms are omitted. Selected distances are given in Ångstrom. Asterisks indicate fixed atoms in the geometry optimization.

While in the direct decarboxylation mechanism the carboxylate moiety of the substrate is in the deprotonated form, for the pericyclic reaction to take place this carboxylate has to be in the protonated form. Using the active site model, the p*K*_a_ of the enzyme‐bound substrate can be estimated to ca 8.6 (see SI for details), which indicates that it could be in either protonation state at the conditions of the experiment (pH 8.0).^[4]^ The enzyme‐substrate complexes for both scenarios, called **E:S** for the deprotonated case and **E:S_p_** for the protonated one, were therefore considered for the mechanistic investigations. The two structures were found to be very similar to each other (Figure 1), and also quite similar to the available crystal structures of the enzyme with bound ligands (see SI for superpositions).

It is important to point out that there is a void in the structure around the carboxylate part of the substrate. To fill the space, we have manually placed two water molecules there that are not visible in the crystal structure. We have also considered a model with only one water molecule at this location, and although some small energy differences could be observed between the two models, the conclusions are the same. The model with two water molecules will be discussed below, while the other one is given in the SI.

For the pericyclic reaction to occur from **E:S_p_**, the OH group of the carboxylic moiety must first adopt an *anti*‐configuration (**E:S_p_‐anti**) in order to deliver the proton to the α‐carbon. The energy of this intermediate (see SI) is calculated to be 11.0 kcal mol^−1^ higher than the *syn*‐configuration in **E:S_p_**. Outside the enzyme, this penalty is 6.0 kcal mol^−1^ (see SI), showing that the active site surrounding further disfavors the *anti*‐configuration, mainly because of steric clashes with Ala133 and Gly134 (see superposition of **E:S_p_‐anti** and **E:S_p_** in SI). From **E:S_p_‐anti**, the structure of the pericyclic transition state was optimized (**TS_pc_**, Figure 2) and was found to be as much as 34.2 kcal mol^−1^ higher than **E:S_p_**. Both the local TS structure, that is, the distances and angles of the breaking and forming bonds, and the energy barrier are quite similar to the pericyclic reaction outside the enzyme, which is calculated to have a barrier of 36.9 kcal mol^−1^ (Figure 2 and SI). These results clearly show that the pericyclic mechanism is not energetically viable. The fact that the enzyme surrounding does not provide much stabilization to the pericyclic transition state is in line with previous computational work on Diels–Alder reactions in enzymes.[^10^, ^11^]

**Figure 2:**
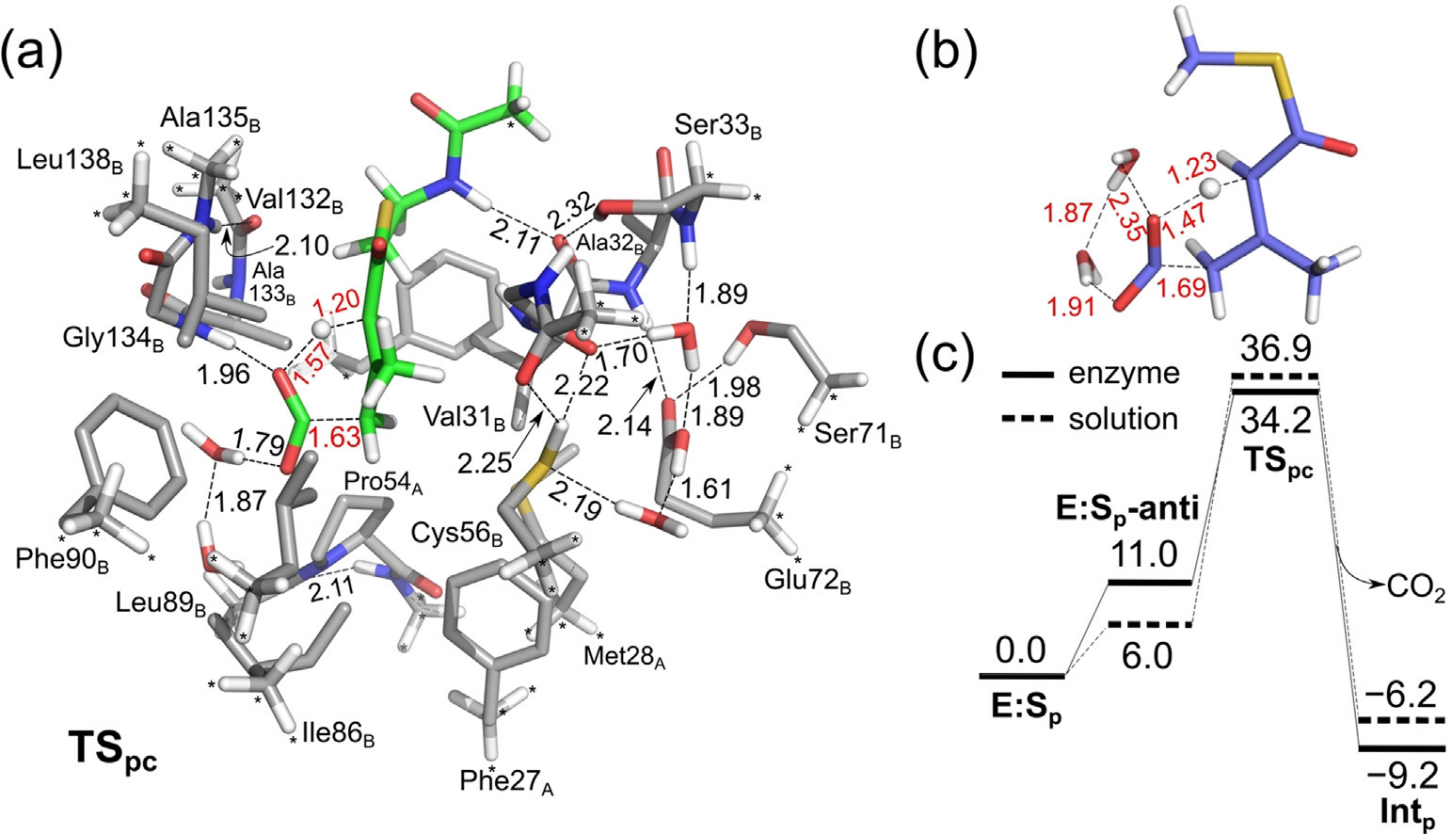
a) Optimized structure of the pericyclic transition state in AibA/AibB, b) transition state structure in solution, and c) calculated energy profiles.

We now turn our attention to the direct decarboxylation mechanism shown in Scheme 2 a. Starting from **E:S**, the C−C bond cleavage has a feasible barrier of 18.8 kcal mol^−1^, and the formed enolate intermediate **Int** is only 0.8 kcal mol^−1^ higher than **E:S** (Figure 3). Next, a proton transfer takes place from Cys56_B_ to the γ‐carbon of the enolate, to yield the 3,3‐dimethylacrylyl‐CoA product. The barrier is 13.1 kcal mol^−1^ and the formed enzyme‐product complex (**E:P**) is −2.0 kcal mol^−1^ relative to **E:S**. These results confirm thus that the previously suggested direct decarboxylation^[4]^ is energetically viable and that the cysteine indeed can act as a general acid in the reaction.

**Figure 3:**
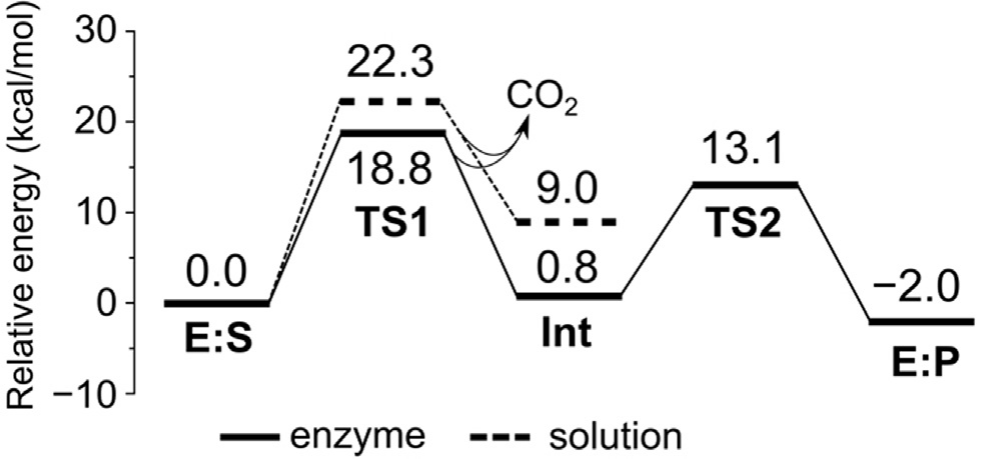
Calculated energy profiles for the direct decarboxylation mechanism in AibA/AibB and in solution.

In this mechanism, the generated CO_2_ molecule exits the active site directly after its formation. We have also examined energies of the case where CO_2_ remains in the active site throughout the reaction, but the calculations show that this scenario leads to a 10 kcal mol^−1^ higher barrier for the proton transfer (see SI). Another option considered was the C−C bond cleavage taking place concertedly with the proton transfer from the cysteine to the α‐carbon. However, this leads to a very high barrier of more than 40 kcal mol^−1^ (see SI).

For comparison, we have also calculated the energetics of the direct decarboxylation in water solution, that is, outside the enzyme (see details in SI). The calculations show that the barrier for the C−C bond cleavage is 22.3 kcal mol^−1^, that is, 3.5 kcal mol^−1^ higher than in the enzyme. The enolate intermediate is ca 8 kcal mol^−1^ higher than the corresponding intermediate in the enzyme case (Figure 3). From these results it is clear that the enzyme surrounding provides more stabilization to the enolate intermediate in **Int** compared to the substrate in **E:S**. However, it is not clear how this stabilization is achieved. Apart from a long hydrogen bond to the amide NH of Gly134_B_ (Figure 4), there is no oxyanion hole present at the active site like in other cofactor‐independent decarboxylases.[^6^, ^12^, ^13^, ^14^]

**Figure 4:**
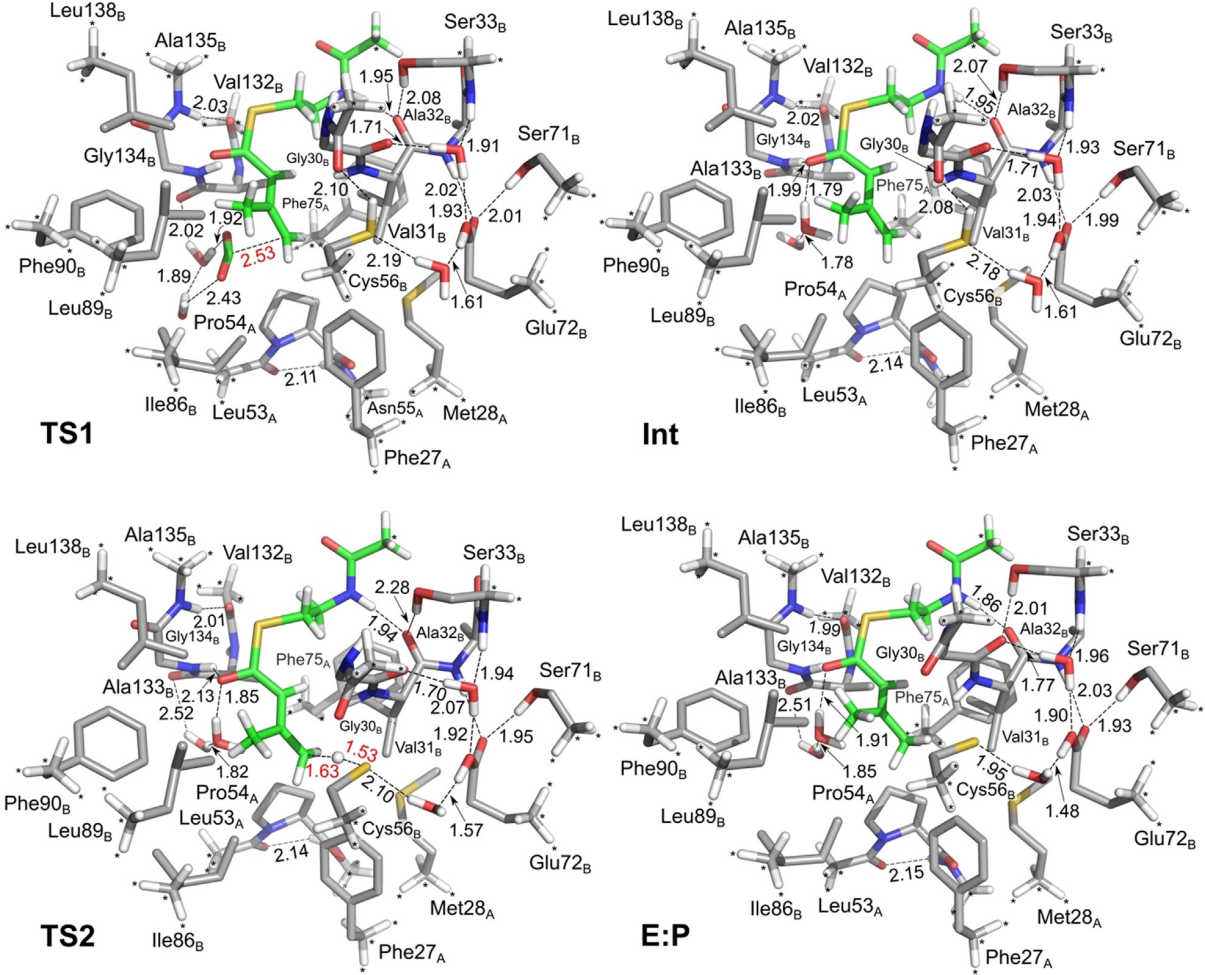
Optimized structures of intermediates and transition states in the direct decarboxylation mechanism of AibA/AibB.

It is important to note that the catalytic cycle is not complete at **E:P**. For that to happen, the product has to be released, the protonation state of Cys56_B_ has to be restored, and a new substrate has to bind. These steps are not possible to treat with the current approach and were not considered explicitly here. However, we found that at **E:P**, a proton transfer from the protonated Glu72_B_ to the deprotonated Cys56_B_ can take place barrierlessly via a bridging water molecule, with an exothermicity of 11 kcal mol^−1^ (see SI). This means that the glutamic acid would be the ultimate source of the proton that protonates the enolate intermediate.

The involvement of the Glu72_B_ as a proton source in the reaction could explain the experimental fact that mutation of Cys56_B_ to either of alanine, serine, asparagine or valine did not eliminate the activity.^[5]^ Namely, in the absence of the cysteine, Glu72_B_ could still deliver the proton to the enolate though water molecules. To examine this hypothesis, we re‐calculated the energies of the mechanism for the Cys56Ala variant, that is, where the cysteine in the model is replaced by an alanine. The barrier for the C−C bond cleavage was found to be 20.1 kcal mol^−1^, only 1.3 kcal mol^−1^ higher than the wild‐type, and the following proton transfer was found to have a barrier of only 7.7 kcal mol^−1^ (see SI for details).

Finally, since the protonation state of the Glu72_B_ residue is not known, we have also considered both the pericyclic and the direct mechanisms with an active site model in which the Glu72_B_ is in the ionized form. In both cases, the overall barriers are within 1–2 kcal mol^−1^ compared to the case with a protonated Glu72_B_ (see SI). However, the energies of the second step of the direct mechanism, that is, the protonation of the enolate intermediate by the cysteine, are affected more. The barrier is higher by 5.8 kcal mol^−1^ and the enolate is 14.1 kcal mol^−1^ higher compared to their counterparts in the mechanism with a protonated Glu72_B_. If the Glu72_B_ would indeed be in the ionized form, one can speculate that the protonation of the enolate intermediate could take place outside the active site, which would also be consistent with the mutational results.

To summarize the results of the present paper, the calculations unambiguously show that the intramolecular decarboxylation mechanism is not viable from energetic point of view. It is associated with a very high barrier, which the enzyme is not able to lower much compared to its solution counterpart. Instead, it is demonstrated that the enzyme follows a direct decarboxylation mechanism involving the formation of an enolate intermediate, as initially proposed.^[4]^ Moreover, the cysteine can be confirmed to act as a general acid, protonating the enolate intermediate that results from the direct C−C bond cleavage. In the absence of the cysteine, the protonation can be affected either by a nearby glutamic acid or in bulk solution.

## Conflict of interest

The authors declare no conflict of interest.

## Supporting information

As a service to our authors and readers, this journal provides supporting information supplied by the authors. Such materials are peer reviewed and may be re‐organized for online delivery, but are not copy‐edited or typeset. Technical support issues arising from supporting information (other than missing files) should be addressed to the authors.

Supplementary
