# Supplementary material for: Mechanism of 3‐Methylglutaconyl CoA Decarboxylase AibA/AibB: Pericyclic Reaction versus Direct Decarboxylation
Source: Angew Chem Int Ed Engl. 2020 Oct 16;59(51):22973–7. doi: 10.1002/anie.202008919 (PMC7756340; doi:10.1002/anie.202008919)
Supplement: Supplementary file 1 — Supplementary [file ANIE-59-22973-s001.pdf]

Angewandte Chemie
Eine Zeitschrift der Gesellschaft Deutscher Chemiker

Supporting Information

Mechanism of 3-Methylglutaconyl CoA Decarboxylase AibA/AibB: Pericyclic Reaction versus Direct Decarboxylation

Xiang Sheng and Fahmi Himo**

anie_202008919_sm_miscellaneous_information.pdf

Table of Contents

1. Computational details S2

2. Estimation of the pK_a of the enzyme-bound substrate S3

3. Superposition of E:S and E:S_p with crystal structures S4

4. Results of active site model with one water molecule S6

5. Additional results on intramolecular decarboxylation S8

6. Intramolecular decarboxylation mechanism in solution S9

7. Direct decarboxylation mechanism with CO_2 remaining in the active site S10

8. Concerted C-C bond cleavage and proton transfer S12

9. Direct decarboxylation mechanism in solution S13

10. Additional results on direct decarboxylation mechanism S14

11. Direct decarboxylation mechanism of the Cys56Ala mutant S15

12. Intramolecular decarboxylation mechanism with deprotonated $Glu72_B$ S17

13. Direct decarboxylation mechanism with deprotonated $Glu72_B$ S18

14. References S20

15. Absolute energies and energy corrections S21

16. Cartesian coordinates S22

1. Computational details

The calculations were performed with the B3LYP hybrid density functional method[1] implemented in Gaussian 09 program.[2] The Grimme's D3(BJ) dispersion correction was included in all the calculations.[3] The geometry optimizations were carried out with the 6-31G(d,p) basis set. At the same level of theory, single-point energies were calculated using the SMD solvation model with $\varepsilon = 4$.[4] Single-point calculations on the optimized structures were performed with the larger basis set 6-311+G(2d,2p) to get more accurate electronic energies. Frequency calculations were preformed to obtain zero-point energies (ZPEs). The entropy gain from releasing of CO_2 active site was estimated to be the translational entropy of the free molecule, which is 11.1 kcal/mol at room temperature. This value is added to the energy of the CO_2 formation step. Unless otherwise stated, the values presented throughout the paper are the large basis set energies (which include dispersion effect) corrected for solvation effects and ZPE.

The employed model of the active site was designed on the basis of the crystal structure of AibA/AibB in complex with 4'-diphospho pantetheine and acetate (PDB: 5MZX). The two ligands were manually replaced by the MG-CoA substrate. The acyl part of MG-CoA was orientated based on the position of 3-methylglutaconate identified in the active site of another crystal structure (PDB: 5MZZ). The constructed model consists of the truncated substrate, the important $Cys56_B$, and other residues that contribute to the active site pocket. These are: $Phe27_A$, $Met28_A$, $Leu53_A$, $Pro54_A$, $Asn55_A$, and $Phe75_A$ from chain A, and $Gly30_B$, $Ala32_B$, $Ser33_B$, $Cys56_B$, $Ser71_B$, $Glu72_B$, $Ile86_B$, $Leu89_B$, $Phe90_B$, $Val132_B$, $Gly134_B$, $Ala135_B$, and $Leu138_B$ from chain B. Two crystallographic water molecules that can form hydrogen bonds with $Cys56_B$ and $Glu72_B$ were also included in the model. In addition, two more water molecules were added to the model in the vicinity of the carboxylate group of the substrate, because a large void is observed there. The model consists of 298 or 299 atoms depending on the protonation state of the carboxylate group of the substrate. To avoid unrealistic movements during the geometry optimizations, a number of atoms were kept fixed. These are indicated by asterisks in the figures.

2. Estimation of the pK_a of the enzyme-bound substrate

The pK_a of the substrate bound to the enzyme can be estimated by comparing its deprotonation energy to that of acetic acid in aqueous solution, for which the pK_a has been measured to 4.76.[5]

$$\Delta E_1 = E(\mathbf{E{:}S_p})_{\varepsilon=4} - E(\mathbf{E{:}S})_{\varepsilon=4} = 285.9 \text{ kcal/mol}$$
$$\Delta E_2 = E(\mathbf{CH_3COOH})_{\varepsilon=78} - E(\mathbf{CH_3COO^-})_{\varepsilon=78} = 280.7 \text{ kcal/mol}$$
$$\Delta pK_a = [\Delta E_1 - \Delta E_2] / 2.303RT = 5.2 / 1.37 = +3.8$$

The pK_a of the enzyme-bound substrate is thus estimated to be ca 3.8 units higher than that of acetic acid, i.e. ca 8.6.

The result is the same when using an active site model with only one additional water molecule (ΔE_1 = 285.5 kcal/mol), or when using a mixed explicit-implicit solvent model for the acetic acid, with two explicit water molecules (ΔE_2 =280.7 kcal/mol).

3. Superposition of E:S and E:S$_p$ with crystal structures

The optimized structures of **E:S** and **E:S$_p$** show in general high similarities to the available crystal structures of the enzyme with ligands bound. Small movements can be detected for some of the active site residues, such as Phe75, Leu89, and Phe90. The carboxylate group of the substrate is rotated somewhat compared to the corresponding carboxylate group of 3-methylglutaconate in the crystal structure (Figure S1). Furthermore, some deviations are found for the CoA moiety in the optimized structure compared to the 4'-diphospho pantetheine in the crystal structure (Figure S2), due to the presence of 3-methylglutaconyl group in the substrate.

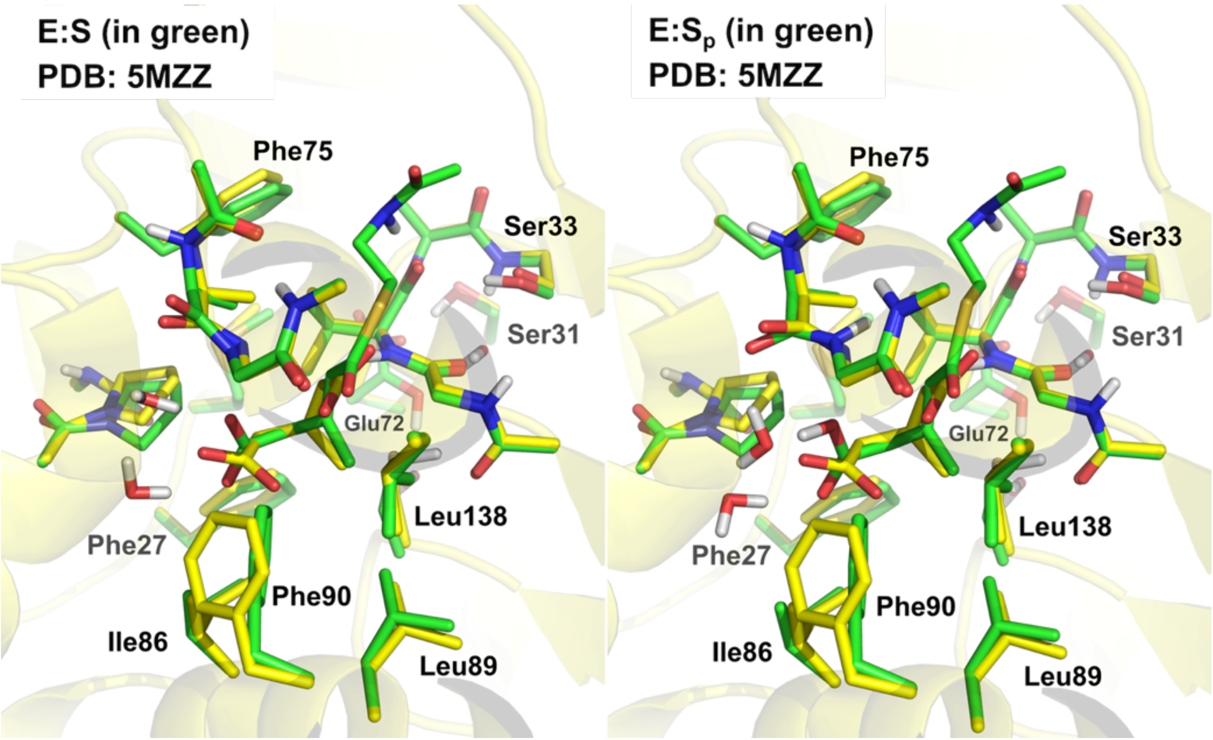

Figure S1*. Superpositions of the optimized structures of* ***E:S*** *and* ***E:S$_p$*** *with the X-ray structure in complex with 3-methylglutaconate (PDB: 5MZZ), which represents the 3-methylglutaconyl group of the substrate.*

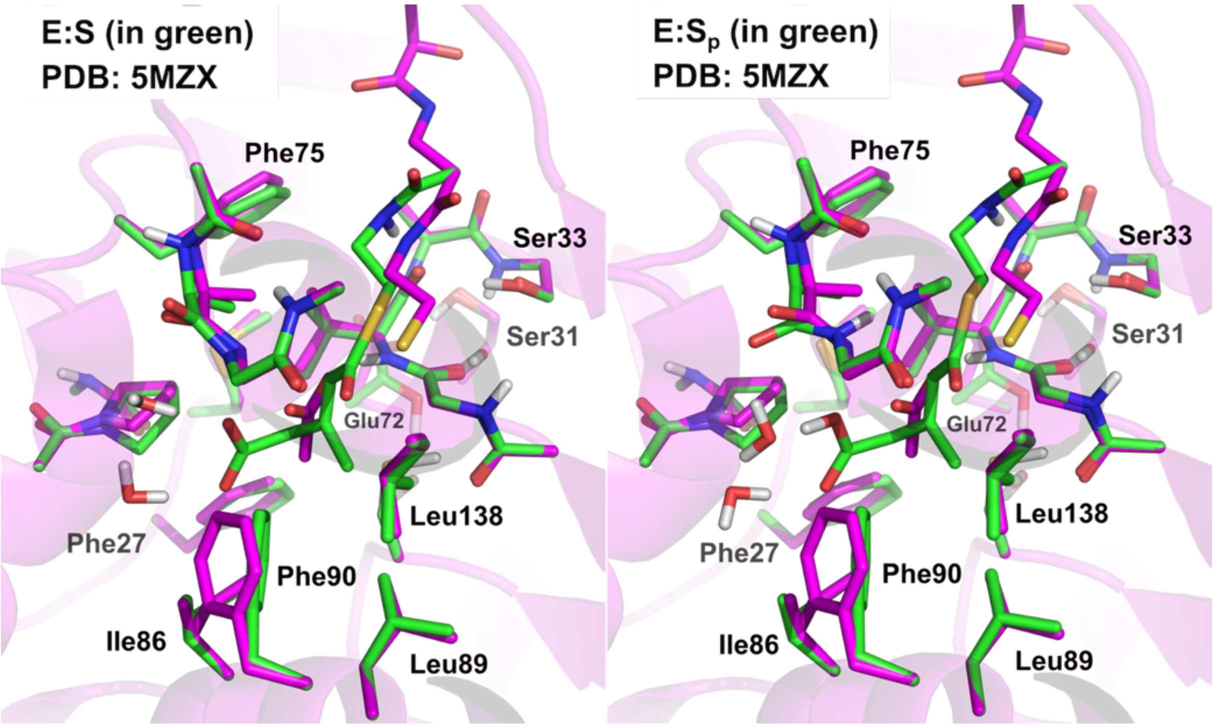

Figure S2. *Superpositions of the optimized structures of* ***E:S*** *and* ***E:S$_p$*** *with the X-ray structure in complex with acetate and 4'-diphospho pantetheine (PDB: 5MZX), which represents the CoA moiety of the substrate.*

4. Results of active site model with one water molecule

In addition to the active site model discussed in the main text, which contains two water molecules filling the void around the carboxylic moiety of the substrate, we have also considered a model with only one water molecule. The optimized structures of the active site model with the substrate in the deprotonated and protonated forms are shown in Figure S3. Comparison of the calculated energies of this model with those from the model with two water molecules are given in Figure S4 and Figure S5.

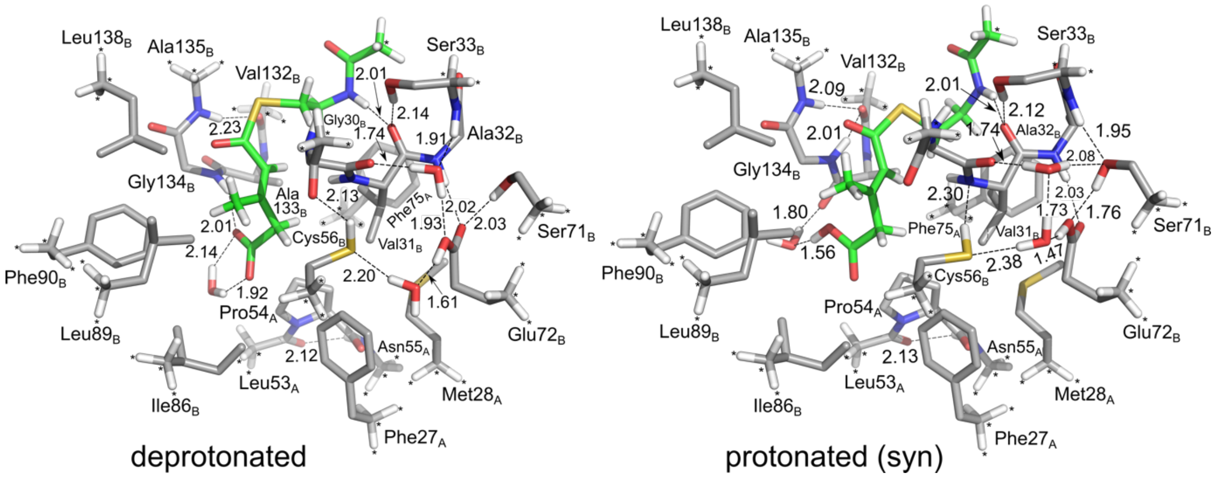

Figure S3. *Optimized structures of the active site model with one additional water molecule forming hydrogen bonds with the carboxylate group of the substrate. The protonation state of the carboxylate group of the substrate is indicated.*

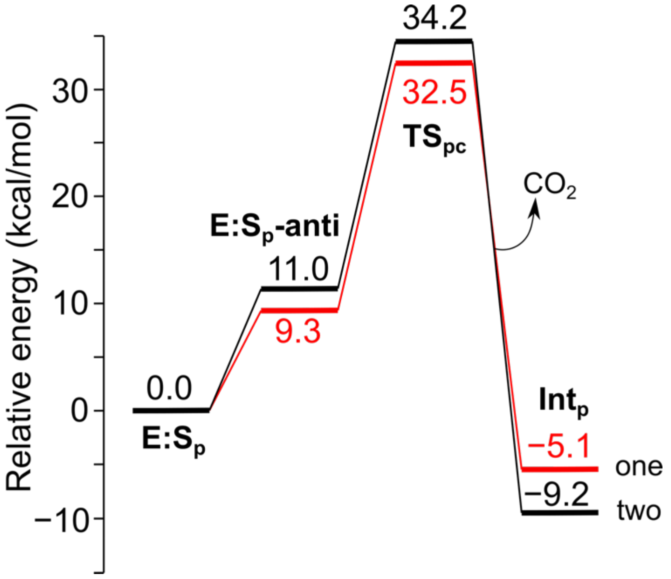

Figure S4. *Calculated energy profiles for the intramolecular decarboxylation mechanism calculated using active site models with one (red) or two (black) additional water molecules.*

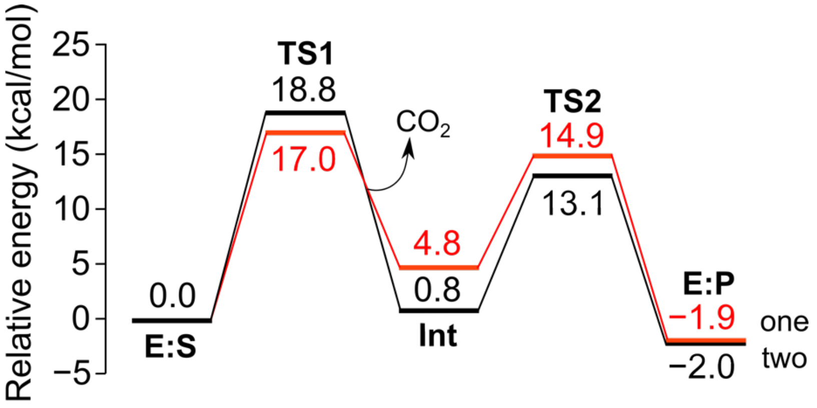

Figure S5. *Calculated energy profiles for the direct decarboxylation mechanism using active site models with one (red) or two (black) additional water molecules.*

5. Additional results on intramolecular decarboxylation

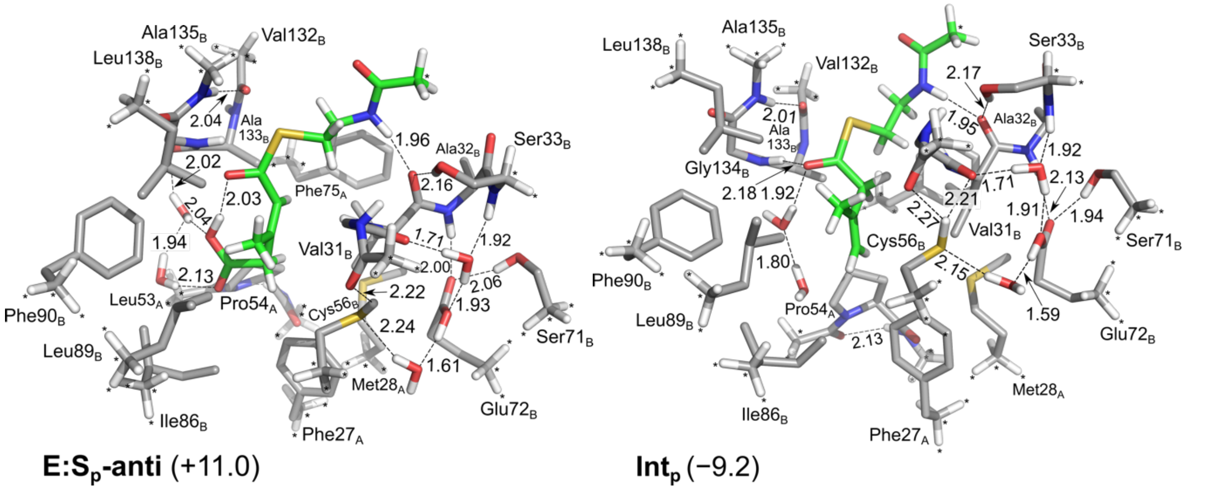

Figure S6. *Optimized structures of the enzyme-substrate complex with the carboxylic group of the substrate in an anti-conformation (**E:S$_p$-anti**), and the resulting intermediate of the pericyclic TS (**Int$_p$**). Energies relative to **E:S$_p$** with a syn-carboxylic group are given.*

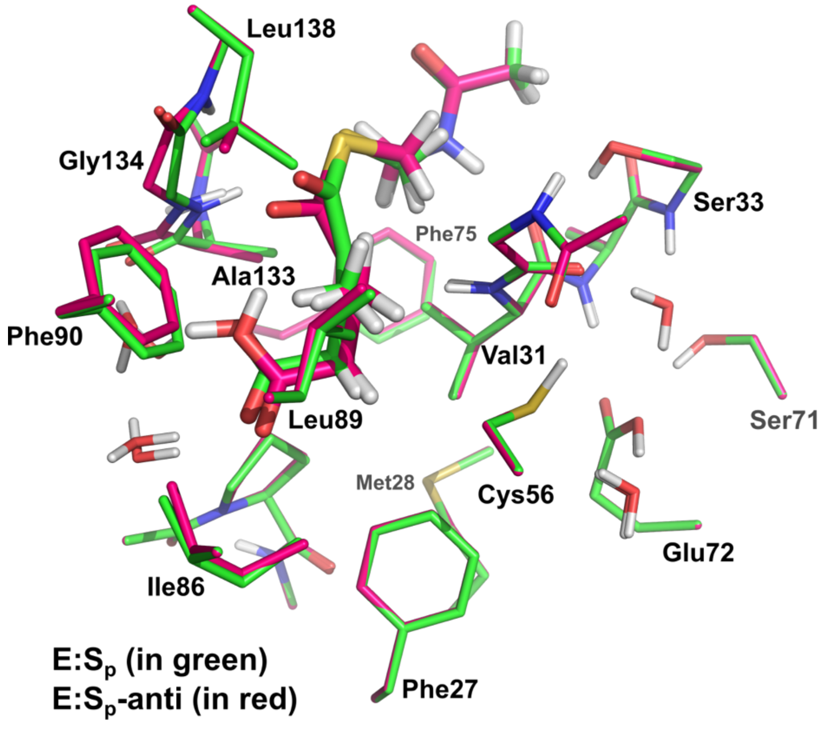

Figure S7. *Superposition of the optimized structures of **E:S$_p$** (green) and **E:S$_p$-anti** (red).*

6. Intramolecular decarboxylation mechanism in solution

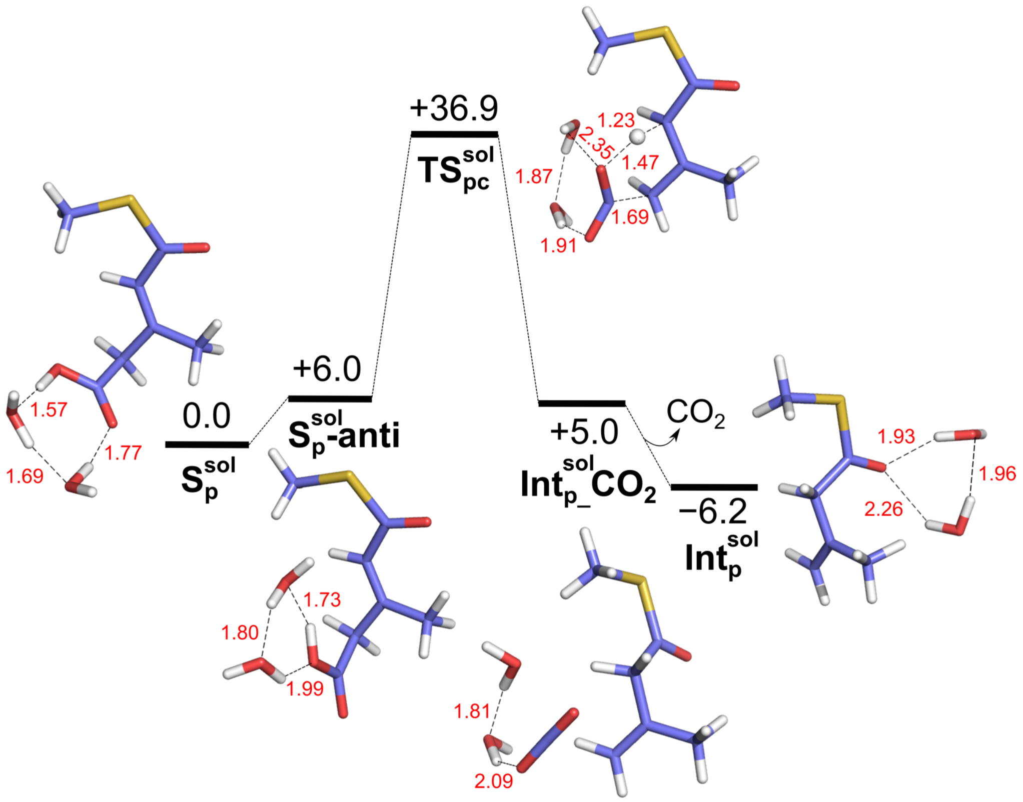

Figure S8. *Calculated Gibbs free energy profile (kcal/mol) and optimized structures for the intramolecular decarboxylation mechanism in solution. A mixed explicit-implicit solvation model was used, in which two explicit water molecules form hydrogen bonds to the carboxylate part of the substrate, similarly to the enzyme case.*

7. Direct decarboxylation mechanism with CO_2 remaining in the active site

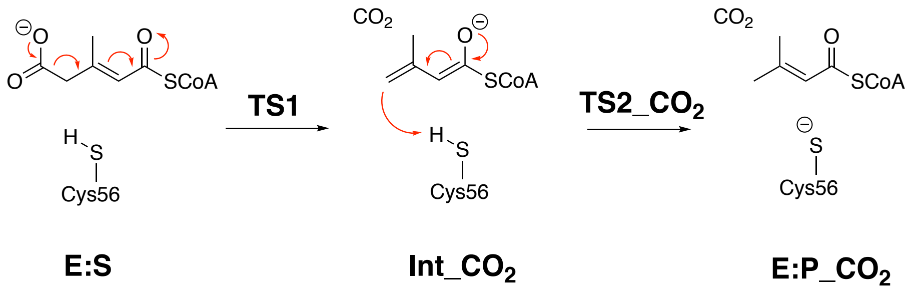

Scheme S1. *Alternative direct decarboxylation mechanism with CO_2 remaining in the active site throughout the reaction.*

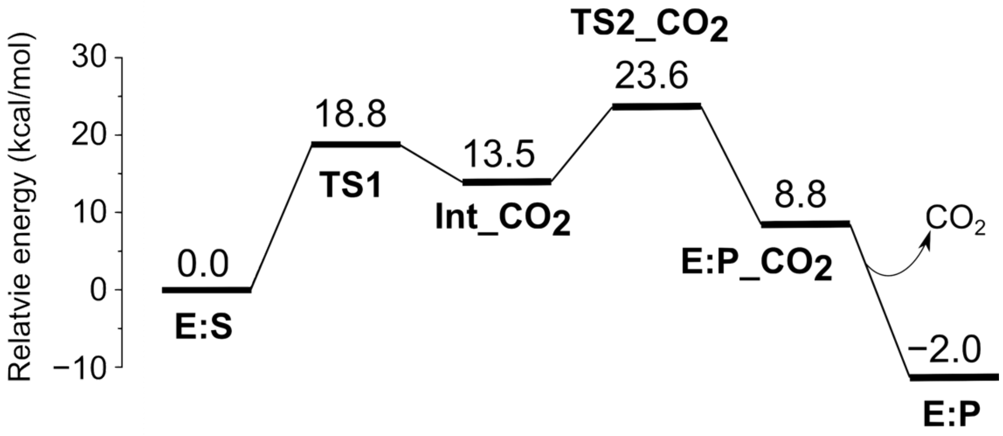

Figure S9. *Calculated energy profile for the alternative direct decarboxylation mechanism with CO_2 remaining in the active site throughout the reaction.*

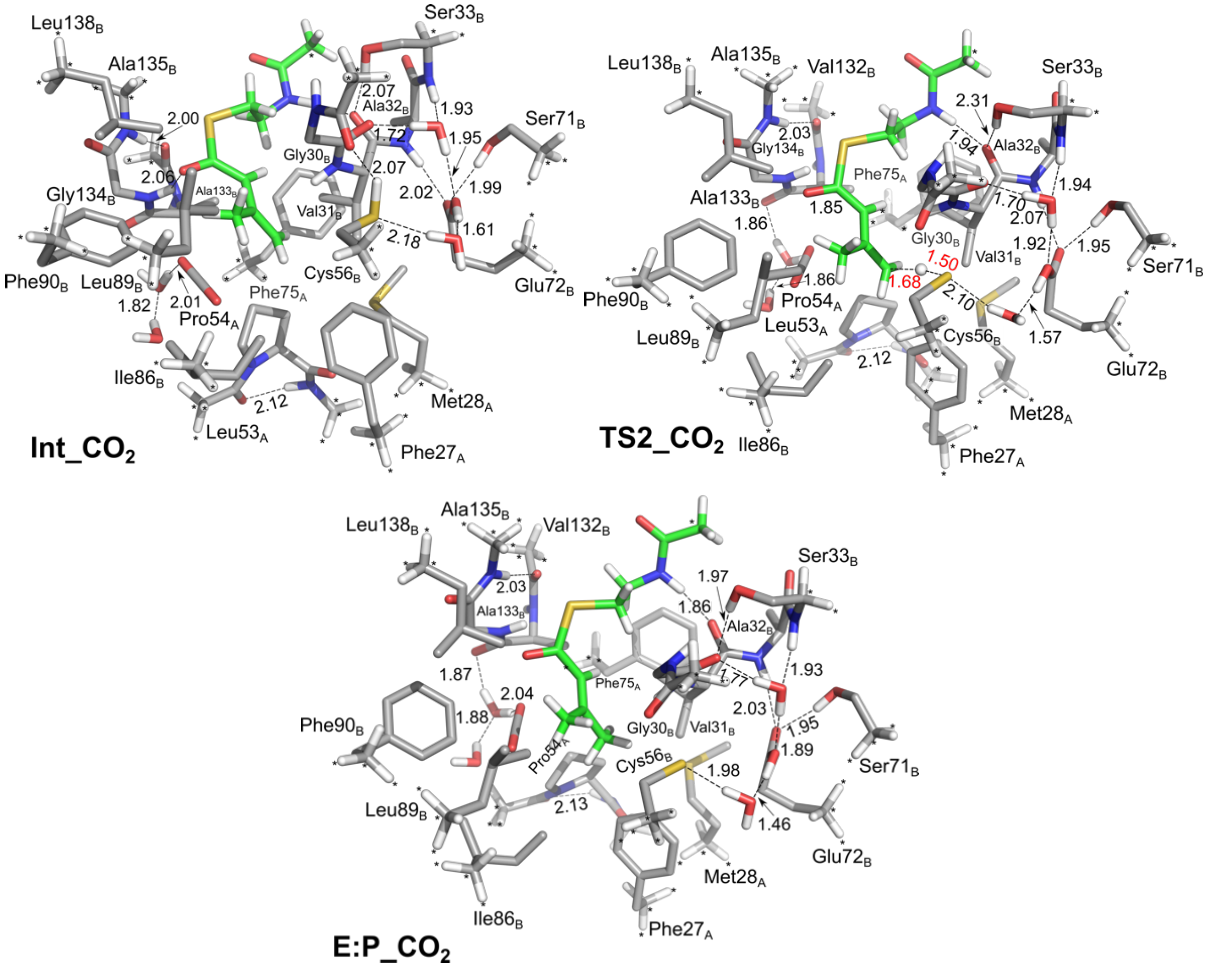

Figure S10. *Optimized structures for the alternative direct decarboxylation mechanism with CO_2 remaining in the active site throughout the reaction.*

8. Concerted C-C bond cleavage and proton transfer

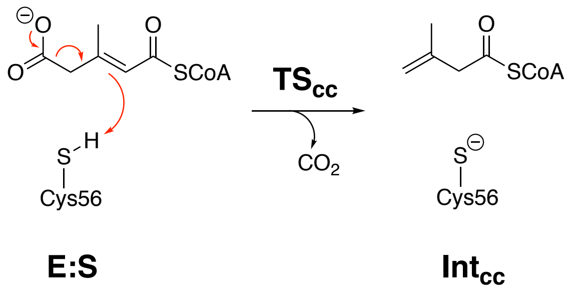

Scheme S2. *Alternative mechanism with the C-C bond cleavage taking place concertedly with the proton transfer from the cysteine to the α-carbon.*

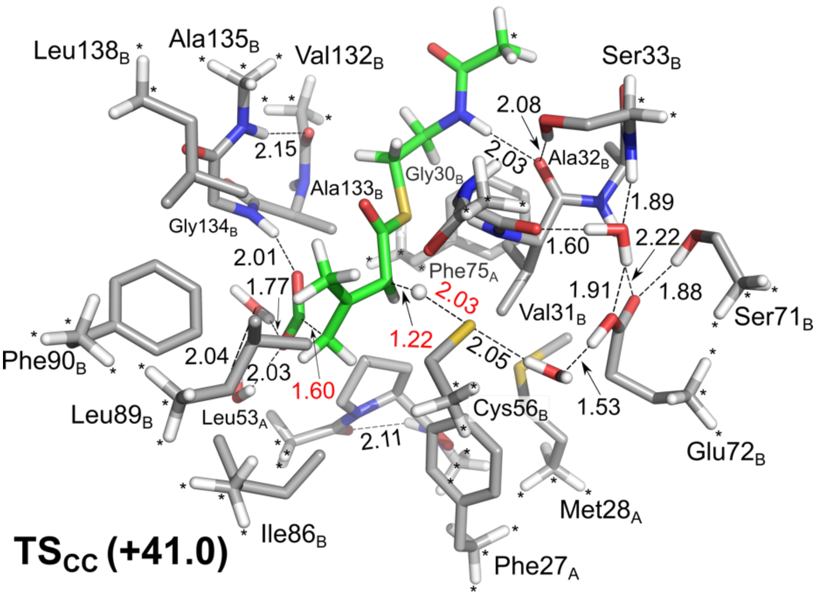

Figure S11. *Optimized transition state for the alternative mechanism with the C-C bond cleavage taking place concertedly with the proton transfer from the cysteine to the α-carbon. Calculated barrier is given in kcal/mol.*

9. Direct decarboxylation mechanism in solution

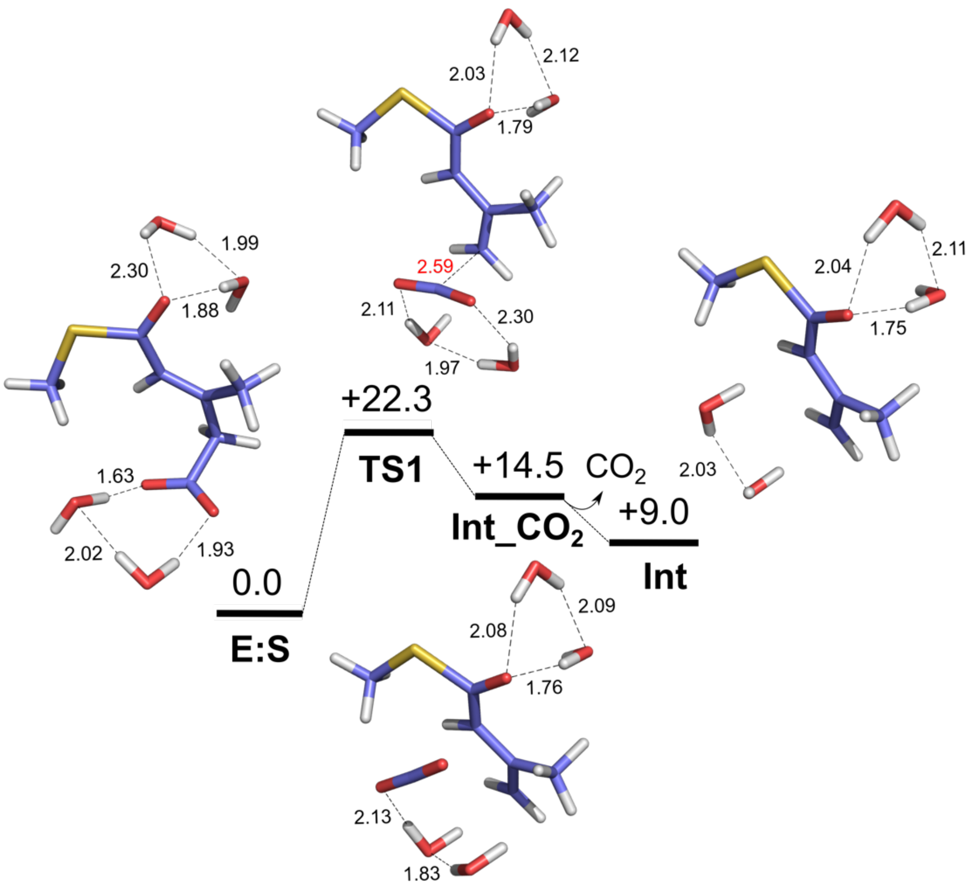

Figure S12. *Calculated Gibbs free energy profile (kcal/mol) and optimized structures for the direct decarboxylation mechanism in solution. A mixed explicit-implicit solvation model was used, in which two explicit water molecules form hydrogen bonds to the carboxylate part of the substrate, similarly to the enzyme case, and two additional water molecules form hydrogen bonds to the carbonyl oxygen.*

10. Additional results on direct decarboxylation mechanism

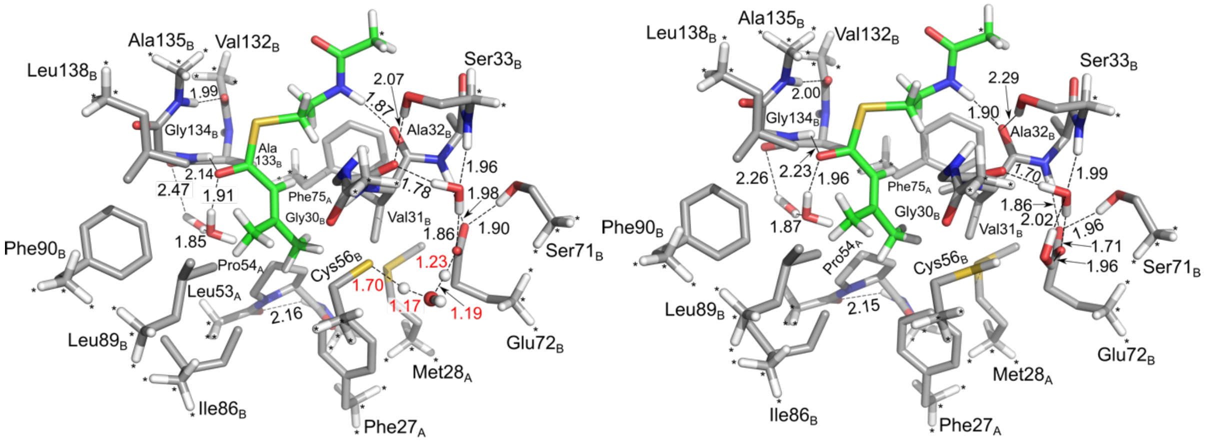

TS (Cys56$_B$-SH) (−3.4) **E:P (Cys56$_B$-SH)** (−13.0)

Figure S13. *Optimized structures of transition state* ***TS (Cys56$_B$-SH)*** *and the resulting enzyme-product complex* ***E:P (Cys56$_B$-SH)*** *for the proton transfer from Glu72$_B$ to Cys56$_B$.*

Note that the structure of **TS (Cys56$_B$-SH)** could be optimized at the level used for the geometry optimization. However, when all corrections were added (i.e. large basis set, solvation, and zero-point energy) the energy of the transition state becomes lower than **Int2**. The step can therefore be considered to take place without a barrier.

11. Direct decarboxylation mechanism of the Cys56Ala mutant

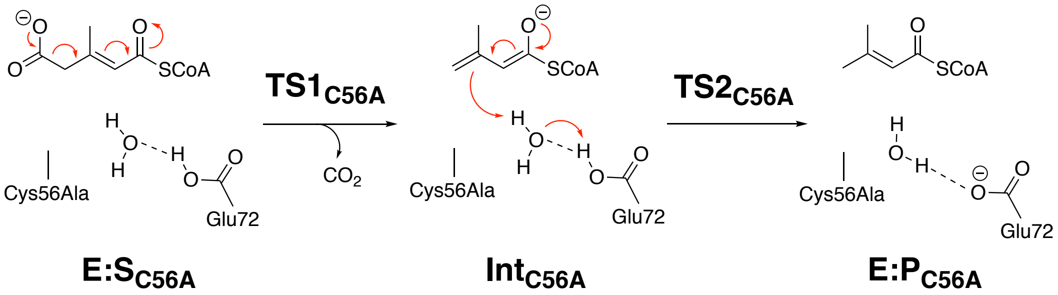

Scheme S3. *Direct decarboxylation mechanism for the Cys56Ala mutant.*

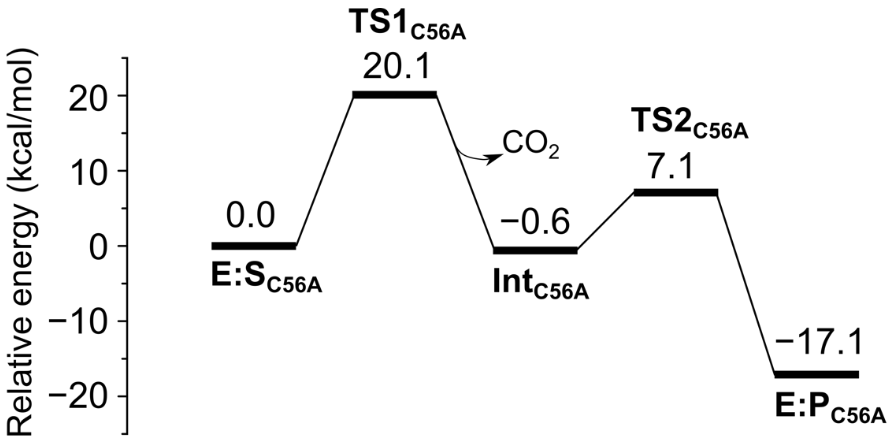

Figure S14. *Calculated energy profile for the direct decarboxylation mechanism of the Cys56Ala mutant.*

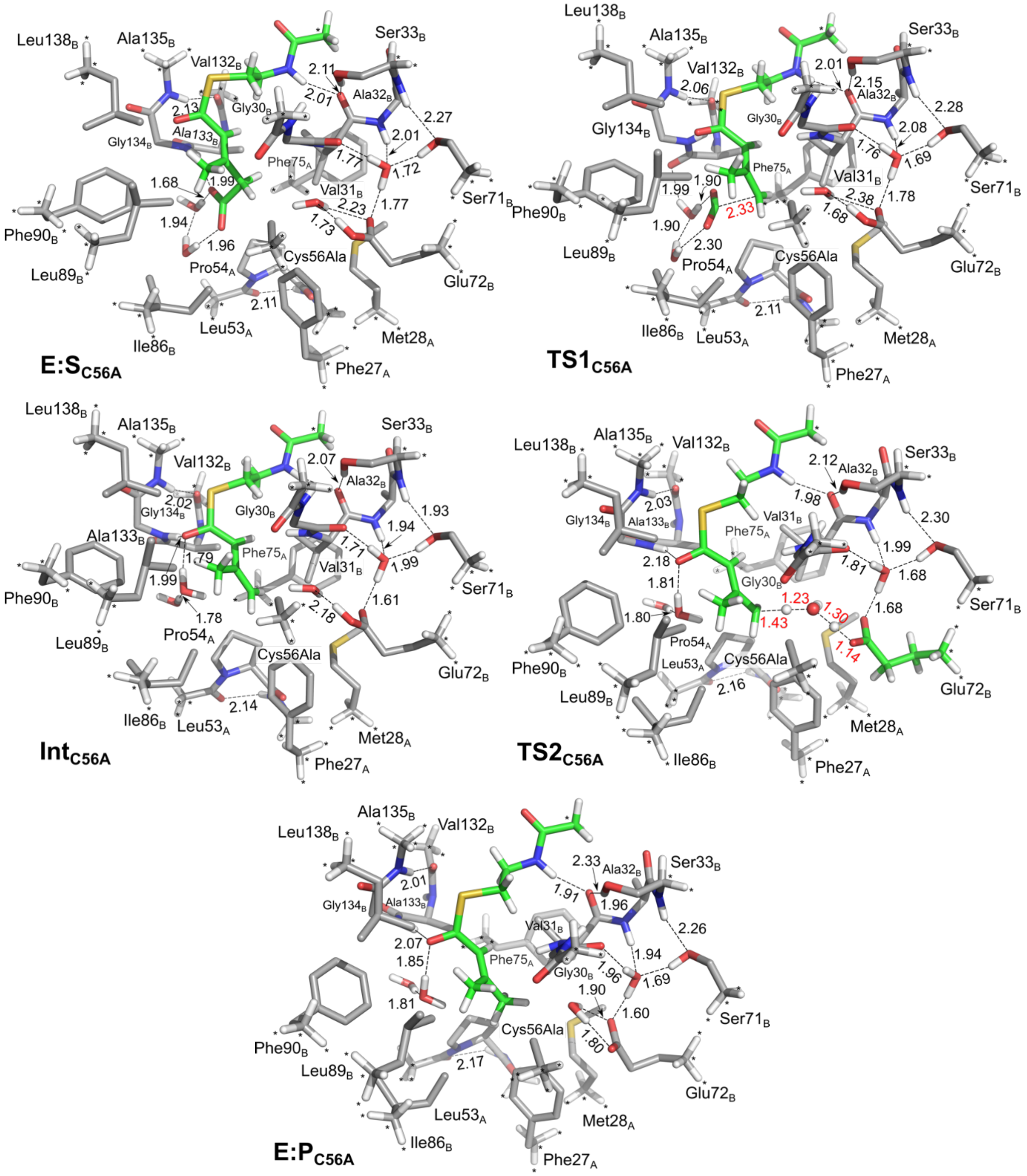

Figure S15. *Optimized structures of the intermediates and transition states for the direct decarboxylation mechanism of the Cys56Ala mutant.*

12. Intramolecular decarboxylation mechanism with deprotonated Glu72$_B$

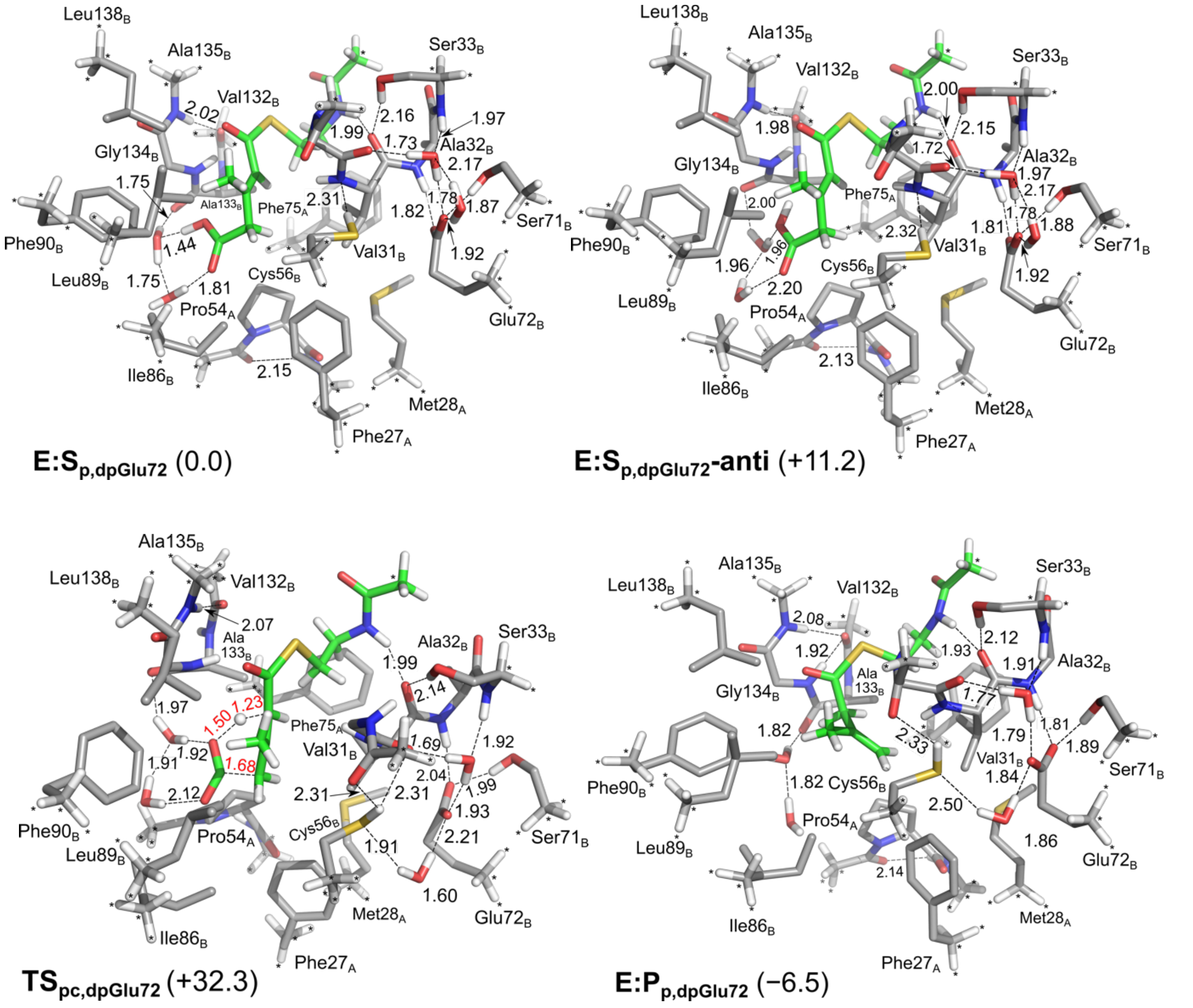

Figure S16. *Optimized structures of the intermediates and transition states for the intramolecular decarboxylation mechanism with Glu72$_B$ in the deprotonated form. Relative energies are given in kcal/mol.*

13. Direct decarboxylation mechanism with deprotonated $Glu72_B$

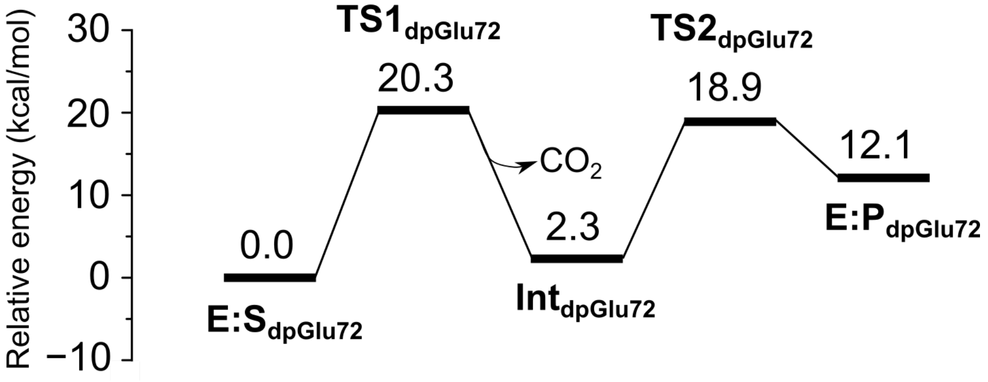

Figure S17. *Calculated energy profile for the direct decarboxylation mechanism with* $Glu72_B$ *in the deprotonated form.*

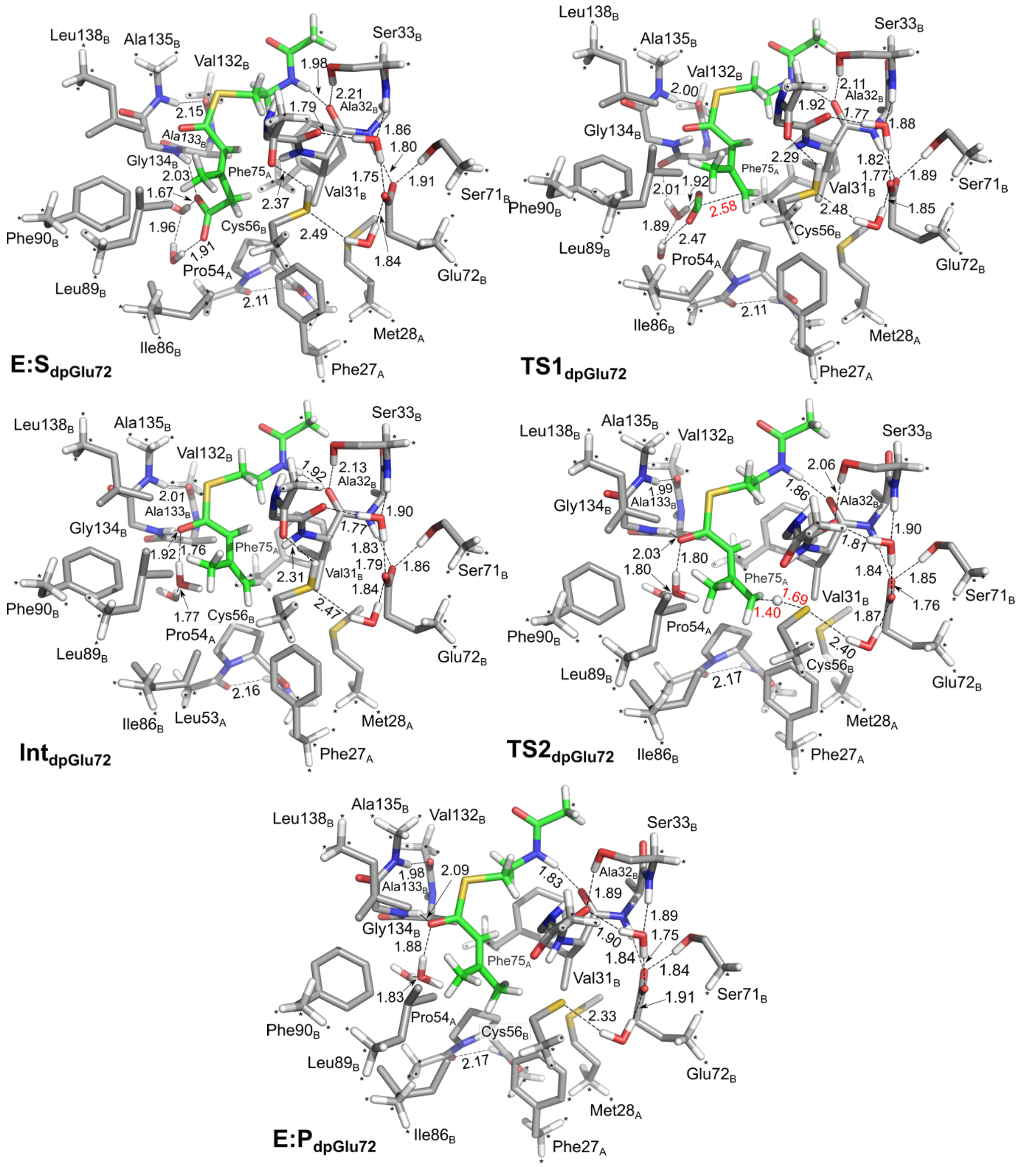

Figure S18. *Optimized structures of the intermediates and transition states for the direct decarboxylation mechanism with Glu72$_B$ in the deprotonated form.*

14. References

[**1**] (a) A. D. Becke, Density functional Thermochemistry. III. The Role of Exact Exchange. *J. Chem. Phys.* **1993**, *98*, 5648–5652; (b) C. Lee, W. Yang, R. G. Parr, Development of the Colle-Salvetti Correlation-energy Formula into a Functional of the Electron Density. *Phys. Rev. B* **1988**, *37*, 785–789.

[**2**] M. J. Frisch, G. W. Trucks, H. B. Schlegel, G. E. Scuseria, M. A. Robb, J. R. Cheeseman, G. Scalmani, V. Barone, G. A. Petersson, H. Nakatsuji, X. Li, M. Caricato, A. Marenich, J. Bloino, B. G. Janesko, R. Gomperts, B. Mennucci, H. P. Hratchian, J. V. Ortiz, A. F. Izmaylov, J. L. Sonnenberg, D. Williams-Young, F. Ding, F. Lipparini, F. Egidi, J. Goings, B. Peng, A. Petrone, T. Henderson, D. Ranasinghe, V. G. Zakrzewski, J. Gao, N. Rega, G. Zheng, W. Liang, M. Hada, M. Ehara, K. Toyota, R. Fukuda, J. Hasegawa, M. Ishida, T. Nakajima, Y. Honda, O. Kitao, H. Nakai, T. Vreven, K. Throssell, J. A. Montgomery, Jr., J. E. Peralta, F. Ogliaro, M. Bearpark, J. J. Heyd, E. Brothers, K. N. Kudin, V. N. Staroverov, T. Keith, R. Kobayashi, J. Normand, K. Raghavachari, A. Rendell, J. C. Burant, S. S. Iyengar, J. Tomasi, M. Cossi, J. M. Millam, M. Klene, C. Adamo, R. Cammi, J. W. Ochterski, R. L. Martin, K. Morokuma, O. Farkas, J. B. Foresman, D. J. Fox, Gaussian 09, Revision D.01; Gaussian, Inc.: Wallingford, CT, **2013**.

[**3**] (a) S. Grimme, J. Antony, S. Ehrlich, H. Krieg, A Consistent and Accurate Ab Initio Parametrization of Density Functional Dispersion Correction (DFT-D) for the 94 Elements H–Pu. *J. Chem. Phys.* **2010**, *132*, 154104; (b) S. Grimme, S. Ehrlich, L. Goerigk, Effect of the Damping Function in Dispersion Corrected Density Functional Theory. *J. Comput. Chem.* **2011**, *32*, 1456–1465.

[**4**] A. V. Marenich, C. J. Cramer, D. G. Truhlar, Universal Solvation Model Based on Solute Electron Density and on A Continuum Model of the Solvent Defined by the Bulk Dielectric Constant and Atomic Surface Tensions. *J. Phys. Chem. B* **2009**, *113*, 6378–6396.

[**5**] W. M. Haynes ed. (2016). CRC Handbook of Chemistry and Physics (97th ed.). CRC Press. pp. 5–88. ISBN 9781498754293.

15. Absolute energies and energy corrections

Table S1. *Calculated absolute energies and energy corrections. The entropy contribution of the release of CO_2 is included in **ΔE_{total}** for the corresponding intermediates and transition states. BS1=6-31G(d,p), BS2=6-311+G(2d,2p)*

	E_{BS1} (au)	E_{BS2} (au)	$E_{solvation}$ (au)	E_{ZPE} (au)	E_{total} (au)	ΔE_{total} (kcal/mol)
**Intramolecular decarboxylation mechanism:**						
	**Protonated carboxylate group of MG-CoA; Protonated Glu72$_B$**					
**E:S$_p$**	-6895.519666	-6897.246431	-6895.614377	2.615694	-6894.725449	**0.0**
**E:S$_p$-anti**	-6895.496823	-6897.225749	-6895.595215	2.616147	-6894.707994	**+11.0**
**TS$_{pc}$**	-6895.456715	-6895.551694	-6897.185075	2.609153	-6894.670902	**+34.2**
**Int$_p$**	-6706.911125	-6708.576203	-6707.017387	2.600457	-6706.082009	**-9.2**
	**Protonated carboxylate group of MG-CoA; Deprotonated Glu72$_B$**					
**E:S$_{p,dpGlu72}$**	-6895.023313	-6896.757884	-6895.139051	2.605475	-6894.268148	**0.0**
**E:S$_{p,dpGlu72}$-anti**	-6895.002167	-6896.739755	-6895.11838	2.605669	-6894.250299	**+11.2**
**TS $_{pc,dpGlu72}$**	-6894.962979	-6896.700708	-6895.079591	2.600572	-6894.216749	**+32.3**
**Int$_{p,dpGlu72}$**	-6706.415536	-6708.093713	-6706.531719	2.589505	-6705.620391	**-6.5**

	E_{BS1} (au)	E_{BS2} (au)	$E_{solvation}$ (au)	E_{ZPE} (au)	E_{total} (au)	ΔE_{total} (kcal/mol)
**Direct decarboxylation mechanism:**						
	**Deprotonated carboxylate group of MG-CoA; Protonated Glu72$_B$**					
**E:S**	-6895.016093	-6896.756523	-6895.132752	2.603362	-6894.269820	**0.0**
**TS1**	-6894.984342	-6896.728807	-6895.093281	2.597863	-6894.239882	**+18.8**
**Int**	-6706.404173	-6708.083729	-6706.517213	2.58625	-6705.610519	**+0.8**
**TS2**	-6706.391861	-6708.067812	-6706.499463	2.584505	-6705.590908	**+13.1**
**E:P**	-6706.417964	-6708.093863	-6706.52724	2.588188	-6705.614952	**-2.0**
**TS (Cys56$_B$-SH)**	-6706.414915	-6708.089966	-6706.524472	2.582293	-6705.617229	**-3.4**
**E:P (Cys56$_B$-SH)**	-6706.434674	-6708.110132	-6706.544939	2.587971	-6705.632426	**-13.0**
	**Deprotonated carboxylate group of MG-CoA; Deprotonated Glu72$_B$**					
**E:S$_{dpGlu72}$**	-6894.471021	-6896.220822	-6894.649771	2.591668	-6893.807905	**0.0**
**TS1$_{dpGlu72}$**	-6894.433471	-6896.189994	-6894.606058	2.586957	-6893.775624	**+20.3**
**Int$_{dpGlu72}$**	-6705.853654	-6707.544658	-6706.030508	2.575391	-6705.146121	**+2.3**
**TS2$_{dpGlu72}$**	-6705.825921	-6707.514217	-6706.003434	2.572024	-6705.119705	**+18.9**
**E:P$_{dpGlu72}$**	-6705.834216	-6707.523776	-6706.018009	2.577054	-6705.130515	**+12.1**
**CO_2**		-188.6519767		0.011593	-188.6403837	

16. Cartesian coordinates

Intramolecular decarboxylation mechanism:
Protonated carboxylate group of MG-CoA
Protonated Glu72$_B$

E:S$_p$ (0.0)

C	3.07408900	8.23657800	-1.59821600
C	2.25555900	7.57192200	-2.71974300
C	2.06012000	6.07937500	-2.55255900
C	1.06774400	5.57334400	-1.70202800
C	2.84766000	5.16558600	-3.26637500
C	0.84958300	4.20075600	-1.58715100
C	2.64152800	3.78991400	-3.14728900
C	1.63738000	3.30387200	-2.31005200
H	2.56392400	8.16037200	-0.63381400
H	1.27232500	8.05353700	-2.78138900
H	2.74899400	7.75588200	-3.68112200
H	0.45310000	6.26400400	-1.13268300
H	3.61910800	5.54168300	-3.93378100
H	0.04608900	3.83518500	-0.95707400
H	3.24935700	3.09428700	-3.71996900
H	1.47889800	2.23624500	-2.21450400
C	5.85440500	6.20737900	0.05896500
C	5.72917800	4.83436400	-0.60755900
C	4.54359700	4.02847800	-0.06813200
S	4.73949300	3.40417700	1.64688200
C	6.03791000	2.14392000	1.39954900
H	5.88542000	6.08395000	1.14648800
H	5.57358700	4.96114700	-1.68826400
H	6.66107700	4.26503800	-0.49629500
H	3.65261200	4.65700800	-0.02064900
H	4.31094200	3.17675300	-0.71088000
H	6.16206600	1.62510400	2.35235700
H	5.75512800	1.40852700	0.64155900
H	6.99442100	2.59437300	1.12322400
C	-2.13315100	7.17182200	3.19164400
C	-0.69601600	6.72136300	3.39831400
O	-0.00195200	7.23825900	4.28409800
H	-2.79169000	6.50588800	3.76140800
N	-0.20798700	5.70669300	2.63429800
C	1.10235700	5.11361600	2.96784400
C	2.24886700	6.04132300	2.53253600
O	2.65299400	6.08854900	1.36934000
C	1.11517300	3.77718700	2.21537700
C	-0.36505700	3.47072200	1.97747700
C	-0.96091600	4.85492600	1.70744200
H	1.14810700	4.97362000	4.05357900
H	1.62586700	3.92592700	1.26101300
H	1.65186600	3.00336600	2.76790900
H	-0.51254500	2.79622300	1.13643900
H	-0.84625900	3.03349100	2.85812800
H	-0.77168000	5.16318700	0.67182500
H	-2.03261100	4.91312500	1.88412900
N	2.75953200	6.77122600	3.55030600
C	3.70406900	7.85267400	3.30541900
H	2.09235300	6.91440600	4.30420800
H	4.42421400	7.53938400	2.55044400
C	1.72367000	1.90572300	7.21082200
C	1.78721900	1.60084500	5.69752000
C	2.71839800	0.47458700	5.31508200
C	2.29874300	-0.85887800	5.43983500
C	4.01097800	0.72485500	4.83852000
C	3.14517200	-1.91441600	5.10202000
C	4.86551900	-0.32948000	4.50917600
C	4.43642600	-1.65043100	4.64059600
H	1.70918100	0.98689400	7.80369900
H	0.77584000	1.34731300	5.35845700
H	2.06657200	2.51257800	5.15801000
H	1.29613600	-1.06838400	5.80587500
H	4.34560900	1.75217600	4.72030900
H	2.79725000	-2.94011800	5.18133300
H	5.86864400	-0.11886500	4.14972200
H	5.09796000	-2.46864500	4.37951200
C	0.32403400	-4.94027900	-5.48744300
C	0.11131300	-3.82900200	-4.49125000
O	-0.02419700	-2.64918200	-4.81609700
H	0.14500700	-5.91227500	-5.01539200
N	0.04760100	-4.24496800	-3.18781500
C	0.15591200	-3.26433100	-2.14245600
C	1.62168200	-2.90666300	-1.89637900
O	2.48654700	-3.33155800	-2.66434700
H	0.46393300	-5.14421000	-2.97674900
H	-0.37014000	-2.36650700	-2.46635800
H	-0.32189400	-3.63153200	-1.23278800
N	1.88786900	-2.09781500	-0.85803500
C	3.24919100	-1.73069300	-0.51747200
C	3.99562700	-2.95344800	0.05028900
O	3.38290600	-3.92009100	0.52984900
C	3.27945700	-0.54252400	0.47122000
C	2.77661700	0.73865700	-0.19388300
C	2.51913500	-0.83059900	1.76818000
H	1.12732200	-1.78595300	-0.27369700
H	3.75452000	-1.43286900	-1.44056300
H	4.33379400	-0.40460100	0.73084600
H	2.84407300	1.58214200	0.49776900
H	3.35761600	0.98099200	-1.08760000
H	1.73730900	0.62944600	-0.51790200
H	2.67784900	-0.02439400	2.48616000
H	1.43739600	-0.89674100	1.59598600
H	2.84435700	-1.75945700	2.23737500
N	5.32998900	-2.86062200	0.00086800
C	6.23398400	-3.86550100	0.57262300
C	5.91225600	-5.28825900	0.05868500
O	5.84834400	-6.24283000	0.82734300
C	6.23558400	-3.81588400	2.09648100
H	5.73850700	-2.08816200	-0.51656700
H	7.22116300	-3.61108100	0.17458300
H	6.91776300	-4.56938100	2.49215100
H	6.54729400	-2.82456700	2.43724100
H	5.23662000	-4.03272100	2.47995000

N	5.78517200	-5.37041400	-1.28922500
C	5.38576300	-6.60034500	-1.95148900
C	3.95069300	-6.48230300	-2.49087000
O	2.97781500	-6.24575500	-1.48036200
H	5.79742400	-4.51800400	-1.85173600
H	5.44502200	-7.39766800	-1.20792600
H	3.92080800	-5.69092200	-3.25440800
H	3.67026600	-7.42455600	-2.97513700
H	3.26763100	-5.48408600	-0.95062700
C	0.19296000	1.03109100	-5.83563900
C	0.14529400	0.51167400	-4.40907800
S	1.76571400	-0.06952400	-3.75190900
H	-0.83483900	1.26257200	-6.14599400
H	-0.18378000	1.30286100	-3.73074300
H	-0.52672900	-0.34303900	-4.32314900
H	1.66427100	-1.32084000	-4.24676100
C	8.96780500	-2.34822800	-4.24714700
C	8.21251600	-3.30453000	-3.32355200
O	8.04430300	-2.77331500	-2.00746100
H	9.15682000	-2.83142600	-5.21327000
H	7.24968400	-3.59381400	-3.75598300
H	8.79832100	-4.21974500	-3.19094500
H	7.47114000	-1.99429500	-2.05502000
C	7.98611900	1.35008400	-4.34365900
C	7.51281900	2.08250700	-3.07224700
C	6.10066300	1.69013500	-2.58357400
C	5.97530800	0.19848200	-2.42048000
O	6.33304300	-0.39606600	-1.41156300
O	5.51050400	-0.48804100	-3.46677300
H	7.15370000	1.15623200	-5.02940200
H	8.22386300	1.90593500	-2.25896600
H	7.49497200	3.16421100	-3.23935200
H	5.90792300	2.15391200	-1.61759700
H	5.35009200	2.04742700	-3.29237300
C	-5.43663400	4.88693800	-4.21557700
C	-4.93669900	5.35076900	-2.84108700
C	-3.41853400	5.63566100	-2.83005900
C	-5.34817400	4.34958600	-1.75672300
C	-2.52418300	4.49097600	-3.32137100
H	-5.02006500	3.90397600	-4.46697000
H	-5.43510500	6.30358800	-2.61333800
H	-3.22433400	6.52179900	-3.44907400
H	-3.12499700	5.90526300	-1.80728500
H	-5.15907800	4.73272100	-0.75132200
H	-6.41529700	4.11370300	-1.82185100
H	-4.79860500	3.40882900	-1.86412900
H	-1.46839100	4.76233400	-3.22806400
H	-2.67602400	3.58259700	-2.73257300
H	-2.71461300	4.25375200	-4.37313000
C	-7.63179000	0.91076900	-6.48601600
C	-6.31043100	0.84434600	-5.70826000
C	-6.08026800	-0.49515600	-4.98928600
C	-6.11212600	-1.69345900	-5.94453600
C	-4.76623100	-0.46945500	-4.20634000
H	-7.57958200	0.34132200	-7.41920700
H	-5.46518600	1.04817300	-6.38104100
H	-6.29582000	1.64153600	-4.95331500
H	-6.89527400	-0.61903700	-4.26183300
H	-5.94486400	-2.62852600	-5.39964800
H	-7.07254100	-1.77921400	-6.46028900
H	-5.32816100	-1.60493300	-6.70716500
H	-4.63836400	-1.39364500	-3.63548800
H	-3.90688600	-0.36716300	-4.88167500
H	-4.74528300	0.37068900	-3.50458300
C	-10.20844700	0.44636400	-3.71976400
C	-10.26216600	1.20801800	-2.38456800
C	-9.03035300	0.88474000	-1.55906700
C	-9.10487000	-0.00259000	-0.47740700
C	-7.77128200	1.38747400	-1.91756000
C	-7.96041800	-0.39542500	0.21964500
C	-6.62360300	0.99938300	-1.22538800
C	-6.71357100	0.09881400	-0.16351300
H	-9.88315900	-0.58075200	-3.50607500
H	-10.33343000	2.28754100	-2.56312600
H	-11.15759800	0.92005400	-1.82379800
H	-10.07262200	-0.40328500	-0.18727000
H	-7.68731600	2.08549700	-2.74659800
H	-8.02699600	-1.09799000	1.04477200
H	-5.66114500	1.41114900	-1.50847000
H	-5.81786500	-0.22526700	0.35529400
C	-2.25213700	-2.76663100	7.73034100
C	-2.25249600	-2.18133200	6.32453600
O	-2.47845300	-2.85746000	5.31862200
H	-2.07619100	-3.83993300	7.67834500
N	-2.01566600	-0.83741900	6.26736900
C	-2.23825700	-0.00676900	5.07346900
C	-3.73713000	-0.02891300	4.70069400
O	-4.52403100	0.76291100	5.22718300
C	-1.28085800	-0.33326800	3.92741100
H	-1.97692500	-0.34285200	7.14644600
H	-2.06280300	1.02285900	5.38895400
H	-1.51003500	0.32362400	3.08453300
H	-0.24786300	-0.15833600	4.23363000
H	-1.35920000	-1.37876100	3.62582300
N	-4.10706900	-0.94522000	3.78722500
C	-5.48476000	-1.19770900	3.40314500
C	-5.75365800	-2.67117100	3.08767500
O	-6.70201900	-2.98393800	2.37165700
H	-3.40851400	-1.58256200	3.44092800
H	-6.13252900	-0.86691500	4.22028100
H	-5.76562800	-0.62930100	2.51341900
N	-4.91026500	-3.57053400	3.64790600
C	-5.06237100	-4.99797000	3.40958200
H	-4.19001600	-3.27679900	4.29798400
H	-5.04801600	-5.21206000	2.33780600
C	-8.61777200	-5.45411800	-0.13940700
C	-7.22503500	-5.07702700	-0.65835800
C	-7.04140800	-3.58184100	-0.96546700
C	-5.56691000	-3.25770000	-1.22207200
C	-7.89871100	-3.10415800	-2.14379400
H	-9.39483800	-5.18467400	-0.86355500
H	-6.48176800	-5.36506000	0.09635800
H	-6.99217200	-5.66219900	-1.56041100
H	-7.34060400	-3.02886500	-0.06949800
H	-5.44038800	-2.18696600	-1.40768200
H	-4.95179400	-3.52135500	-0.35656200
H	-5.18825900	-3.80214700	-2.09802900
H	-7.75591000	-2.03253400	-2.31319400
H	-7.62518800	-3.63447200	-3.06543300
H	-8.96658400	-3.26631100	-1.96914200

C	1.53307600	-6.08748000	3.01327700
O	0.47547200	-6.52929600	3.45264400
N	1.72354800	-4.73103000	2.82897500
C	0.52990200	-3.90730100	2.85503200
C	-0.27865200	-4.12702000	1.56286400
S	-2.08218500	-3.87074100	1.74902700
O	5.12137300	-3.33563000	-3.20962500
C	-1.66457900	-0.66283200	-0.59672900
C	-1.40072900	-1.34348800	0.53646500
C	-2.41918300	-2.30178100	1.05883600
O	-3.58289100	-1.88787400	1.08209100
C	-1.27720300	0.78148800	-0.68882700
C	-2.43594000	1.64891200	-0.14781800
O	-2.49737800	2.83491800	-0.37556500
O	-3.36615300	1.06553900	0.63724600
C	-2.61043200	-1.14179500	-1.65832600
H	-3.52798900	-0.54587800	-1.65810400
H	-2.15825700	-1.00520400	-2.64629500
H	-2.89435100	-2.18625700	-1.54219800
H	-0.40767900	1.01982600	-0.06734100
H	-0.69496500	-0.95595300	1.26646900
H	-1.06972700	1.13008700	-1.70205600
H	-0.19897000	-5.17624200	1.27536600
H	0.10546300	-3.52214900	0.74195800
H	-0.06983800	-4.20364500	3.71685600
H	0.80863500	-2.85764400	2.97174500
H	2.45349100	-4.45266400	2.17542800
C	2.68215400	-6.96923400	2.59340100
H	2.64376900	-7.11631900	1.50672400
H	3.65885100	-6.52574900	2.80429600
H	2.58948800	-7.93789000	3.08381200
H	-0.38068000	-4.86923800	-6.32246800
H	1.36437900	-4.96112900	-5.82873500
H	6.07006500	-6.83316600	-2.77405500
H	8.39533600	-1.43837100	-4.45560600
H	9.93736400	-2.13862100	-3.78342400
H	8.44160600	0.38639100	-4.09327500
H	8.68018400	2.00174100	-4.88473700
H	0.79400500	2.45472900	7.39349600
H	2.56374700	2.52695000	7.53850300
H	6.77498500	6.72340500	-0.23330500
H	4.95788400	6.79311500	-0.17022900
H	3.25369400	9.29074000	-1.83377400
H	4.03802800	7.72978200	-1.48445700
H	3.15022900	8.72512700	2.94330900
H	4.21109300	8.06206200	4.25305500
H	-2.22404300	8.17788400	3.61429200
H	-2.52434200	7.17710600	2.16891900
H	-5.13602700	5.57695200	-5.01091800
H	-6.52500600	4.76841100	-4.19627100
H	-8.42076100	0.46044400	-5.87464400
H	-7.87544600	1.94669700	-6.74389500
H	-11.15757100	0.32370500	-4.25190000
H	-9.43202300	0.85535300	-4.37459000
H	-8.83433100	-4.91113400	0.78650500
H	-8.68888900	-6.53718600	0.00530100
H	-4.22375000	-5.50668900	3.89631900
H	-6.01264000	-5.35621600	3.81902800
H	-3.24007000	-2.57806300	8.16331200
H	-1.48841600	-2.28219600	8.34766200
H	0.56192900	0.28239600	-6.54437500
H	0.73912300	1.97809400	-5.89922800
H	4.16426300	-3.33292200	-3.02020900
H	5.33544500	-2.41141500	-3.39785500
H	5.17434200	0.09877800	-4.20826500
O	-4.12772700	4.68834400	1.44601300
H	-4.07705300	4.07282600	2.19988000
H	-3.74474600	4.16701900	0.72405600
O	4.33098400	0.86444700	-5.35285600
H	4.39981300	1.78813200	-5.62153300
H	3.40105200	0.71472500	-5.06594000
O	-3.45817600	2.51971600	3.19289600
H	-4.03733800	2.22510300	3.91700500
H	-3.69466400	1.96184600	2.43594100

E:S_p-anti (+11.0)

C	2.94642000	8.29119000	-1.50319300
C	2.11339700	7.64210400	-2.62261200
C	1.93260200	6.14550800	-2.48337500
C	0.98081800	5.61527600	-1.60204700
C	2.69107200	5.25325600	-3.25317600
C	0.77145400	4.23980200	-1.51532700
C	2.49334600	3.87409800	-3.16154000
C	1.52620900	3.36362700	-2.29612600
H	2.45830200	8.19122500	-0.52989800
H	1.12532100	8.11700200	-2.65699100
H	2.58743200	7.85036200	-3.58868600
H	0.39540300	6.28996200	-0.98471300
H	3.43173500	5.64954300	-3.94329200
H	0.00075000	3.85148900	-0.85874100
H	3.07749300	3.19512300	-3.77701100
H	1.36921400	2.29449100	-2.22390200
C	5.78776000	6.26675600	0.05292800
C	5.65121000	4.90965200	-0.64402300
C	4.48251400	4.08907300	-0.08989900
S	4.73754500	3.41953700	1.60008400
C	6.04211400	2.18380500	1.27629700
H	5.84771500	6.11907800	1.13614000
H	5.46394500	5.06291000	-1.71614700
H	6.58669900	4.34023400	-0.57432300
H	3.59463900	4.71804100	0.00057900
H	4.22714100	3.25449200	-0.74635200
H	6.20746500	1.64591900	2.21152400
H	5.74256900	1.46244200	0.51147800
H	6.98115500	2.65602400	0.97809700
C	-2.13146900	7.09021100	3.39221700
C	-0.68660200	6.64887100	3.54383400
O	0.03203400	7.15223300	4.41451800
H	-2.76674800	6.41013900	3.97271500
N	-0.22518700	5.65321400	2.73976600
C	1.11102100	5.07787300	2.99350600
C	2.22545900	6.05471100	2.57186700
O	2.57373700	6.17693900	1.39679600
C	1.12789100	3.79153600	2.15977100
C	-0.34999800	3.44459400	1.96382900
C	-1.02209200	4.81555200	1.83440200
H	1.19967400	4.87358200	4.06662900
H	1.58965800	4.01444300	1.19545000
H	1.70982200	3.00233100	2.63866000

H	-0.50845300	2.83407600	1.07800900
H	-0.75678800	2.90691000	2.82752400
H	-0.95305400	5.18734200	0.80429600
H	-2.07281900	4.81127000	2.12073500
N	2.77852700	6.72795500	3.60738100
C	3.69913800	7.83460400	3.37683300
H	2.15191800	6.81778600	4.40169200
H	4.41043600	7.55382600	2.60050700
C	1.87835000	1.80332100	7.22992800
C	1.92323000	1.51758700	5.71386600
C	2.89429200	0.43616400	5.29926800
C	2.57276400	-0.91419200	5.50656000
C	4.12081900	0.74486500	4.69942100
C	3.44406300	-1.93069600	5.11783900
C	5.00388400	-0.26977400	4.32336800
C	4.66806000	-1.60786600	4.52843800
H	1.88874100	0.87706200	7.81134600
H	0.91706100	1.22456800	5.39037400
H	2.15438000	2.44538000	5.17815200
H	1.62471300	-1.17000200	5.97371900
H	4.38020000	1.78495200	4.51960000
H	3.16661700	-2.97113000	5.25626400
H	5.95663800	-0.01599400	3.86798400
H	5.34953300	-2.39503600	4.22751800
C	0.24525900	-4.85047800	-5.54244400
C	0.05012200	-3.75779200	-4.51798400
O	-0.04384600	-2.56721100	-4.81817900
H	0.08668400	-5.83330400	-5.08563000
N	-0.04424800	-4.20022200	-3.22623900
C	0.07204400	-3.25577200	-2.14564400
C	1.53754800	-2.86672900	-1.93404300
O	2.40642400	-3.31600800	-2.68390200
H	0.33437600	-5.11924300	-3.03358100
H	-0.50878400	-2.36512100	-2.39081100
H	-0.34234100	-3.68636200	-1.23104900
N	1.80457600	-2.01835300	-0.92703200
C	3.16782500	-1.64331700	-0.60347600
C	3.94308100	-2.86917600	-0.07674900
O	3.35877400	-3.83904500	0.42726700
C	3.20729300	-0.47639800	0.41085000
C	2.66780000	0.81281000	-0.20935300
C	2.49491300	-0.80213300	1.72611200
H	1.04090300	-1.67030700	-0.36740800
H	3.65488400	-1.31589300	-1.52722900
H	4.26751500	-0.32662600	0.63941400
H	2.76578300	1.64395200	0.49353400
H	3.20818100	1.07188000	-1.12337600
H	1.61371400	0.70985000	-0.48525200
H	2.64023000	0.00889800	2.44199000
H	1.41287200	-0.91948200	1.58638700
H	2.87832000	-1.71570700	2.18108600
N	5.27493400	-2.76487500	-0.18792400
C	6.22097100	-3.74892600	0.35038500
C	5.92837600	-5.18003100	-0.15719800
O	5.90834600	-6.13580200	0.61270400
C	6.27704500	-3.70214000	1.87326000
H	5.65291000	-1.98043300	-0.71002100
H	7.18744000	-3.46743000	-0.07936000
H	6.99248400	-4.43859800	2.24149500
H	6.57744400	-2.70351700	2.20269400

H	5.30010000	-3.94292800	2.29644300
N	5.78390400	-5.26833400	-1.50331900
C	5.40885600	-6.51110900	-2.15726300
C	3.95366000	-6.44035800	-2.64950400
O	3.00985300	-6.23962800	-1.60594100
H	5.74894300	-4.41540800	-2.06449600
H	5.51621800	-7.30616500	-1.41660300
H	3.87174700	-5.64838600	-3.40909400
H	3.68852100	-7.39038600	-3.12714900
H	3.27721500	-5.45433500	-1.10070700
C	0.04134000	1.12390700	-5.78859100
C	0.04335900	0.57024900	-4.37240400
S	1.69936400	0.01127000	-3.78550200
H	-0.99773500	1.34687100	-6.06606600
H	-0.28635500	1.33073900	-3.66026200
H	-0.60321300	-0.30412100	-4.28815400
H	1.57995200	-1.25006800	-4.25288000
C	8.88807000	-2.18238100	-4.46782200
C	8.15200000	-3.14533700	-3.53452800
O	8.00769400	-2.61643700	-2.21476700
H	9.05509800	-2.65411900	-5.44337100
H	7.18100800	-3.43311800	-3.95043900
H	8.73944900	-4.06120800	-3.41501400
H	7.41492300	-1.85146100	-2.24614900
C	7.86448900	1.50577700	-4.47950800
C	7.38677200	2.21010400	-3.19446200
C	5.97170800	1.80120700	-2.72921600
C	5.86862800	0.30715200	-2.56979900
O	6.24287100	-0.28097900	-1.56298800
O	5.41281700	-0.38623900	-3.61428200
H	7.02640000	1.30831200	-5.15794500
H	8.09131500	2.01707300	-2.37928300
H	7.36759200	3.29506700	-3.33886100
H	5.75676300	2.26014500	-1.76537200
H	5.22890500	2.14903200	-3.45075900
C	-5.58857300	4.88944900	-3.97001000
C	-5.05143400	5.50230100	-2.66521700
C	-3.53473800	5.78311000	-2.74831000
C	-5.41168300	4.69401700	-1.41864600
C	-2.63823200	4.55735300	-2.95972900
H	-5.13923000	3.91650100	-4.19156700
H	-5.54015500	6.48212400	-2.56183300
H	-3.36035700	6.49832400	-3.56310900
H	-3.22519500	6.29406700	-1.82631100
H	-5.11878900	5.26397800	-0.52972500
H	-6.48523300	4.48968400	-1.36067800
H	-4.88636500	3.73743100	-1.38355100
H	-1.58840900	4.85990400	-3.01526000
H	-2.72727800	3.84008000	-2.13920800
H	-2.87773900	4.03745300	-3.89329800
C	-7.79515400	0.92663100	-6.25237400
C	-6.43831100	0.86327300	-5.54521800
C	-6.09129900	-0.51395800	-4.95800800
C	-6.14243000	-1.63621000	-6.00050200
C	-4.71708900	-0.46757800	-4.28820300
H	-7.77428800	0.37868900	-7.20013800
H	-5.64445400	1.16807100	-6.24243000
H	-6.42125100	1.59451100	-4.72637200
H	-6.83582200	-0.73774400	-4.18029100
H	-5.86724300	-2.59508100	-5.54914500

H	-7.14030100	-1.74961200	-6.43318700
H	-5.44080800	-1.43910700	-6.82097300
H	-4.47988300	-1.42313600	-3.81356300
H	-3.93079800	-0.25215600	-5.02344400
H	-4.68355700	0.31108600	-3.52033800
C	-10.29881900	0.38844200	-3.43299600
C	-10.30185600	1.14699600	-2.09640700
C	-8.96875900	0.94188200	-1.39852700
C	-8.84862600	0.05682500	-0.31900800
C	-7.80367400	1.55284400	-1.88442000
C	-7.60971900	-0.21419600	0.26619300
C	-6.56134000	1.28290000	-1.30874200
C	-6.46134100	0.40348700	-0.22955500
H	-9.95452900	-0.63576900	-3.23784700
H	-10.48562000	2.21521100	-2.26479800
H	-11.10978700	0.78247500	-1.45331500
H	-9.73782300	-0.44012300	0.05966400
H	-7.86787900	2.23946100	-2.72525900
H	-7.53393100	-0.91927900	1.08883200
H	-5.67041200	1.76116400	-1.70124100
H	-5.49773500	0.21001000	0.22905800
C	-2.03308300	-2.92093700	7.76761000
C	-2.03776800	-2.36263300	6.35397300
O	-2.28241800	-3.05563900	5.36612700
H	-1.85010700	-3.99401000	7.72019500
N	-1.76023000	-1.02943900	6.26473000
C	-1.85534300	-0.23893000	5.03067800
C	-3.30908000	-0.19217300	4.51665100
O	-4.05965200	0.71717500	4.90403300
C	-0.83239500	-0.65119100	3.97185300
H	-1.68865000	-0.51118200	7.12722800
H	-1.65743600	0.79191000	5.33182100
H	-0.96328200	-0.02153300	3.08881300
H	0.18194500	-0.52416600	4.35000200
H	-0.95037200	-1.69776000	3.69001000
N	-3.67096900	-1.15823600	3.66029700
C	-5.00155900	-1.31757400	3.09896500
C	-5.47319600	-2.77174200	3.01482600
O	-6.46205000	-3.04418000	2.33912000
H	-2.97572000	-1.85144200	3.41982200
H	-5.70191900	-0.73160400	3.69701900
H	-5.03374800	-0.93778600	2.07580100
N	-4.73768500	-3.68674700	3.68543900
C	-4.92286600	-5.11303700	3.47949800
H	-3.97738400	-3.39977700	4.29010700
H	-4.93272400	-5.33041300	2.40768800
C	-8.55814300	-5.55128900	0.01003100
C	-7.24850100	-5.10670000	-0.64384000
C	-7.18780200	-3.61217800	-1.01390000
C	-5.86920700	-3.29591100	-1.72726800
C	-8.36469000	-3.15562200	-1.88597300
H	-9.39979900	-5.30820800	-0.65060300
H	-6.40650100	-5.32469600	0.02459000
H	-7.08567600	-5.70185700	-1.55503400
H	-7.20478400	-3.04428800	-0.07697300
H	-5.77843300	-2.21897000	-1.90172000
H	-5.01241000	-3.61426400	-1.12990600
H	-5.82371100	-3.80005300	-2.70219300
H	-8.23263300	-2.11038900	-2.18258700
H	-8.43260400	-3.76080300	-2.79953700

H	-9.32381700	-3.22627400	-1.36470500
C	1.77738200	-6.08124300	3.04618500
O	0.74102400	-6.49866400	3.55172600
N	2.03930700	-4.72695800	2.93727100
C	0.87365400	-3.86462400	3.00898800
C	0.06701400	-3.96512000	1.69633200
S	-1.74735400	-4.04744000	1.93963700
O	5.01538300	-3.24528800	-3.39789600
C	-1.94345700	-0.71146800	-0.42092600
C	-1.61181400	-1.58164200	0.55325300
C	-2.42605700	-2.77729800	0.87451800
O	-3.60655300	-2.86966700	0.57909800
C	-1.46111500	0.71455900	-0.33618900
C	-2.53125700	1.54185000	0.39526000
O	-2.92672700	2.62575100	-0.03551800
O	-2.93229300	0.97229700	1.49844800
C	-2.91594500	-0.98995700	-1.52258200
H	-3.87623700	-0.50371000	-1.31670100
H	-2.54142700	-0.56896000	-2.46086200
H	-3.12254400	-2.04964400	-1.64941300
H	-0.54186400	0.78794200	0.25425900
H	-0.87511500	-1.29124100	1.29351700
H	-1.28971600	1.17038800	-1.31340800
H	0.33152200	-4.89404300	1.18957300
H	0.31108900	-3.15327200	1.01327600
H	0.26579500	-4.19864700	3.85054400
H	1.18816900	-2.83751500	3.19793500
H	2.72218900	-4.46841600	2.22782700
C	2.82036400	-6.98412100	2.44466100
H	2.63354400	-7.07551600	1.36712300
H	3.83626700	-6.59182500	2.54342600
H	2.74961400	-7.97425400	2.89515200
H	-0.48022300	-4.77378100	-6.35897500
H	1.27718800	-4.85415200	-5.90896700
H	6.07549200	-6.72284900	-2.99977700
H	8.30092200	-1.27571700	-4.64737000
H	9.86626800	-1.96950600	-4.02416200
H	8.33627100	0.54331100	-4.25616100
H	8.53809900	2.17390200	-5.02628500
H	0.94752500	2.33873600	7.44396900
H	2.71940400	2.42837100	7.54754100
H	6.69538700	6.79757600	-0.25296400
H	4.87974500	6.84599400	-0.14497400
H	3.10878700	9.35112800	-1.72547000
H	3.91824600	7.79355600	-1.42106500
H	3.12736700	8.70679400	3.04267400
H	4.22674500	8.03443700	4.31537600
H	-2.22274200	8.08860200	3.83363500
H	-2.54736600	7.10820700	2.37965200
H	-5.31493900	5.59588900	-4.76077600
H	-6.67487700	4.75873200	-3.92646100
H	-8.56414600	0.45759700	-5.62971000
H	-8.05615700	1.96381100	-6.48717400
H	-11.25917300	0.26387600	-3.94407000
H	-9.54294100	0.81668200	-4.09944700
H	-8.75810300	-5.02594900	0.94971800
H	-8.61408900	-6.63734800	0.13843200
H	-4.06731500	-5.62043700	3.93739800
H	-5.85907200	-5.48875400	3.90565300
H	-3.01210800	-2.75079300	8.22734200

H	-1.25973900	-2.43841500	8.37436700
H	0.40104600	0.39101700	-6.51829200
H	0.57551700	2.07787500	-5.84964000
H	4.07242300	-3.26152400	-3.14702800
H	5.21257900	-2.31164100	-3.55548100
H	5.05889000	0.19429800	-4.35319100
O	-4.30798300	4.28577900	1.74538800
H	-5.08558100	4.66867500	1.31949600
H	-3.86057000	3.77875100	1.02983600
O	4.18932400	0.94186000	-5.48578300
H	4.24118000	1.86441400	-5.76173600
H	3.26845400	0.78357500	-5.17190200
O	-4.94055500	1.85604200	2.64200100
H	-4.80113300	1.58350500	3.57830100
H	-4.86563300	2.83551200	2.56783000

TS_{pc} (+34.2)

C	2.96628900	8.48020500	-1.29413100
C	2.16803900	7.85295700	-2.45155600
C	2.03672700	6.34637000	-2.37545800
C	1.14704400	5.74382300	-1.47578100
C	2.79160300	5.51678800	-3.21547800
C	1.00421700	4.35798200	-1.43369800
C	2.65860600	4.12666700	-3.17361200
C	1.75932600	3.54405100	-2.28033100
H	2.45184000	8.35600400	-0.33732300
H	1.16622000	8.29903600	-2.48052600
H	2.64810400	8.11498600	-3.40139600
H	0.57170000	6.36611800	-0.79756200
H	3.48372500	5.96999200	-3.92077600
H	0.30277900	3.90945700	-0.73914100
H	3.24242000	3.50275500	-3.84479800
H	1.65796200	2.46589800	-2.22640800
C	5.76610400	6.42626600	0.29810700
C	5.66971900	5.08522900	-0.43827900
C	4.49059700	4.23062900	0.03703300
S	4.66284000	3.52640700	1.72426800
C	5.99652900	2.31069100	1.45072200
H	5.79209200	6.25141300	1.37856500
H	5.52631900	5.26456300	-1.51305000
H	6.61049100	4.52721700	-0.34477600
H	3.58782500	4.84031000	0.09700300
H	4.28666000	3.40901800	-0.65305900
H	6.17157900	1.81244500	2.40592500
H	5.71854500	1.55391100	0.71267400
H	6.92632500	2.79319700	1.13993900
C	-2.25219800	7.15249000	3.41724000
C	-0.81284200	6.70704400	3.59944400
O	-0.12559300	7.16587100	4.52066300
H	-2.90524000	6.45646200	3.95624400
N	-0.31549300	5.75575100	2.76191000
C	0.99868200	5.14805700	3.05842600
C	2.14733700	6.11654300	2.69932700
O	2.56371700	6.23470800	1.54594300
C	1.01208900	3.86123400	2.21574400
C	-0.47298000	3.55983400	1.96457800
C	-1.07481500	4.95701200	1.79136200
H	1.03966100	4.93275200	4.13210700
H	1.52320100	4.07305000	1.27445700
H	1.55200800	3.05544600	2.71683100
H	-0.64941100	2.92076600	1.09357900
H	-0.93325200	3.07080100	2.82990100
H	-0.89915900	5.33121400	0.77336500
H	-2.14602700	4.99435300	1.98555000
N	2.64138200	6.79646500	3.76378100
C	3.57403200	7.91046400	3.59413000
H	1.96841300	6.88203700	4.52127400
H	4.30848700	7.64820400	2.83313200
C	1.65690400	1.78608400	7.24805600
C	1.73106600	1.51427600	5.72974100
C	2.73891600	0.46525700	5.31839800
C	2.44182600	-0.89596100	5.48767900
C	3.97714900	0.81275400	4.76534600
C	3.35813500	-1.88211800	5.12337400
C	4.89556600	-0.17309100	4.39576400
C	4.59089100	-1.52231000	4.57305900
H	1.65118100	0.85104700	7.81588400
H	0.73812800	1.18695300	5.39786600
H	1.93732400	2.45249700	5.20237600
H	1.47730400	-1.17879900	5.90424000
H	4.21818000	1.86163900	4.61372400
H	3.11110400	-2.93121300	5.25745600
H	5.85311000	0.11289400	3.97087900
H	5.30241100	-2.28783300	4.28046800
C	0.42712900	-4.56853900	-5.71974100
C	0.22276500	-3.70003700	-4.50265200
O	-0.24169600	-2.55507300	-4.53074800
H	0.28455600	-5.60859200	-5.39329100
N	0.58623600	-4.32784400	-3.35017200
C	0.68765100	-3.64925200	-2.08950000
C	1.96573700	-2.83015600	-1.98332300
O	2.61401600	-2.55032200	-2.99526200
H	1.11615300	-5.18709600	-3.40740700
H	-0.14786700	-2.96256400	-1.98684500
H	0.63369800	-4.38075800	-1.28101300
N	2.24595400	-2.37371900	-0.74174600
C	3.54251400	-1.79126300	-0.40774300
C	4.38119700	-2.91977400	0.20686100
O	3.82078900	-3.88120500	0.76545800
C	3.42011000	-0.56784600	0.53473400
C	2.91507500	0.66388200	-0.21963500
C	2.53590200	-0.85609900	1.75004400
H	1.85515900	-2.92337300	0.01562700
H	4.00663100	-1.48322200	-1.34430900
H	4.43634700	-0.36558200	0.89355500
H	2.89524500	1.53496300	0.44168100
H	3.55295100	0.90417300	-1.07606400
H	1.90702800	0.49128100	-0.60934500
H	2.59075100	-0.03355200	2.46494600
H	1.48837600	-0.95762000	1.44263600
H	2.83882200	-1.76345100	2.27606700
N	5.70684000	-2.80474500	0.07443100
C	6.63470500	-3.85988300	0.50127000
C	6.25243000	-5.22161500	-0.13113100
O	6.29315300	-6.26257700	0.51446500
C	6.73791900	-3.95527200	2.01989200
H	6.06954200	-2.04424300	-0.49185800
H	7.59719100	-3.57771200	0.06381900
H	7.42414800	-4.75786200	2.29286200

H	7.10338900	-3.00887600	2.42844800
H	5.76168400	-4.18356200	2.45086800
N	5.92200700	-5.14486900	-1.44785700
C	5.49187100	-6.29666200	-2.22109800
C	4.09269600	-6.04755400	-2.80049200
O	3.11487200	-5.72458600	-1.81055800
H	5.91274300	-4.24328500	-1.92808100
H	5.49478300	-7.15757400	-1.54925100
H	4.15653000	-5.24117300	-3.54408400
H	3.74576800	-6.95327000	-3.31166100
H	3.56441200	-5.27112000	-1.07720200
C	0.21267200	1.40905200	-5.83148400
C	0.01194800	0.86589800	-4.42799900
S	1.49930200	0.17233800	-3.60962400
H	-0.78927300	1.66467200	-6.20921600
H	-0.34287800	1.66342800	-3.76992500
H	-0.72900500	0.06391000	-4.42475800
H	1.39479300	-1.06888900	-4.11781400
C	9.02532900	-1.90693700	-4.32381000
C	8.29431300	-2.93625600	-3.46393000
O	8.15092000	-2.52535500	-2.10362800
H	9.23355500	-2.33615500	-5.31171500
H	7.32527700	-3.19596800	-3.90254000
H	8.88621500	-3.85633900	-3.42316600
H	7.68636100	-1.67459700	-2.06999400
C	7.99126600	1.77795200	-4.28015900
C	7.51438800	2.44703500	-2.97614000
C	6.15119000	1.94680600	-2.44423400
C	6.12930000	0.44484900	-2.33835200
O	6.58994400	-0.17308300	-1.38601800
O	5.64173900	-0.22993000	-3.38456700
H	7.15428000	1.60653800	-4.96619800
H	8.26860900	2.30318000	-2.19561000
H	7.41408100	3.52759900	-3.11860600
H	5.98109800	2.35773900	-1.45038600
H	5.35114000	2.28868900	-3.10422400
C	-5.48080300	5.11841500	-4.09514200
C	-5.02303700	5.52583600	-2.68446300
C	-3.48707500	5.52065100	-2.51943800
C	-5.71749700	4.64948300	-1.63666900
C	-2.80277000	4.14901500	-2.61331900
H	-5.05192200	4.14999800	-4.37892000
H	-5.35117900	6.56145300	-2.51896600
H	-3.05000500	6.18815500	-3.27353300
H	-3.24518300	5.96707000	-1.54522200
H	-5.47009500	4.94465000	-0.61328500
H	-6.80626100	4.70883400	-1.73422100
H	-5.43871000	3.59960300	-1.76241600
H	-1.71681900	4.26383300	-2.52819300
H	-3.12400600	3.46650400	-1.81981000
H	-3.00575800	3.65905700	-3.57231500
C	-7.60575400	1.20455500	-6.53505100
C	-6.30592500	1.25854600	-5.69822500
C	-6.14088300	0.14560700	-4.65420800
C	-6.01849100	-1.24310900	-5.28753700
C	-4.94580800	0.44653700	-3.74348400
H	-7.49003200	0.65330900	-7.47150200
H	-5.42754000	1.27311400	-6.35888200
H	-6.28703600	2.21393600	-5.15937000
H	-7.03697800	0.15312800	-4.01969100
H	-5.93541500	-2.02031200	-4.52128500
H	-6.89063300	-1.48083700	-5.90456800
H	-5.12982800	-1.30070500	-5.92899400
H	-4.93183600	-0.23720500	-2.88967700
H	-3.99984500	0.35089900	-4.29395600
H	-4.99293800	1.46269400	-3.34074300
C	-10.19119000	0.59435500	-3.80525500
C	-10.31947800	1.32641300	-2.45518400
C	-9.15659100	0.99425900	-1.54045400
C	-9.28616900	0.00197900	-0.55874400
C	-7.90656900	1.60728400	-1.70168700
C	-8.19973800	-0.38905100	0.22564300
C	-6.81673700	1.22365200	-0.91888700
C	-6.95707500	0.21631600	0.03549600
H	-9.85322300	-0.43164300	-3.60938500
H	-10.38081600	2.40899100	-2.61685700
H	-11.25321700	1.02812000	-1.96665100
H	-10.24922000	-0.48233200	-0.41947300
H	-7.78439200	2.38955300	-2.44650400
H	-8.30887900	-1.17543500	0.96642500
H	-5.85287200	1.70206000	-1.04410700
H	-6.09501000	-0.09749400	0.61392000
C	-2.25449800	-2.95910000	7.55761800
C	-2.27042200	-2.30901000	6.18434500
O	-2.46751900	-2.93504800	5.14099400
H	-2.06016900	-4.02666400	7.46381800
N	-2.09719000	-0.94843600	6.18808200
C	-2.67176000	-0.14540900	5.09755600
C	-4.13156900	-0.62814100	4.90425300
O	-4.81763600	-0.86864000	5.89306800
C	-1.79584000	-0.09560400	3.84793100
H	-2.17157800	-0.52047900	7.10035000
H	-2.75271300	0.87302000	5.49159500
H	-2.16594900	0.68130800	3.17494200
H	-0.76653500	0.14289900	4.12707400
H	-1.79389400	-1.05476500	3.33220800
N	-4.58700500	-0.76038400	3.63165300
C	-5.88999500	-1.33981600	3.36354400
C	-5.90814500	-2.81005500	2.92372600
O	-6.84883800	-3.21417000	2.23796000
H	-3.97501600	-0.61638500	2.83648700
H	-6.47224600	-1.26539100	4.28579800
H	-6.40540300	-0.77458000	2.58739400
N	-4.91491700	-3.61733200	3.36513300
C	-5.00828800	-5.05600600	3.13430400
H	-4.19760700	-3.26483200	3.99012300
H	-4.98430100	-5.26862500	2.06089700
C	-8.53567400	-5.42082500	-0.45139200
C	-7.14862200	-4.96927800	-0.91949300
C	-7.02220600	-3.44330400	-1.07096700
C	-5.56086700	-3.03713600	-1.26611800
C	-7.87967400	-2.89124400	-2.21652600
H	-9.30914000	-5.13215200	-1.17225200
H	-6.40542500	-5.30383300	-0.18517700
H	-6.88711800	-5.45461500	-1.87122000
H	-7.35962700	-2.99899100	-0.12949800
H	-5.45907900	-1.94574400	-1.26981700
H	-4.92758800	-3.43907000	-0.46916400
H	-5.17721600	-3.42052400	-2.21916300
H	-7.74556000	-1.81016800	-2.31875000

H	-7.60276900	-3.36150500	-3.16893300
H	-8.94645900	-3.07120700	-2.05420100
C	1.64373200	-6.12465500	2.95815900
O	0.72554100	-6.69062200	3.54231700
N	1.70613100	-4.75449400	2.85759500
C	0.64896300	-3.94732700	3.42512700
C	-0.34191300	-3.36358400	2.40870900
S	-1.02912800	-4.68384600	1.32303000
O	5.23617900	-3.02562800	-3.20397200
C	-2.13947200	-1.58568500	-1.25670500
C	-1.86148300	-2.33810700	-0.06260300
C	-1.86598800	-3.84760400	-0.01590000
O	-2.41176600	-4.53934700	-0.85409300
C	-2.09723300	-0.14070300	-1.06267500
C	-3.15207100	0.33503100	0.08466700
O	-3.50554200	-0.52274700	0.93518500
O	-3.43611500	1.53536000	0.00890500
C	-2.59713200	-2.13979800	-2.53101500
H	-3.32292100	-1.48371900	-3.01179400
H	-1.70710300	-2.13682700	-3.19876300
H	-2.94191700	-3.16544700	-2.45050800
H	-1.14490500	0.18566000	-0.63036800
H	-1.07503200	-1.87607400	0.53407100
H	-2.77983300	-1.84899300	0.52853300
H	-2.31425100	0.43526400	-1.95986300
H	0.17176800	-2.62031400	1.79535400
H	-1.15725700	-2.89226000	2.95969500
H	0.11313300	-4.58186500	4.13250700
H	1.07219000	-3.09627000	3.96948300
H	2.43783900	-4.33482500	2.29379500
C	2.76803900	-6.88403200	2.28877100
H	2.39047100	-7.31310200	1.35451300
H	3.63792100	-6.26882300	2.04728200
H	3.06344200	-7.71205000	2.93522600
H	-0.27421900	-4.47499500	-6.55561100
H	1.46931800	-4.56186700	-6.05514300
H	6.18393400	-6.48743100	-3.04787200
H	8.44148200	-0.99780100	-4.50005200
H	9.98950500	-1.70243300	-3.84639300
H	8.45954700	0.81167900	-4.06467100
H	8.67958300	2.46030700	-4.79033200
H	0.71832600	2.31425200	7.44709200
H	2.48608500	2.40540400	7.60584700
H	6.68075600	6.96620700	0.03289000
H	4.86227200	7.00756300	0.08733400
H	3.13215900	9.54534500	-1.48662100
H	3.93685300	7.98308800	-1.19448800
H	3.00985700	8.78903900	3.26365000
H	4.07263400	8.08952000	4.55268900
H	-2.35972300	8.14015000	3.87900300
H	-2.63755000	7.19354200	2.39329900
H	-5.18551100	5.84303500	-4.86043800
H	-6.56738800	4.98315100	-4.08706300
H	-8.39199500	0.71951400	-5.94674700
H	-7.86312800	2.24640400	-6.75290000
H	-11.13566400	0.47988100	-4.34772700
H	-9.41717500	1.04005800	-4.43845900
H	-8.76664600	-4.91777900	0.49337300
H	-8.59359300	-6.50949300	-0.35093900
H	-4.16525000	-5.57170800	3.60577300
H	-5.95555400	-5.44346900	3.52360700
H	-3.24760200	-2.80124400	7.99156700
H	-1.50153400	-2.48836100	8.19836500
H	0.59607400	0.69425600	-6.56733800
H	0.74519500	2.36539000	-5.85431200
H	4.26542700	-2.92476100	-3.19267500
H	5.55029600	-2.11486100	-3.30292000
O	-3.96348900	2.28413700	2.61700100
H	-4.67600300	1.73805100	2.97669000
H	-3.87765400	1.99182800	1.68302600
O	4.15976200	1.22676200	-4.95290400
H	4.16163900	1.06369400	-5.90500400
H	3.26128800	0.96212200	-4.63341300
O	-4.27244000	4.85964300	1.55166100
H	-4.33166500	4.03916100	2.08233500
H	-4.00221100	4.53064400	0.68486800
H	5.15543100	0.34982000	-4.04751100

Int_p (-9.2)

C	2.80099600	8.37008200	-1.38090000
C	1.95185800	7.73119400	-2.49455300
C	1.72876500	6.23935000	-2.34386700
C	0.73850500	5.74004900	-1.48644800
C	2.48273200	5.31996100	-3.08584200
C	0.49494100	4.36971300	-1.38852000
C	2.25214000	3.94570900	-2.98655800
C	1.25398000	3.46639800	-2.13684800
H	2.31807500	8.26086900	-0.40531400
H	0.97685100	8.23242600	-2.53090000
H	2.42969300	7.91904400	-3.46310900
H	0.14755600	6.43317500	-0.89465600
H	3.24878700	5.68922300	-3.76336800
H	-0.30449700	4.01000800	-0.74886200
H	2.83579700	3.25415200	-3.58819400
H	1.06909500	2.40098300	-2.05303200
C	5.63209200	6.31210100	0.14924700
C	5.50256400	4.96803300	-0.57462700
C	4.32618000	4.12935800	-0.06546800
S	4.52829000	3.41798100	1.61610800
C	5.91592800	2.26497700	1.33933300
H	5.68461400	6.14403800	1.23013800
H	5.33308000	5.14298700	-1.64629700
H	6.43775200	4.39794200	-0.50327800
H	3.42975000	4.74684500	0.01785600
H	4.09788200	3.31118800	-0.75166800
H	6.01060800	1.66368000	2.24480200
H	5.72178800	1.58896400	0.50342100
H	6.85701500	2.79211900	1.16552100
C	-2.30055000	7.08806900	3.46901800
C	-0.84909200	6.66525800	3.63042900
O	-0.12742800	7.21849000	4.46895000
H	-2.93481600	6.40020700	4.04191800
N	-0.40023300	5.63597700	2.87065300
C	0.93931900	5.06166300	3.11219400
C	2.05843900	6.03024800	2.67663100
O	2.41595400	6.13448200	1.50384500
C	0.92360000	3.75794500	2.30166200
C	-0.56187600	3.38460900	2.25816100

C	-1.23678800	4.74173200	2.05817300
H	1.04239400	4.86529700	4.18681900
H	1.29393200	3.97323100	1.29617400
H	1.56990500	2.99607700	2.74122300
H	-0.83063500	2.68624500	1.46797500
H	-0.87721700	2.95130000	3.21541900
H	-1.22320900	5.03602600	1.00640600
H	-2.27558600	4.75663800	2.38353600
N	2.60650600	6.71423800	3.71127100
C	3.53140800	7.82195000	3.49285000
H	1.96403200	6.82069800	4.49108800
H	4.24284300	7.54878900	2.71395800
C	1.68296500	1.72390800	7.22539700
C	1.75059600	1.43407300	5.71315600
C	2.91132100	0.55182300	5.31752100
C	2.79416000	-0.84318200	5.38543300
C	4.13801500	1.09953600	4.92245200
C	3.87320400	-1.67113400	5.07420400
C	5.22121900	0.27402600	4.61460700
C	5.09259600	-1.11351800	4.68605900
H	1.69433200	0.79377800	7.80157800
H	0.81501300	0.94709900	5.41408200
H	1.79989900	2.38134100	5.16374400
H	1.84677100	-1.28194700	5.69113500
H	4.24453900	2.17898600	4.85496600
H	3.76526400	-2.74994300	5.13828700
H	6.17015800	0.71637300	4.32669900
H	5.93302400	-1.75579700	4.44242300
C	0.09542700	-4.68913700	-5.67474300
C	-0.07877200	-3.77002200	-4.48227900
O	-0.50146800	-2.61748200	-4.54449700
H	-0.04720900	-5.71857800	-5.31754900
N	0.27112700	-4.37225900	-3.30040300
C	0.43856300	-3.61632700	-2.08867600
C	1.77415200	-2.87733500	-2.07326800
O	2.38882500	-2.69253000	-3.12572800
H	0.80855700	-5.22843900	-3.33731400
H	-0.35850100	-2.87334000	-2.02587100
H	0.35625500	-4.28199400	-1.22534600
N	2.15886700	-2.38171300	-0.86767100
C	3.50779700	-1.85940600	-0.65617700
C	4.31677100	-2.99127700	-0.01530500
O	3.73232800	-3.94210000	0.53978200
C	3.57677400	-0.55649100	0.17942300
C	2.85920500	0.59516400	-0.52490900
C	3.07349100	-0.70119300	1.61655500
H	1.82566500	-2.92478900	-0.07940700
H	3.92321200	-1.65441000	-1.64132200
H	4.64599100	-0.32401400	0.22510100
H	2.91781900	1.50104000	0.08411700
H	3.29483000	0.80907300	-1.50491500
H	1.80558200	0.35612700	-0.69338100
H	3.35942000	0.17625000	2.20107500
H	1.98285000	-0.75843800	1.63945100
H	3.47937500	-1.58017000	2.12309200
N	5.64509200	-2.90230800	-0.10623100
C	6.52691200	-3.98086500	0.35502700
C	6.09641100	-5.33974800	-0.25017200
O	6.13772300	-6.37336900	0.40734100
C	6.60472700	-4.04563800	1.87718400

H	6.03926500	-2.16505900	-0.68139600
H	7.50522000	-3.74540200	-0.07395500
H	7.26353600	-4.86153200	2.17695200
H	6.98891700	-3.10084800	2.27203600
H	5.61582400	-4.23641500	2.29744200
N	5.72440500	-5.27124700	-1.55523400
C	5.24066800	-6.42182700	-2.29783200
C	3.81929600	-6.16486200	-2.81516900
O	2.89622100	-5.81750500	-1.78236700
H	5.72159200	-4.37842400	-2.04910500
H	5.26283800	-7.27633700	-1.61825000
H	3.85340000	-5.37390900	-3.57661400
H	3.43744900	-7.07493100	-3.29190900
H	3.37367700	-5.29647100	-1.11320300
C	-0.09680300	1.28825900	-5.81054700
C	-0.22064500	0.74047000	-4.40400000
S	1.32687900	0.07759900	-3.68636300
H	-1.11449100	1.53922500	-6.14635700
H	-0.55125300	1.52394400	-3.72032300
H	-0.93863200	-0.07878600	-4.35828800
H	1.21000100	-1.16034800	-4.19897800
C	8.73756000	-2.05765900	-4.51233300
C	8.03932900	-3.09202800	-3.63516900
O	7.92292300	-2.67303500	-2.27471500
H	8.92248500	-2.48632800	-5.50532500
H	7.06479200	-3.36665000	-4.05008300
H	8.64477400	-4.00381800	-3.60518100
H	7.50623700	-1.79703100	-2.24565500
C	7.72016900	1.63179200	-4.46082100
C	7.26858900	2.31658900	-3.15760900
C	5.91485500	1.81066900	-2.61332800
C	5.93108700	0.30907000	-2.51290500
O	6.46965000	-0.29231600	-1.59025100
O	5.40112300	-0.37777400	-3.52704200
H	6.86870100	1.45927200	-5.12849400
H	8.03231100	2.17906500	-2.38537500
H	7.16585300	3.39541300	-3.31079400
H	5.74454000	2.21872200	-1.61818000
H	5.10737300	2.13952100	-3.27002100
C	-5.72887600	5.03077700	-3.94825600
C	-5.14488900	5.36877900	-2.57471400
C	-3.60095200	5.45257600	-2.60268900
C	-5.64726700	4.35705500	-1.53885800
C	-2.87891400	4.23479600	-3.19841900
H	-5.34110700	4.06007000	-4.28354600
H	-5.51003100	6.36057500	-2.27416900
H	-3.30220400	6.34064600	-3.17366900
H	-3.24475400	5.61420800	-1.57798100
H	-5.23081300	4.54128900	-0.54492100
H	-6.73872000	4.37429000	-1.46253700
H	-5.37270400	3.33792700	-1.83332800
H	-1.80296200	4.29911100	-3.01393500
H	-3.23192600	3.28799100	-2.77122900
H	-3.02971000	4.16331400	-4.27948300
C	-7.93178700	1.11397700	-6.31319300
C	-6.60866500	1.03797900	-5.53522500
C	-6.39733300	-0.30038100	-4.80747200
C	-6.41158500	-1.49843600	-5.76330400
C	-5.10404000	-0.27911300	-3.99128700
H	-7.87994900	0.57317400	-7.26279700

H	-5.75834100	1.23094000	-6.20468900
H	-6.58973300	1.83774900	-4.78269900
H	-7.23140800	-0.42023000	-4.10122500
H	-6.26503200	-2.43411600	-5.21406200
H	-7.35827200	-1.57741600	-6.30547500
H	-5.60612400	-1.41493600	-6.50380800
H	-4.98972100	-1.19889400	-3.41080100
H	-4.22876200	-0.18354000	-4.64646100
H	-5.09521300	0.56046700	-3.28840000
C	-10.44901900	0.52826200	-3.51516700
C	-10.50143200	1.25119500	-2.15789500
C	-9.28111700	0.89522100	-1.33009500
C	-9.37083600	-0.05238000	-0.30194500
C	-8.01956200	1.42753700	-1.63399900
C	-8.23886900	-0.47373100	0.39827100
C	-6.88512200	1.01057500	-0.93748500
C	-6.99023900	0.05428900	0.07163200
H	-10.11030000	-0.50078500	-3.33671900
H	-10.56340800	2.33590800	-2.30621800
H	-11.40370100	0.95503900	-1.61250200
H	-10.34075800	-0.47743200	-0.05742200
H	-7.92484500	2.16829400	-2.42410800
H	-8.32050800	-1.22479700	1.17832700
H	-5.91263700	1.42837800	-1.17088200
H	-6.09592300	-0.27741300	0.58588200
C	-2.23891700	-3.00290700	7.65882800
C	-2.34675000	-2.33114200	6.30021800
O	-2.64664900	-2.93832000	5.27307800
H	-2.05238300	-4.06870600	7.54167100
N	-2.16460800	-0.97330400	6.30509900
C	-2.69331200	-0.15122600	5.20806600
C	-4.17123400	-0.54453000	4.97692200
O	-4.95048700	-0.49840400	5.91918300
C	-1.78805600	-0.16612100	3.98136800
H	-2.14190700	-0.53118700	7.21241100
H	-2.74428700	0.86901200	5.60053400
H	-2.19561900	0.46426400	3.18594400
H	-0.79693800	0.20982100	4.24561100
H	-1.68770500	-1.18915600	3.62268900
N	-4.54429400	-0.88041100	3.70405500
C	-5.88614400	-1.37921000	3.44045900
C	-5.98321900	-2.86278000	3.08444300
O	-6.95326500	-3.26152900	2.43773000
H	-3.82928200	-1.16823300	3.04657200
H	-6.47496200	-1.20027800	4.34376200
H	-6.35159600	-0.82829700	2.62232800
N	-5.00913900	-3.67745300	3.54488300
C	-5.11369100	-5.10980900	3.31797000
H	-4.24712800	-3.32244700	4.11332700
H	-5.12192900	-5.32501800	2.24458500
C	-8.73313700	-5.47738900	-0.17445000
C	-7.33103400	-5.07798800	-0.65990800
C	-7.13631500	-3.56710500	-0.87628600
C	-5.64950000	-3.21612500	-1.00775500
C	-7.91687400	-3.04074400	-2.08693800
H	-9.50731300	-5.17910400	-0.88994600
H	-6.60293800	-5.41260300	0.08977600
H	-7.08590700	-5.61181000	-1.59006000
H	-7.50594600	-3.06473700	0.02290100
H	-5.51951900	-2.13886800	-1.15619800

H	-5.09460400	-3.49630700	-0.10641000
H	-5.19385100	-3.73082000	-1.86420700
H	-7.78997600	-1.95871500	-2.18981200
H	-7.56456600	-3.51362600	-3.01309900
H	-8.99006000	-3.23767000	-2.00015500
C	1.69264500	-6.04289100	3.02052800
O	1.00001200	-6.39147900	3.96891600
N	1.68762900	-4.75529200	2.53474800
C	1.05699800	-3.69376900	3.28555900
C	0.31517600	-2.69877600	2.39863600
S	-1.36873800	-3.27313900	1.92216900
O	5.00385300	-3.18820300	-3.37715800
C	-1.56395400	-0.15786500	-0.85720300
C	-0.95946800	-0.98684300	0.25428200
C	-1.95771200	-1.84899100	1.01324700
O	-3.15864700	-1.60582600	1.02440700
C	-1.30403000	1.14940400	-0.92289500
C	-2.37359700	-0.86781200	-1.90806000
H	-2.85523900	-0.14561700	-2.56756300
H	-1.74619900	-1.50967600	-2.53787100
H	-3.15359600	-1.49426900	-1.46830200
H	-0.69039700	1.65166700	-0.18622800
H	-0.16352300	-1.62184400	-0.14327500
H	-0.47410400	-0.33237100	0.98994600
H	-1.68837700	1.76652200	-1.72604000
H	0.89911400	-2.49457000	1.50572900
H	0.20187400	-1.75856500	2.94029900
H	0.39441900	-4.15800000	4.01962300
H	1.80691100	-3.11097500	3.83703700
H	2.42261200	-4.49606000	1.88155700
C	2.63079300	-6.97530700	2.28317600
H	2.50430700	-6.89430900	1.19832900
H	3.67463700	-6.71770100	2.49342000
H	2.44135000	-7.99888400	2.60412700
H	-0.62683400	-4.59719100	-6.49243700
H	1.12864300	-4.68896400	-6.03631000
H	5.91070400	-6.61985500	-3.14096700
H	8.15291200	-1.14710300	-4.67803300
H	9.71432700	-1.85511900	-4.06072400
H	8.18981300	0.66444000	-4.25267000
H	8.39800200	2.30848900	-4.99184600
H	0.75196700	2.25701600	7.44569000
H	2.52358600	2.34143000	7.55874400
H	6.54202000	6.84669300	-0.14188600
H	4.72570500	6.89617000	-0.04116300
H	2.96648000	9.43351000	-1.58329900
H	3.77178900	7.86918200	-1.30408900
H	2.96252300	8.70121000	3.17235100
H	4.05505500	8.00348700	4.43728100
H	-2.39214900	8.07832300	3.92795500
H	-2.71186800	7.12555900	2.45467200
H	-5.45013100	5.75016900	-4.72456000
H	-6.81537000	4.89994400	-3.91173200
H	-8.70458500	0.63508800	-5.70253500
H	-8.19028700	2.15575200	-6.52978400
H	-11.40745900	0.41514600	-4.03275200
H	-9.68962100	0.96754700	-4.17025300
H	-8.93778800	-4.96857800	0.77342100
H	-8.79304100	-6.56517700	-0.06700700
H	-4.26101000	-5.62684900	3.77040800

H	-6.05227100	-5.49136200	3.73341100
H	-3.21994100	-2.83867900	8.11725300
H	-1.46784300	-2.53226300	8.27772700
H	0.26419000	0.56833900	-6.55293200
H	0.43864100	2.24236900	-5.85220600
H	4.03351500	-3.07836700	-3.36675700
H	5.32520600	-2.28072500	-3.48324900
O	-3.85989400	1.10977900	1.36032800
H	-4.41318700	0.89144800	2.12261400
H	-3.62044100	0.25296000	0.96640900
O	3.93102800	1.07662600	-5.08851500
H	3.93058600	0.89565700	-6.03742500
H	3.02327700	0.83816700	-4.76402200
O	-2.98209400	3.46289900	0.17528000
H	-3.31274100	2.67046400	0.64305800
H	-3.36952400	3.39148800	-0.70267000

Intramolecular decarboxylation mechanism:
Protonated carboxylate group of MG-CoA
Deprotonated Glu72$_B$

E:S$_{p,dpGlu72}$ (0.0)

C	2.60380500	8.22734500	-2.06397800
C	1.76652300	7.51775000	-3.13944300
C	1.56672100	6.03929700	-2.90445900
C	0.52151500	5.57790200	-2.09591800
C	2.40709700	5.09694400	-3.51152600
C	0.29826700	4.21162300	-1.92785700
C	2.19452300	3.72925100	-3.34157200
C	1.12877500	3.28661600	-2.55862000
H	2.12465600	8.17516500	-1.08274400
H	0.78569600	8.00480900	-3.20522700
H	2.24611900	7.65892400	-4.11545600
H	-0.13533300	6.29756500	-1.61469500
H	3.22287700	5.44228800	-4.14192900
H	-0.54570100	3.87089600	-1.33758700
H	2.83453700	3.00159500	-3.82963500
H	0.95542500	2.22416000	-2.45429700
C	5.50887200	6.37642600	-0.41241100
C	5.40761600	4.98043900	-1.03047100
C	4.29257000	4.13927100	-0.40817700
S	4.59191200	3.60449800	1.32461500
C	6.11814200	2.61537600	1.13079900
H	5.58148800	6.29581100	0.67734500
H	5.18535000	5.06670000	-2.10261200
H	6.36420700	4.44952600	-0.96334500
H	3.36854400	4.71943600	-0.35552600
H	4.09859300	3.25017500	-1.00754500
H	6.29768100	2.13520700	2.09463500
H	6.02464300	1.83843700	0.36701700
H	6.97490400	3.25361300	0.90206700
C	-2.39405800	7.23120800	2.95797800
C	-0.93800900	6.82086400	3.11493100
O	-0.23086500	7.35468700	3.98128200
H	-3.00674800	6.57234700	3.58426400
N	-0.45178600	5.82678500	2.32630100
C	0.89616200	5.27930200	2.58805100
C	1.99137900	6.25724100	2.12243300
O	2.34279900	6.33284400	0.94644100
C	0.92911700	3.96313500	1.79857700
C	-0.54618200	3.60364200	1.60155600
C	-1.20843100	4.97090100	1.40184000
H	0.99506700	5.11712000	3.66721200
H	1.39402900	4.15489100	0.82893900
H	1.51448500	3.19648800	2.31003200
H	-0.69290500	2.95436100	0.73826400
H	-0.97016200	3.12164400	2.48904400
H	-1.06707500	5.31737300	0.37148500
H	-2.27459400	4.97399800	1.62362200
N	2.52470400	6.99661200	3.12804200
C	3.41636300	8.10954900	2.82622100
H	1.88025800	7.12338700	3.90408200
H	4.12703800	7.79850600	2.06088800
C	1.78066000	2.30285500	7.08618500
C	1.85459700	1.92440800	5.59503200
C	2.96347600	0.94970700	5.27173500
C	2.85831300	-0.39127100	5.66752400
C	4.12065100	1.35760500	4.59783800
C	3.88025200	-1.30191400	5.40115300
C	5.14613000	0.44872900	4.33083900
C	5.02962800	-0.88274500	4.72852500
H	1.82358300	1.41728900	7.72711500
H	0.89263600	1.49201400	5.29727800
H	1.97424300	2.83339900	4.99531800
H	1.96404100	-0.72601200	6.18918600
H	4.21203000	2.38821100	4.26571700
H	3.77658100	-2.33909000	5.70496600
H	6.03499000	0.77799700	3.80277700
H	5.82128500	-1.59122600	4.50920000
C	0.18088200	-5.21042400	-5.20420600
C	-0.10391800	-4.15026700	-4.15744400
O	-0.60152800	-3.05776700	-4.40838700
H	0.06934600	-6.19264700	-4.72517700
N	0.16891600	-4.55676800	-2.86899900
C	0.26882600	-3.55591400	-1.83788500
C	1.63881900	-2.88266800	-1.90048200
O	2.47297100	-3.28466400	-2.70239500
H	0.83245600	-5.31662900	-2.75493900
H	-0.52027200	-2.81835900	-1.98284400
H	0.14158700	-4.02005800	-0.85439100
N	1.86546800	-1.83466200	-1.06317800
C	3.24119700	-1.42951400	-0.78459000
C	3.99443500	-2.61457000	-0.13917400
O	3.36142900	-3.58080300	0.33787100
C	3.31427600	-0.13127600	0.05469000
C	2.53246900	0.99471200	-0.61681300
C	2.88886500	-0.27467600	1.51425200
H	1.17960600	-1.67714900	-0.33866000
H	3.72028400	-1.22839900	-1.74593000
H	4.37034200	0.14380300	0.04151600
H	2.59754800	1.90428500	-0.01751900
H	2.91042800	1.20945400	-1.61751700
H	1.47184200	0.73664500	-0.72286300
H	3.12984800	0.64603600	2.04997900
H	1.80829500	-0.42669700	1.61003700
H	3.40210600	-1.09537800	2.01962300

N	5.32202400	-2.52344700	-0.15854100
C	6.20900100	-3.51153200	0.46369400
C	5.86050500	-4.97380900	0.08954500
O	5.77052900	-5.84354600	0.95720300
C	6.26932800	-3.32644000	1.97909200
H	5.75563900	-1.66084200	-0.53632600
H	7.18717200	-3.31462600	0.01353700
H	6.96904000	-4.04005000	2.41790300
H	6.59512000	-2.30784500	2.20891500
H	5.29103900	-3.49750500	2.43379000
N	5.77284700	-5.21558100	-1.24124800
C	5.41659900	-6.52412500	-1.77783800
C	3.97429400	-6.49623700	-2.31895500
O	2.99364300	-6.15271500	-1.34844900
H	5.76514100	-4.43468000	-1.90310700
H	5.52618500	-7.25313100	-0.97363500
H	3.94015300	-5.79949200	-3.16876600
H	3.70734600	-7.49313900	-2.68962400
H	3.26690600	-5.31934000	-0.92639200
C	-0.17005300	0.72805400	-5.83776100
C	0.08197900	0.21967200	-4.41736200
S	1.90778100	0.01364200	-4.22269800
H	-1.24071400	0.87413800	-6.01199700
H	-0.28902100	0.93841200	-3.67919100
H	-0.38469200	-0.75545900	-4.26363900
H	1.86685000	-0.59262800	-3.02445400
C	8.76679900	-2.27607600	-4.40636400
C	8.09204800	-3.25584300	-3.45698100
O	7.81804700	-2.67829900	-2.18255600
H	8.95178800	-2.77311900	-5.36783200
H	7.17953400	-3.65150400	-3.91257400
H	8.75960900	-4.10504900	-3.26690600
H	7.21922700	-1.92766900	-2.35842100
C	7.65412800	1.37828300	-4.64739800
C	7.11417600	2.14207700	-3.42974700
C	5.81957000	1.55726400	-2.83418000
C	5.99464400	0.15027700	-2.27674300
O	6.24020400	0.00235000	-1.04861800
O	5.89509900	-0.81491800	-3.10202200
H	6.82811800	1.09137200	-5.30871800
H	7.87721400	2.19289300	-2.64287300
H	6.90042300	3.17961200	-3.71608400
H	5.47536400	2.20760700	-2.02917900
H	5.04767600	1.52536100	-3.61118900
C	-5.87083100	4.47251700	-4.20441700
C	-5.33262200	5.15023200	-2.93180800
C	-3.83746400	5.50885600	-3.08025300
C	-5.60524800	4.33728800	-1.66583900
C	-2.91190300	4.33777000	-3.42862200
H	-5.40575800	3.49688300	-4.37858900
H	-5.87370100	6.10262400	-2.82984000
H	-3.73856900	6.27955700	-3.85692000
H	-3.49463700	5.97097000	-2.14520000
H	-5.36345100	4.90743200	-0.76429000
H	-6.65670500	4.03979200	-1.59911500
H	-5.00069700	3.42754000	-1.64469500
H	-1.87511900	4.67517300	-3.49711700
H	-2.94855900	3.55449300	-2.66670500
H	-3.17398100	3.88827400	-4.39172800
C	-8.00344000	0.31682400	-6.19704700

C	-6.64393700	0.32321300	-5.48786000
C	-6.30061100	-0.99937100	-4.78262300
C	-6.33552400	-2.20292900	-5.73198900
C	-4.93766700	-0.90443700	-4.09384000
H	-7.97543400	-0.29275200	-7.10613000
H	-5.84910800	0.56789200	-6.20647900
H	-6.63101500	1.12290200	-4.73608500
H	-7.05897800	-1.16062200	-4.00247400
H	-6.07270100	-3.12270200	-5.19928200
H	-7.32512900	-2.34786200	-6.17502700
H	-5.61555700	-2.07417800	-6.54967400
H	-4.71234200	-1.82795900	-3.55433500
H	-4.13618600	-0.74056100	-4.82456800
H	-4.91599600	-0.07696200	-3.37731000
C	-10.46507100	-0.09367800	-3.31964400
C	-10.47833500	0.73791800	-2.02916600
C	-9.13156900	0.60581600	-1.34365200
C	-8.96347800	-0.22836600	-0.23096300
C	-7.99910400	1.23412300	-1.87936200
C	-7.70550500	-0.42586600	0.34326900
C	-6.73923600	1.03578900	-1.31683000
C	-6.59108800	0.21168200	-0.20145400
H	-10.09369400	-1.09618600	-3.06804900
H	-10.69241300	1.79002900	-2.25292800
H	-11.26911200	0.38652500	-1.35762800
H	-9.82815100	-0.73850100	0.18561900
H	-8.10297200	1.88286500	-2.74551400
H	-7.58790500	-1.09180800	1.19293800
H	-5.87697600	1.53523200	-1.74078600
H	-5.61223600	0.08289400	0.24794500
C	-2.00990200	-2.46646400	7.97674200
C	-2.11193800	-1.97920300	6.54112900
O	-2.40704300	-2.72665800	5.60686000
H	-1.79883900	-3.53479800	7.97674300
N	-1.91174100	-0.64629400	6.35639000
C	-2.00741800	-0.00151600	5.04557300
C	-3.42471600	-0.18108500	4.45427500
O	-4.35264000	0.50959000	4.87052300
C	-0.86536300	-0.42621800	4.12732800
H	-1.71973300	-0.06354800	7.15615800
H	-1.94769500	1.07474700	5.22079400
H	-0.91776500	0.12499000	3.18478300
H	0.09738300	-0.20883200	4.59099800
H	-0.90317200	-1.49821400	3.93708200
N	-3.55852300	-1.05681500	3.41688300
C	-4.86170100	-1.35415200	2.83504300
C	-5.38683300	-2.77464800	3.04729800
O	-6.35181900	-3.15003200	2.38389300
H	-2.85133100	-1.77724100	3.34496600
H	-5.57363600	-0.62624000	3.22653900
H	-4.81994000	-1.24222400	1.74945700
N	-4.71974100	-3.56171200	3.91694100
C	-4.88894000	-5.00232300	3.87484900
H	-3.95511700	-3.19511800	4.47266900
H	-4.90646600	-5.32534800	2.82935500
C	-8.54618800	-5.75222700	0.48407000
C	-7.23773900	-5.29979900	-0.16671000
C	-7.27359300	-3.84274700	-0.66249400
C	-5.86941200	-3.35816800	-1.02458700
C	-8.22381600	-3.63153900	-1.84798800

H	-9.38941300	-5.56563000	-0.19333500
H	-6.42292900	-5.39305100	0.56006500
H	-6.98826000	-5.96091100	-1.00956200
H	-7.62239400	-3.22766100	0.17367300
H	-5.90053700	-2.31568100	-1.35532400
H	-5.19752000	-3.42825600	-0.16619800
H	-5.44382300	-3.95613500	-1.84185300
H	-8.23894900	-2.57538600	-2.13800100
H	-7.89816400	-4.21446800	-2.71920200
H	-9.25339400	-3.92323400	-1.61726700
C	2.18949200	-5.59650800	3.53826900
O	1.36170400	-5.72034800	4.43935300
N	2.54712200	-4.38031000	3.02771200
C	1.79600500	-3.19989800	3.36789100
C	0.81165200	-2.79358000	2.27139400
S	-0.72363300	-3.80501300	2.28985200
O	5.05757500	-3.40748900	-3.42918300
C	-1.99114300	-0.97803500	-0.39329000
C	-1.46346000	-1.58127800	0.69512300
C	-1.84692100	-2.92199200	1.21043700
O	-2.94488400	-3.42179900	1.01102900
C	-1.68164300	0.48362900	-0.62669900
C	-2.69066500	1.41350400	0.04934600
O	-3.17486000	1.01170200	1.24864100
O	-3.01613100	2.48945100	-0.39387800
C	-2.92195800	-1.60541700	-1.37806200
H	-3.88021500	-1.07487400	-1.38407800
H	-2.50487700	-1.50496800	-2.38724800
H	-3.11766400	-2.65121900	-1.16183200
H	-0.70403700	0.75586000	-0.20922800
H	-0.72945500	-1.02898500	1.26866000
H	-2.88444900	0.10994100	1.48762000
H	-1.66956200	0.74115100	-1.68715900
H	1.30853400	-2.90652000	1.30752000
H	0.54531900	-1.75303800	2.43241600
H	1.28550200	-3.39409800	4.31387700
H	2.47561200	-2.35827400	3.51498400
H	3.09018800	-4.34453800	2.16752100
C	2.88652700	-6.75486900	2.87487900
H	2.32216300	-7.04406100	1.98037400
H	3.89768100	-6.50137500	2.54250200
H	2.90785100	-7.60554400	3.55732500
H	-0.55462100	-5.20520300	-6.01557800
H	1.20875300	-5.21462900	-5.58163200
H	6.07977100	-6.77529100	-2.61225800
H	8.15632400	-1.39722900	-4.63818800
H	9.74414800	-2.01167900	-3.98907500
H	8.15164200	0.44403900	-4.36671600
H	8.30634400	2.02471100	-5.24435700
H	0.83918700	2.82947800	7.27558500
H	2.60928100	2.96727300	7.35250100
H	6.40034400	6.90722100	-0.76215000
H	4.58499600	6.91979500	-0.63670500
H	2.73852100	9.27370700	-2.35711500
H	3.58830200	7.75901700	-1.96077500
H	2.82032600	8.94427700	2.44257300
H	3.94831800	8.38288100	3.74369900
H	-2.50516700	8.25410000	3.33407500
H	-2.82044200	7.17302500	1.95114100
H	-5.62190500	5.13058100	-5.04264200
H	-6.95303300	4.31827900	-4.13951400
H	-8.75483100	-0.12796300	-5.53621400
H	-8.29201400	1.33010800	-6.49598500
H	-11.42733200	-0.27392500	-3.81037200
H	-9.72662300	0.30763800	-4.02140700
H	-8.75053900	-5.17107200	1.38938100
H	-8.57585200	-6.82852900	0.68334300
H	-4.01671000	-5.45808400	4.35497600
H	-5.81128800	-5.37083000	4.33558400
H	-2.98824100	-2.28911600	8.43573500
H	-1.24264800	-1.92671000	8.54168400
H	0.20024900	-0.04255800	-6.52232600
H	0.34038400	1.68812100	-5.96741800
H	4.09960800	-3.33263000	-3.27340900
H	5.39735900	-2.52117000	-3.18786500
O	-3.27035800	2.79734000	3.57072700
H	-3.86566900	2.32342700	4.17680600
H	-3.26348600	2.22976600	2.78351800
O	5.71011300	-1.65077300	-5.77657300
H	5.72707100	-1.06326900	-5.00175200
H	5.41745600	-2.47707000	-5.36146200
O	-4.42709300	4.51215300	1.55386600
H	-4.19855100	4.05503600	2.38288600

E:$S_{p,dpGlu72}$-anti (+11.2)

C	2.56600600	8.26540000	-1.99512900
C	1.72697800	7.55300400	-3.06695500
C	1.52804800	6.07445600	-2.82790500
C	0.50702700	5.61466600	-1.98737900
C	2.34504600	5.12996500	-3.46260700
C	0.28332700	4.24855400	-1.81707300
C	2.13244400	3.76239300	-3.28996600
C	1.08936900	3.32132400	-2.47638300
H	2.09268300	8.20442200	-1.01140000
H	0.74552200	8.03961500	-3.13092000
H	2.20289600	7.69205100	-4.04495800
H	-0.12724600	6.33592700	-1.47857700
H	3.14334800	5.47364900	-4.11590100
H	-0.54177300	3.90689900	-1.20099500
H	2.75480900	3.03326300	-3.79799100
H	0.91462100	2.25925900	-2.36825800
C	5.48757500	6.42316700	-0.36323100
C	5.38980100	5.02944200	-0.98700500
C	4.27101400	4.18807800	-0.37049000
S	4.56469200	3.65059400	1.36223100
C	6.07934600	2.64434100	1.16532400
H	5.56310600	6.33657800	0.72607700
H	5.17223600	5.11880100	-2.05988700
H	6.34536000	4.49733800	-0.91642800
H	3.34665000	4.76851400	-0.32231400
H	4.07844400	3.29902200	-0.97087900
H	6.23825300	2.13702200	2.11861700
H	5.98353800	1.88765800	0.38169700
H	6.94794000	3.27511200	0.96171400
C	-2.41680000	7.18807900	3.02513000
C	-0.95459400	6.79596500	3.17693600
O	-0.24395800	7.36194000	4.01692000
H	-3.02328000	6.52249600	3.65147800
N	-0.47715900	5.78254100	2.40962000

C	0.88764400	5.26188000	2.63782400
C	1.95656900	6.27038400	2.17306600
O	2.27339800	6.38388700	0.99114700
C	0.92911300	3.96434000	1.82094800
C	-0.53887600	3.54690000	1.69998000
C	-1.26739800	4.88683600	1.55275500
H	1.01108300	5.07694600	3.71151100
H	1.32879500	4.19544000	0.83094800
H	1.57462500	3.21293200	2.27852800
H	-0.71601900	2.89660400	0.84518800
H	-0.88517500	3.03150300	2.60432700
H	-1.23834500	5.22903800	0.51161400
H	-2.30731200	4.85275300	1.87269100
N	2.51185500	6.98394600	3.18531500
C	3.38650200	8.11231600	2.89297900
H	1.89156800	7.08406200	3.98294900
H	4.10106400	7.81845300	2.12418800
C	1.80193400	2.25748900	7.10564200
C	1.88012900	1.88781800	5.61245400
C	3.00168900	0.92912700	5.28522500
C	2.89896200	-0.42151200	5.64677100
C	4.16772900	1.36144100	4.64273200
C	3.93114400	-1.31892800	5.37487100
C	5.20428300	0.46632000	4.37225600
C	5.08916000	-0.87562100	4.73353600
H	1.85205900	1.36751800	7.73974200
H	0.92482300	1.44268000	5.31257500
H	1.98957200	2.80144200	5.01721300
H	1.99757900	-0.77493500	6.14298100
H	4.25782800	2.40083900	4.33841800
H	3.82813200	-2.36429900	5.64912500
H	6.10101100	0.81479000	3.87023500
H	5.88854500	-1.57380000	4.50941000
C	0.24233700	-5.16354300	-5.24609100
C	-0.04919100	-4.09674700	-4.20879100
O	-0.52436100	-2.99900900	-4.47877000
H	0.13673400	-6.14554600	-4.76621900
N	0.19114300	-4.50093400	-2.91446100
C	0.27741500	-3.49828900	-1.88346500
C	1.66102400	-2.85183600	-1.90660800
O	2.51228900	-3.27018200	-2.68359400
H	0.84347300	-5.26790700	-2.78685100
H	-0.49293800	-2.74696700	-2.05542700
H	0.10824100	-3.95432100	-0.90283200
N	1.88321800	-1.81009600	-1.06290000
C	3.25557100	-1.40147000	-0.77902400
C	4.02264900	-2.59028200	-0.15735400
O	3.40569300	-3.56934400	0.31084300
C	3.32606500	-0.11837900	0.08315900
C	2.53042300	1.01455700	-0.56107700
C	2.91892500	-0.29101500	1.54520300
H	1.17480000	-1.62288700	-0.36697900
H	3.73276600	-1.17902000	-1.73712800
H	4.37994000	0.16430100	0.06233700
H	2.61048200	1.91784000	0.04631400
H	2.88994100	1.23875600	-1.56676300
H	1.46699500	0.76103600	-0.64694200
H	3.16262200	0.62061800	2.09560300
H	1.84149900	-0.45197100	1.65146700
H	3.44259100	-1.11858900	2.02836300

N	5.35026700	-2.48393000	-0.18544100
C	6.25043200	-3.46407600	0.42943400
C	5.93003600	-4.92800300	0.03825400
O	5.87017900	-5.81035200	0.89579800
C	6.29765000	-3.29610300	1.94759000
H	5.77128500	-1.61230700	-0.55321000
H	7.22774800	-3.24610100	-0.01290700
H	7.00785600	-4.00145500	2.38282500
H	6.60140800	-2.27398400	2.19181800
H	5.31905900	-3.49220300	2.39151200
N	5.83260600	-5.15613500	-1.29417300
C	5.49311200	-6.46571000	-1.83843900
C	4.04793500	-6.45539400	-2.37192400
O	3.06820800	-6.14239900	-1.39182000
H	5.80194300	-4.36871100	-1.94778200
H	5.61569600	-7.19763600	-1.03874600
H	3.99695000	-5.74894600	-3.21304600
H	3.79663500	-7.45214500	-2.75414800
H	3.31902300	-5.30024000	-0.97360600
C	-0.15543500	0.77723200	-5.82857000
C	0.08752200	0.26207300	-4.40925200
S	1.91255700	0.07422000	-4.18985700
H	-1.22644500	0.91775200	-6.00724100
H	-0.30077500	0.96975100	-3.66959200
H	-0.36947100	-0.71923600	-4.26822500
H	1.86051800	-0.53938500	-2.99517000
C	8.80646500	-2.16961300	-4.43541000
C	8.13580100	-3.15529700	-3.48827200
O	7.86595600	-2.58313300	-2.21090200
H	8.99193700	-2.66037500	-5.39994000
H	7.22182500	-3.54891800	-3.94305600
H	8.80420400	-4.00532700	-3.30462400
H	7.25401300	-1.84136600	-2.38100800
C	7.66518400	1.47793000	-4.64372500
C	7.11863100	2.22921600	-3.42074300
C	5.82493000	1.63535500	-2.83178200
C	6.00683900	0.22735900	-2.27770200
O	6.23736100	0.07680400	-1.04746100
O	5.93051300	-0.73381000	-3.11008100
H	6.84122400	1.18957800	-5.30698300
H	7.87984400	2.27742100	-2.63183400
H	6.90171600	3.26816300	-3.70004900
H	5.47356700	2.28160800	-2.02627600
H	5.05597700	1.60124400	-3.61165300
C	-5.88305000	4.46364800	-4.15555500
C	-5.34453400	5.17425600	-2.90262700
C	-3.84143400	5.49778900	-3.04813600
C	-5.64144100	4.43080200	-1.60069500
C	-2.92478100	4.29115200	-3.27476600
H	-5.40517400	3.49439800	-4.32464500
H	-5.86862300	6.13987200	-2.84568800
H	-3.71874600	6.20077300	-3.88295700
H	-3.50970500	6.03553300	-2.14931200
H	-5.36713600	5.07481400	-0.75724100
H	-6.70084600	4.17110000	-1.50905600
H	-5.05995800	3.51081100	-1.51430200
H	-1.88621200	4.61518000	-3.37112200
H	-2.96703300	3.58535600	-2.44030100
H	-3.18407000	3.75126000	-4.19096900
C	-7.98594400	0.30838000	-6.18025900

C	-6.62415400	0.31260400	-5.47700100
C	-6.27311800	-1.01723600	-4.78954800
C	-6.31275200	-2.21015000	-5.75207700
C	-4.90578300	-0.92647100	-4.11042100
H	-7.95796300	-0.29233600	-7.09530600
H	-5.83325000	0.56930600	-6.19586200
H	-6.61152100	1.10236200	-4.71455100
H	-7.02571100	-1.18855600	-4.00595800
H	-6.04009300	-3.13398500	-5.23171100
H	-7.30602000	-2.35545600	-6.18692300
H	-5.60130800	-2.06943100	-6.57529800
H	-4.67654100	-1.85031300	-3.57379400
H	-4.10921800	-0.76126000	-4.84627000
H	-4.87737400	-0.10201000	-3.39113800
C	-10.44002900	-0.14575200	-3.30298600
C	-10.46005000	0.69436900	-2.01713800
C	-9.10793800	0.58553500	-1.33807700
C	-8.92361400	-0.24865200	-0.22778800
C	-7.98519200	1.22477000	-1.88183000
C	-7.65889500	-0.44154500	0.33146200
C	-6.71747600	1.02893000	-1.33387100
C	-6.55196800	0.19890900	-0.22492500
H	-10.05941900	-1.14368400	-3.04942700
H	-10.69133800	1.74128300	-2.24934900
H	-11.24247000	0.33691900	-1.33897200
H	-9.78116200	-0.76621600	0.19453000
H	-8.10210200	1.87048900	-2.74905400
H	-7.52635400	-1.10840700	1.17807600
H	-5.85809200	1.52286600	-1.77301500
H	-5.56923200	0.05774000	0.20715400
C	-1.95014200	-2.54891000	7.96083900
C	-2.02311700	-2.06242800	6.52420800
O	-2.25812500	-2.81654300	5.58088800
H	-1.73066100	-3.61578400	7.96172400
N	-1.84265300	-0.72086600	6.36602600
C	-1.91622400	-0.02320400	5.07896200
C	-3.33767300	-0.10184100	4.48056400
O	-4.17277000	0.75899700	4.79712400
C	-0.81187700	-0.46813500	4.12448100
H	-1.72675300	-0.15506000	7.19214000
H	-1.79481900	1.03689600	5.31111600
H	-0.89249700	0.08237400	3.18356300
H	0.17088900	-0.27843300	4.55779400
H	-0.88228800	-1.53757300	3.92952300
N	-3.57405500	-1.11095100	3.62840800
C	-4.83959300	-1.37365400	2.96886700
C	-5.34037400	-2.81273700	3.09368400
O	-6.30281100	-3.16867900	2.41536400
H	-2.82095300	-1.76158000	3.45437100
H	-5.58830200	-0.68422900	3.35913400
H	-4.73902300	-1.19356600	1.89605800
N	-4.65699700	-3.63143100	3.92184900
C	-4.81565200	-5.07214500	3.84183300
H	-3.88522800	-3.27620300	4.47476100
H	-4.83075900	-5.37357800	2.79000700
C	-8.47188100	-5.82137600	0.44955900
C	-7.16840800	-5.34781600	-0.19668900
C	-7.21273100	-3.87980800	-0.65927600
C	-5.81495700	-3.38739000	-1.03585100
C	-8.18386300	-3.64322800	-1.82320500

H	-9.31745400	-5.63558400	-0.22515800
H	-6.34913800	-5.45299200	0.52358300
H	-6.91908600	-5.98867200	-1.05512500
H	-7.54716900	-3.28424000	0.19698800
H	-5.85212500	-2.34026200	-1.35056600
H	-5.12565600	-3.46733300	-0.19275700
H	-5.40271300	-3.97196000	-1.86953200
H	-8.19870700	-2.58172400	-2.09305400
H	-7.87607600	-4.21095300	-2.71087600
H	-9.21088300	-3.93444300	-1.58013300
C	2.27864300	-5.61835600	3.52181600
O	1.51521100	-5.76893300	4.47388800
N	2.56140100	-4.39404500	2.98595100
C	1.78949700	-3.23311100	3.35002100
C	0.82004600	-2.81096200	2.24660800
S	-0.71232100	-3.82257300	2.22639300
O	5.08837500	-3.32603500	-3.45623000
C	-1.97461200	-0.93682200	-0.40823200
C	-1.43231700	-1.59077700	0.63859700
C	-1.80872000	-2.94655200	1.09521300
O	-2.87250600	-3.48850800	0.83462000
C	-1.72570800	0.54697100	-0.54971400
C	-2.79729500	1.37084000	0.17113700
O	-3.31812300	0.75383700	1.19858600
O	-3.07665400	2.51826300	-0.18331100
C	-2.88930600	-1.53168800	-1.42741000
H	-3.86938900	-1.04574300	-1.37271800
H	-2.49782600	-1.33948200	-2.43318900
H	-3.03936500	-2.59803600	-1.28613600
H	-0.76836300	0.83344600	-0.09760600
H	-0.74890800	-1.03989200	1.27074000
H	-4.08663900	1.26837800	1.70860400
H	-1.70129800	0.87080100	-1.59297500
H	1.32937900	-2.90572100	1.28724600
H	0.55028600	-1.77271300	2.41464500
H	1.26789700	-3.46496900	4.28171600
H	2.45461600	-2.38632000	3.53446200
H	3.07945800	-4.34206700	2.11161600
C	2.97177300	-6.75562400	2.81570700
H	2.37304200	-7.05329100	1.94689100
H	3.96054900	-6.47545500	2.43913900
H	3.04788800	-7.60938600	3.49037900
H	-0.49437800	-5.15709700	-6.05637500
H	1.26964700	-5.15654400	-5.62501000
H	6.15697800	-6.70461800	-2.67590100
H	8.18885700	-1.29359100	-4.65887100
H	9.78234500	-1.90121300	-4.01725700
H	8.17033900	0.54521400	-4.37173300
H	8.31150500	2.13444800	-5.23608800
H	0.85686500	2.77458200	7.30071800
H	2.62601600	2.92547500	7.37634300
H	6.37439800	6.96371000	-0.70981000
H	4.55916200	6.96107700	-0.58178100
H	2.69193500	9.31526000	-2.27930400
H	3.55404900	7.80392600	-1.89694600
H	2.78344800	8.94565500	2.51721000
H	3.91769000	8.38196100	3.81191100
H	-2.53518800	8.20661000	3.41044000
H	-2.84411800	7.13484900	2.01881700
H	-5.63981300	5.13035000	-4.98823100

H	-6.96335000	4.30004300	-4.09008700
H	-8.73291100	-0.14784400	-5.52223200
H	-8.28281600	1.32189100	-6.47019400
H	-11.40166800	-0.32911800	-3.79391400
H	-9.70589200	0.26737600	-4.00240200
H	-8.67938200	-5.24947200	1.36022100
H	-8.49290800	-6.89942400	0.63981600
H	-3.93904600	-5.52495200	4.31641300
H	-5.73426600	-5.45140600	4.30024200
H	-2.92915000	-2.38296400	8.42267000
H	-1.18629000	-2.00798700	8.52923200
H	0.21975300	0.01536300	-6.52017800
H	0.34730600	1.74229100	-5.95074900
H	4.13224800	-3.26838700	-3.27980500
H	5.42229000	-2.44025900	-3.20572700
O	-5.15536900	1.80081700	2.51390500
H	-4.93736700	1.55454400	3.44166600
H	-5.04120900	2.77331100	2.40199500
O	5.74357300	-1.55589400	-5.78898100
H	5.76248600	-0.97406700	-5.00986600
H	5.44293000	-2.38250600	-5.37989000
O	-4.49298800	4.19119100	1.53881600
H	-5.26968900	4.51565700	1.06610700
H	-4.00192500	3.66776000	0.86310200

$TS_{pc,dpGlu72}$ (+32.3)

C	2.68948400	8.29055100	-1.66584200
C	1.86156600	7.63142600	-2.78507100
C	1.72174800	6.12902400	-2.67029900
C	0.73794100	5.56489300	-1.84913400
C	2.54912700	5.26761500	-3.40426100
C	0.57195600	4.18228400	-1.78086000
C	2.39597500	3.88037000	-3.33963100
C	1.39813100	3.34026600	-2.52630700
H	2.21056500	8.18677600	-0.68906300
H	0.86166000	8.08269300	-2.80562500
H	2.32439300	7.86660800	-3.75094700
H	0.09021500	6.21762800	-1.27088700
H	3.31273500	5.69147500	-4.05191900
H	-0.22021800	3.76680400	-1.16648600
H	3.03130800	3.22569000	-3.93212800
H	1.27209100	2.26628400	-2.47038200
C	5.58390000	6.31829000	-0.14047400
C	5.47755100	4.95643200	-0.82932400
C	4.35532200	4.08775000	-0.26330400
S	4.62734300	3.47150000	1.44888000
C	6.09018100	2.40165400	1.21497000
H	5.66540400	6.18295900	0.94321200
H	5.26472900	5.09625400	-1.89736500
H	6.43291200	4.42051100	-0.78219600
H	3.43269200	4.66754200	-0.19162900
H	4.16865700	3.23053400	-0.90932900
H	6.28900900	1.93041700	2.17881200
H	5.92001700	1.61808400	0.47421400
H	6.96880800	2.98259200	0.92639300
C	-2.27509100	7.10270200	3.34754900
C	-0.82289700	6.66256300	3.45477500
O	-0.10078000	7.11626700	4.35452200
H	-2.88660000	6.41950700	3.94840400
N	-0.35851300	5.73134600	2.58133900
C	0.97901700	5.13571600	2.79536200
C	2.09530300	6.11892800	2.39257300
O	2.45350400	6.25650900	1.22401600
C	0.97439600	3.87240100	1.92041900
C	-0.51209700	3.56244500	1.71740200
C	-1.13706900	4.95564500	1.60319500
H	1.07868700	4.89978700	3.86072500
H	1.44076700	4.11364200	0.96264300
H	1.54474400	3.06198900	2.37894700
H	-0.69798700	2.95957600	0.82656600
H	-0.94161400	3.04714300	2.58161500
H	-0.98562900	5.36589000	0.59685600
H	-2.20440200	4.96121100	1.82095000
N	2.63927900	6.79064400	3.44139600
C	3.54543300	7.90945200	3.20366700
H	1.99427400	6.88072500	4.22271600
H	4.24723500	7.63432500	2.41644100
C	1.86931500	1.91231800	7.17435400
C	1.92059500	1.60015000	5.66662800
C	3.02951400	0.64639000	5.28683100
C	2.90678200	-0.72168700	5.56789100
C	4.20890600	1.09983300	4.68367400
C	3.93317000	-1.61441100	5.25931700
C	5.23923600	0.20927100	4.37692300
C	5.10498300	-1.14986300	4.65949500
H	1.90617800	0.99606500	7.77121300
H	0.95687400	1.17304000	5.36841400
H	2.02568500	2.53463600	5.10454000
H	1.99422900	-1.09027400	6.03182500
H	4.31469300	2.15391700	4.44138000
H	3.81628200	-2.67218900	5.47491000
H	6.14808800	0.57509200	3.91011000
H	5.90052600	-1.84343200	4.40770700
C	0.05288300	-4.93397900	-5.47088600
C	-0.18151500	-3.89071500	-4.39540300
O	-0.59890600	-2.75916600	-4.63286100
H	-0.07083600	-5.92918400	-5.02494400
N	0.05282000	-4.33537700	-3.11452100
C	0.23525700	-3.36203700	-2.06577800
C	1.67035200	-2.83374400	-2.08615200
O	2.40393400	-3.15290500	-3.02047700
H	0.65406300	-5.14736800	-3.01525600
H	-0.43641000	-2.52566500	-2.26028700
H	-0.00306600	-3.80427300	-1.09302700
N	2.02674300	-1.99938000	-1.09256700
C	3.41148100	-1.61174700	-0.88041600
C	4.15986900	-2.79884300	-0.25258900
O	3.52319400	-3.75262300	0.24767800
C	3.54443500	-0.33191400	-0.02174900
C	2.79342100	0.83605800	-0.65698000
C	3.13023400	-0.50587700	1.44067200
H	1.38919300	-1.88929700	-0.32136900
H	3.84637600	-1.40503300	-1.85989900
H	4.61348800	-0.10383400	-0.04034300
H	2.87986100	1.72313200	-0.02713100
H	3.18052300	1.06450700	-1.65111100
H	1.72941700	0.60130000	-0.77532500
H	3.39007700	0.39158900	2.00602700
H	2.04600400	-0.63521800	1.53921800

H	3.62410100	-1.35553400	1.91759200
N	5.48588300	-2.71593300	-0.27995800
C	6.35455400	-3.75247200	0.28018400
C	5.95499100	-5.16853200	-0.19782200
O	5.92306300	-6.12084500	0.58255100
C	6.41771700	-3.67299500	1.80392900
H	5.91550900	-1.93757500	-0.80513700
H	7.33808700	-3.55372700	-0.15787400
H	7.08272700	-4.44708900	2.19043500
H	6.78599500	-2.68839800	2.10651300
H	5.42996900	-3.83235600	2.24235800
N	5.73642800	-5.25902800	-1.53089600
C	5.30170900	-6.49197700	-2.16896200
C	3.85947000	-6.34100300	-2.68413100
O	2.90742900	-6.05094900	-1.66692100
H	5.70621000	-4.40693800	-2.10570000
H	5.37701300	-7.29180100	-1.43022100
H	3.84727500	-5.56242900	-3.46032400
H	3.54048800	-7.28239600	-3.14774700
H	3.24581600	-5.30447100	-1.14006100
C	-0.22177800	1.03398300	-5.79381800
C	-0.20901300	0.49200200	-4.37771400
S	1.44437500	-0.06546000	-3.82168100
H	-1.26570500	1.24695700	-6.06976200
H	-0.51341700	1.27195400	-3.67439300
H	-0.88377400	-0.36044300	-4.27822000
H	1.38270200	-1.28505100	-4.38558200
C	8.68560300	-2.15840500	-4.60815600
C	8.04727600	-3.15592100	-3.64354800
O	8.04119900	-2.68954500	-2.29708600
H	8.84245100	-2.63282600	-5.58595500
H	7.04636100	-3.43807400	-3.97732300
H	8.65681400	-4.06840600	-3.62966700
H	7.53058500	-1.85773300	-2.23316900
C	7.62116200	1.51813100	-4.64909100
C	7.25316800	2.20517400	-3.31964300
C	5.98201400	1.65817000	-2.64154800
C	5.96258200	0.13520700	-2.55211900
O	6.53819900	-0.41185500	-1.56125200
O	5.38119500	-0.49506700	-3.48850100
H	6.73240400	1.32700600	-5.25576900
H	8.09685800	2.11233300	-2.62461700
H	7.09974800	3.27922000	-3.48062000
H	5.91630000	2.07108400	-1.63426000
H	5.11241100	1.98106900	-3.21644100
C	-5.85528700	4.76339300	-3.91260800
C	-5.30416200	5.36210200	-2.60541100
C	-3.80109700	5.69882200	-2.72160100
C	-5.60139300	4.48903000	-1.38623400
C	-2.87987700	4.51999400	-3.05812100
H	-5.40338600	3.79251000	-4.14092600
H	-5.82486300	6.32000000	-2.45665500
H	-3.67495900	6.47698900	-3.48708500
H	-3.47404200	6.14594700	-1.77376400
H	-5.33217200	4.99133400	-0.45319500
H	-6.66319400	4.22735200	-1.33017100
H	-5.03255300	3.55720000	-1.42213100
H	-1.83794400	4.84882800	-3.10323500
H	-2.94079600	3.72966800	-2.30524100
H	-3.12512400	4.08130800	-4.03132400

C	-8.06348200	0.74588100	-6.09580100
C	-6.69981400	0.69022500	-5.39621000
C	-6.38280100	-0.67833800	-4.77104800
C	-6.42951400	-1.82036300	-5.79311300
C	-5.02501000	-0.65382800	-4.06554900
H	-8.05203200	0.18444900	-7.03581800
H	-5.90326500	0.96005100	-6.10385100
H	-6.66887400	1.44465600	-4.59990200
H	-7.14969600	-0.87466600	-4.00762000
H	-6.18763400	-2.77624500	-5.31668500
H	-7.41728600	-1.92089500	-6.25169300
H	-5.70086300	-1.65421300	-6.59639800
H	-4.81925600	-1.62519900	-3.60868100
H	-4.21513100	-0.43782000	-4.77369100
H	-5.00163700	0.10725300	-3.27879600
C	-10.50376200	0.21968000	-3.21914000
C	-10.48383800	0.97913400	-1.88447300
C	-9.13117800	0.78730600	-1.22283400
C	-8.96887300	-0.10268100	-0.15305600
C	-7.99131300	1.42282900	-1.73450500
C	-7.71051900	-0.34936800	0.40158200
C	-6.73076600	1.17601300	-1.19158100
C	-6.58854400	0.29490600	-0.11981300
H	-10.14463600	-0.80057100	-3.02718700
H	-10.68180900	2.04549700	-2.04731600
H	-11.27208700	0.60544800	-1.22205000
H	-9.83942100	-0.61681800	0.24624200
H	-8.08975600	2.11856800	-2.56406300
H	-7.59914000	-1.05650400	1.21826300
H	-5.86147000	1.68357700	-1.59026400
H	-5.60682500	0.12801500	0.31304300
C	-1.97797200	-2.84604100	7.85527900
C	-2.05707500	-2.28703400	6.44629300
O	-2.34798700	-2.98403100	5.47281200
H	-1.78198300	-3.91627800	7.80455300
N	-1.81933600	-0.95070000	6.34207400
C	-1.98703300	-0.19966200	5.09458600
C	-3.45047700	-0.30244600	4.61164400
O	-4.31791500	0.41261500	5.11990500
C	-0.93096400	-0.57429000	4.05794800
H	-1.69215100	-0.42024200	7.18996700
H	-1.87670900	0.85602900	5.34733100
H	-1.06038500	0.04076300	3.16465000
H	0.06894700	-0.39967900	4.45848200
H	-0.99933900	-1.63179300	3.80349600
N	-3.69727200	-1.16650700	3.60583800
C	-5.01485900	-1.37823600	3.04345500
C	-5.46896200	-2.83724900	3.03424300
O	-6.41820800	-3.17203500	2.32567100
H	-2.96893900	-1.81632200	3.35257400
H	-5.71941600	-0.76546900	3.60969200
H	-5.04168000	-1.04971500	2.00186500
N	-4.76498300	-3.69468800	3.80411700
C	-4.92931100	-5.12798900	3.65675700
H	-4.00576100	-3.36218100	4.38819900
H	-4.95906700	-5.37946300	2.59241400
C	-8.62860900	-5.65297900	0.26804600
C	-7.31784400	-5.18114400	-0.36427300
C	-7.34205500	-3.69754900	-0.77465300
C	-5.93281000	-3.20020500	-1.09813500

C	-8.28286800	-3.41141100	-1.95167300
H	-9.47364200	-5.41969000	-0.39217500
H	-6.50146600	-5.32443500	0.35265200
H	-7.07952700	-5.79572000	-1.24488700
H	-7.69175100	-3.12979100	0.09370300
H	-5.96618500	-2.14514500	-1.38920700
H	-5.27497800	-3.30054200	-0.23203700
H	-5.49674100	-3.76612800	-1.93295700
H	-8.29908500	-2.33839500	-2.17090400
H	-7.94870900	-3.93523700	-2.85665000
H	-9.31317800	-3.72019200	-1.74884200
C	2.05517100	-5.81577700	3.17215600
O	1.12706600	-6.00545000	3.95645600
N	2.48054600	-4.56287800	2.82646700
C	1.71657900	-3.40322900	3.21316700
C	0.76676100	-2.92114700	2.11116200
S	-0.80345700	-3.87975800	2.05682100
O	5.06564300	-3.21075800	-3.39619800
C	-1.97225400	-1.04415700	-0.67467200
C	-1.53305800	-1.50011800	0.61291000
C	-1.92836300	-2.86143100	1.12743800
O	-3.07115200	-3.26474400	0.97550500
C	-1.73643600	0.35936300	-0.92945700
C	-2.67878600	1.22957100	0.13493600
O	-3.10114000	2.27446500	-0.33805400
O	-2.78491300	0.73079000	1.29123200
C	-2.87623600	-1.79727000	-1.56842400
H	-3.89149600	-1.39487800	-1.45403700
H	-2.59099200	-1.62588500	-2.61084200
H	-2.92317300	-2.85840900	-1.34087000
H	-0.73251600	0.69946500	-0.65719700
H	-0.54369200	-1.12979100	0.87673300
H	-2.00538500	0.69625400	-1.92620600
H	1.28839800	-3.02629500	1.16004600
H	0.52638900	-1.87736700	2.30265300
H	1.17178700	-3.64719600	4.12768300
H	2.39633600	-2.57603800	3.42619800
H	3.09046400	-4.45736800	2.01715900
C	2.81177800	-6.92952100	2.49060400
H	2.32627800	-7.15414900	1.53340200
H	3.84759200	-6.66191800	2.26245700
H	2.77459500	-7.82480800	3.11241400
H	-0.68967400	-4.87627900	-6.27352400
H	1.07721300	-4.93158900	-5.85816700
H	5.95347500	-6.70826800	-3.02188200
H	8.08503500	-1.26062900	-4.78806200
H	9.67009400	-1.92883600	-4.18726500
H	8.10805900	0.56390300	-4.42216800
H	8.27647900	2.18574700	-5.21835400
H	0.93712300	2.44073900	7.39996300
H	2.70960200	2.55088200	7.46629000
H	6.47920100	6.85475500	-0.47173800
H	4.66561700	6.88497900	-0.32764100
H	2.83571500	9.34890500	-1.90575400
H	3.66823100	7.80454600	-1.59663900
H	2.95736500	8.77052200	2.86938200
H	4.08952900	8.12764400	4.12851100
H	-2.36868300	8.10573500	3.77705700
H	-2.71146300	7.10190400	2.34330300
H	-5.60547800	5.46169100	-4.71830800
H	-6.93902300	4.62150900	-3.84526600
H	-8.81458200	0.27724700	-5.45137500
H	-8.34062100	1.77686900	-6.33927800
H	-11.47280200	0.07773400	-3.70891600
H	-9.76625100	0.64683900	-3.90655000
H	-8.81540200	-5.11687700	1.20438900
H	-8.66992900	-6.73766300	0.41230700
H	-4.05907600	-5.61951800	4.10407400
H	-5.85244800	-5.50760300	4.10695600
H	-2.94948600	-2.67980800	8.33242500
H	-1.19812800	-2.34658600	8.43959300
H	0.13105300	0.29494600	-6.52070100
H	0.30029600	1.99266900	-5.87865000
H	4.09449400	-3.19379400	-3.35510500
H	5.30784300	-2.25690700	-3.45175800
O	-4.27335300	4.27332300	1.65652500
H	-4.01059400	3.76461400	2.44609500
H	-4.00801100	3.69267100	0.92541900
O	4.13194500	1.30652000	-5.24975900
H	4.58825000	0.62534400	-4.70812300
H	3.20641100	1.02982700	-5.18511200
O	-3.11995300	2.47789600	3.54275100
H	-3.74905800	2.06221800	4.15670900
H	-3.08683700	1.86675200	2.78379200

Int$_{p,dpGlu72}$ (-6.5)

C	2.95557200	8.21580500	-1.74193900
C	2.11116100	7.55502300	-2.84685600
C	1.97175300	6.05271300	-2.72257800
C	1.05411300	5.48449500	-1.82928000
C	2.74832200	5.19595500	-3.51456000
C	0.90218000	4.10040800	-1.75264300
C	2.60865100	3.80786200	-3.44144800
C	1.67392500	3.26303700	-2.56055100
H	2.46830000	8.14854600	-0.76559400
H	1.11290000	8.01114500	-2.85619400
H	2.56080900	7.78265700	-3.82048100
H	0.46417700	6.13318600	-1.18778000
H	3.46541900	5.62538000	-4.20996100
H	0.18269600	3.67048800	-1.06197500
H	3.21224800	3.15469600	-4.06784600
H	1.56460600	2.18892200	-2.49282400
C	5.76878000	6.19057000	-0.13591000
C	5.63062400	4.81993300	-0.79661200
C	4.47119700	4.01090800	-0.22124200
S	4.71175600	3.44114000	1.51120600
C	6.06535400	2.23164100	1.29585600
H	5.83065700	6.07379400	0.95133900
H	5.43857900	4.93928200	-1.87100500
H	6.56535900	4.25253000	-0.71845600
H	3.56803900	4.62325600	-0.17884900
H	4.26050400	3.14175300	-0.84144500
H	6.25874700	1.79792700	2.27926500
H	5.79880500	1.43071100	0.60352500
H	6.98022400	2.71500100	0.94598800
C	-2.12183100	7.25076900	3.20610500
C	-0.68375800	6.77872900	3.36087700
O	0.04337600	7.27736200	4.22995600
H	-2.76886700	6.59999600	3.80639500

N	-0.24250700	5.77055800	2.56342000
C	1.08528400	5.16561800	2.80898500
C	2.21779200	6.11952700	2.37369300
O	2.56490900	6.22276500	1.19916900
C	1.05844700	3.86709200	1.98663600
C	-0.43518900	3.54938500	1.83922600
C	-1.05762800	4.93820500	1.66837800
H	1.17773500	4.96744700	3.88321700
H	1.49850000	4.07018100	1.00774300
H	1.64283600	3.07512000	2.45816200
H	-0.65663800	2.88601300	0.99901200
H	-0.83295700	3.08213500	2.74889600
H	-0.95018000	5.28051400	0.62944700
H	-2.11396200	4.96790900	1.93194300
N	2.78448600	6.80292000	3.40242000
C	3.72077500	7.89394500	3.14655000
H	2.15122600	6.92388400	4.18751900
H	4.42615800	7.58252800	2.37615100
C	1.81383500	2.01427300	7.18707100
C	1.89470700	1.69471800	5.68059600
C	2.84800900	0.57468900	5.32700900
C	2.54096700	-0.74976100	5.67408900
C	4.04008800	0.82064200	4.63665400
C	3.38460900	-1.80319000	5.32391300
C	4.89675600	-0.22886600	4.29763500
C	4.57009600	-1.54227100	4.63323500
H	1.81071500	1.10422700	7.79372500
H	0.89284900	1.43296200	5.32351900
H	2.17684000	2.60086900	5.13330000
H	1.62668400	-0.96047000	6.22450000
H	4.28711700	1.83760200	4.34340900
H	3.11275700	-2.82401800	5.57639300
H	5.81169800	-0.02340400	3.75063400
H	5.22514700	-2.35777200	4.34831700
C	0.01394300	-4.99402100	-5.37129200
C	-0.17821500	-3.82528000	-4.42915900
O	-0.32034900	-2.67426800	-4.83692000
H	-0.16014100	-5.94420600	-4.85538900
N	-0.22479800	-4.15515800	-3.09928500
C	-0.02233000	-3.11485900	-2.11755000
C	1.47836600	-2.87584000	-1.92775100
O	2.28210800	-3.52755900	-2.59613000
H	0.21281000	-5.03630100	-2.85445000
H	-0.49553600	-2.20077800	-2.47736200
H	-0.48965300	-3.39635600	-1.16927800
N	1.83832700	-1.96810500	-1.00356500
C	3.24047300	-1.67330800	-0.75459100
C	3.92928800	-2.90275800	-0.14050500
O	3.28703400	-3.84402600	0.36096700
C	3.41365000	-0.44099700	0.16034200
C	2.96088400	0.83395400	-0.54441900
C	2.73015700	-0.59019200	1.52084800
H	1.12976600	-1.32771400	-0.67263300
H	3.73404600	-1.45690400	-1.70695700
H	4.49133400	-0.36786800	0.32635700
H	3.06390800	1.68807400	0.12734400
H	3.54397300	1.01605900	-1.44805200
H	1.91758600	0.77257800	-0.85152000
H	2.98946200	0.25304100	2.16352400
H	1.64068900	-0.59730500	1.41915400
H	3.02927400	-1.50702400	2.03321800
N	5.26223700	-2.83683300	-0.17283400
C	6.12403200	-3.84262500	0.44311400
C	5.77629800	-5.27147300	-0.02136100
O	5.76730000	-6.21521200	0.77024600
C	6.10720800	-3.73817600	1.96620100
H	5.70480600	-2.06725500	-0.69811900
H	7.12383500	-3.63285800	0.04863100
H	6.74509500	-4.50879500	2.40177800
H	6.46178400	-2.74946200	2.27222200
H	5.09077900	-3.88822500	2.33771800
N	5.57558800	-5.38561100	-1.35516700
C	5.16258600	-6.63982300	-1.95611400
C	3.71016800	-6.55110000	-2.45153300
O	2.77550500	-6.29337400	-1.41510700
H	5.53478200	-4.54587600	-1.94501500
H	5.25529700	-7.41076000	-1.18845600
H	3.64808800	-5.78336600	-3.23667000
H	3.42397000	-7.51150500	-2.89817400
H	3.02755400	-5.45308200	-0.99419000
C	-0.08927300	0.97262700	-5.79757600
C	-0.04605500	0.44359000	-4.37391200
S	1.63107700	-0.09670600	-3.85272000
H	-1.13282500	1.19711200	-6.06148100
H	-0.34765200	1.22523400	-3.67116700
H	-0.70611800	-0.41679100	-4.25570300
H	1.52531200	-1.34216500	-4.35447600
C	8.70548100	-2.44346800	-4.40965100
C	7.99180900	-3.36602600	-3.41819500
O	7.98700300	-2.83999800	-2.09435600
H	8.85434900	-2.95506900	-5.36922300
H	6.98117600	-3.60208300	-3.76137400
H	8.54331800	-4.31245300	-3.35224000
H	7.44070400	-2.02999300	-2.05828100
C	7.74490600	1.25981800	-4.52943800
C	7.36737100	1.97355200	-3.21656100
C	6.04288000	1.50705700	-2.58062300
C	5.92058200	-0.01214800	-2.46493200
O	6.41174600	-0.56879800	-1.43580300
O	5.34386900	-0.62392500	-3.41650900
H	6.86453000	1.08276700	-5.15200900
H	8.17883600	1.83864200	-2.49065200
H	7.28070900	3.05380700	-3.38686500
H	5.96691100	1.93926400	-1.58180200
H	5.21531800	1.87399700	-3.19049600
C	-5.64547300	4.88724300	-4.07357800
C	-5.13608800	5.34241700	-2.69364600
C	-3.61684900	5.12269400	-2.51420300
C	-5.95753600	4.66991800	-1.58527000
C	-3.16225000	3.66278000	-2.37295500
H	-5.25240200	3.89511100	-4.32565100
H	-5.30654400	6.42550700	-2.61822200
H	-3.09022900	5.57699100	-3.36308800
H	-3.28643200	5.68394500	-1.62845900
H	-5.61854700	4.94830600	-0.58405900
H	-7.01534100	4.94045000	-1.67222200
H	-5.89973900	3.57965400	-1.65671900
H	-2.06989000	3.60572700	-2.33397700
H	-3.55045300	3.19362300	-1.46169500
H	-3.49105900	3.04891300	-3.21821100

C	-7.92949700	0.89649000	-6.22671300
C	-6.56486100	0.84468200	-5.52626200
C	-6.24208400	-0.48763200	-4.82912400
C	-6.37723500	-1.69515800	-5.76392000
C	-4.83716900	-0.44027900	-4.22111800
H	-7.92068700	0.31590100	-7.15503800
H	-5.77253600	1.07103900	-6.25378000
H	-6.51529800	1.64164400	-4.77221400
H	-6.96248000	-0.61173100	-4.00786400
H	-6.11573600	-2.61988700	-5.23939000
H	-7.39660000	-1.80700500	-6.14511900
H	-5.70395600	-1.59763400	-6.62456900
H	-4.63371100	-1.33428200	-3.62518700
H	-4.07310200	-0.38101200	-5.00584500
H	-4.71299100	0.43248000	-3.57234300
C	-10.43003700	0.48693600	-3.38326100
C	-10.40488200	1.25682500	-2.04967400
C	-9.11704400	0.93108500	-1.31032100
C	-9.12169600	0.10336800	-0.17938400
C	-7.87788700	1.35172000	-1.81495600
C	-7.93153800	-0.33517900	0.40573100
C	-6.68425600	0.91368700	-1.23996300
C	-6.70569500	0.04952600	-0.14185600
H	-10.10526800	-0.54165700	-3.17575800
H	-10.48638900	2.33585000	-2.22911000
H	-11.26073800	0.97174600	-1.42872300
H	-10.07254100	-0.23021500	0.22831000
H	-7.84452700	2.00967500	-2.67906400
H	-7.94261200	-1.00879700	1.25747100
H	-5.73690700	1.23126000	-1.66339600
H	-5.78239900	-0.36610400	0.24975200
C	-2.17538700	-2.62338900	7.88196800
C	-2.20172300	-2.04655300	6.47484700
O	-2.35072700	-2.75534300	5.47230200
H	-2.00324300	-3.69681700	7.83007500
N	-2.08919400	-0.69312900	6.41805900
C	-2.01105900	0.13240600	5.20112700
C	-3.36261200	0.23262900	4.46054300
O	-3.93390800	1.32278400	4.33367300
C	-0.87895800	-0.32409500	4.27910100
H	-1.99629000	-0.20216700	7.29422400
H	-1.81324000	1.14901700	5.54465100
H	-0.75398200	0.39586200	3.46700600
H	0.06101400	-0.38786800	4.82473000
H	-1.08585200	-1.30957100	3.86742200
N	-3.83312600	-0.92799400	3.97478400
C	-5.15077500	-1.07756300	3.37810100
C	-5.63246500	-2.52512100	3.26162500
O	-6.64016700	-2.76843500	2.60232100
H	-3.23626700	-1.73916100	4.10429800
H	-5.87600800	-0.50325800	3.96387100
H	-5.16308400	-0.68385800	2.36110900
N	-4.90667000	-3.47318400	3.89635300
C	-5.12014500	-4.89453000	3.67314800
H	-4.12802100	-3.23266000	4.49285500
H	-5.13453300	-5.11016000	2.60183400
C	-8.77677100	-5.37476200	0.23173800
C	-7.41783500	-5.03663800	-0.39366300
C	-7.21402900	-3.56783700	-0.80364800
C	-5.82196000	-3.37797100	-1.41722000

C	-8.28711600	-3.06386100	-1.77719900
H	-9.58985600	-5.11860500	-0.45786600
H	-6.62026700	-5.30588500	0.31041900
H	-7.27111200	-5.67210300	-1.27993500
H	-7.26033000	-2.96647200	0.11027500
H	-5.64598900	-2.32415000	-1.65518200
H	-5.03632800	-3.69610600	-0.72783600
H	-5.72500400	-3.95309600	-2.34771300
H	-8.07381000	-2.03431100	-2.08105600
H	-8.31480000	-3.68454500	-2.68252300
H	-9.28753400	-3.07023000	-1.33380200
C	1.75848000	-5.81076500	3.28317800
O	1.36397400	-5.83242700	4.44808400
N	1.47576600	-4.77306200	2.43148000
C	0.87815000	-3.55245000	2.93807700
C	0.10107900	-2.80977100	1.84776000
S	-1.59631200	-3.48950400	1.60009500
O	4.91722100	-3.35810700	-3.30629500
C	-1.76299800	-0.34592600	-0.40333300
C	-2.19411900	-0.69392600	1.00553100
C	-2.67908300	-2.12767600	1.15597000
O	-3.84841300	-2.41234200	0.96589100
C	-0.63669600	0.34791400	-0.60100100
C	-2.70339000	-0.71951500	-1.51179300
H	-3.69286900	-0.28723800	-1.32885400
H	-2.34279200	-0.36562200	-2.47870600
H	-2.85418600	-1.80239900	-1.56906200
H	-0.00212700	0.64285700	0.22851500
H	-1.38508900	-0.46697700	1.69364200
H	-0.32848400	0.66674500	-1.58963500
H	0.65370200	-2.86440200	0.90999500
H	0.00876900	-1.76244300	2.12199200
H	0.24542300	-3.81267400	3.78953800
H	1.65016000	-2.86262600	3.30847800
H	2.06228600	-4.66059300	1.60614000
C	2.58555800	-6.92882500	2.66743800
H	2.19892500	-7.21201200	1.68347000
H	3.61848700	-6.59861000	2.50259500
H	2.57840400	-7.78579500	3.34044900
H	-0.71349000	-4.92935700	-6.18717400
H	1.04410300	-5.02624200	-5.74127100
H	5.82190400	-6.88835800	-2.79437400
H	8.13320400	-1.53263000	-4.61444100
H	9.68900200	-2.23404900	-3.97615900
H	8.20113300	0.29652200	-4.27862000
H	8.42772300	1.89931800	-5.09861200
H	0.89289200	2.57181400	7.38864200
H	2.66649900	2.63415900	7.48298200
H	6.68404800	6.69637900	-0.46097600
H	4.86999200	6.77929100	-0.34796400
H	3.13574900	9.26542400	-1.99744900
H	3.91929500	7.70387300	-1.64859300
H	3.16300100	8.76551700	2.78822300
H	4.25603800	8.11311100	4.07672400
H	-2.19346400	8.26357200	3.61691500
H	-2.54078700	7.24528900	2.19462800
H	-5.36414400	5.56565900	-4.88642600
H	-6.73448900	4.77798900	-4.02224600
H	-8.70388000	0.45989600	-5.58734100
H	-8.17376000	1.93042300	-6.49189500

H	-11.39481000	0.36360100	-3.88672900
H	-9.66988500	0.88177700	-4.06504700
H	-8.96395600	-4.81806200	1.15609300
H	-8.85085800	-6.45547200	0.39358400
H	-4.27161200	-5.40235800	4.14323700
H	-6.06093700	-5.24086200	4.11411900
H	-3.14971100	-2.42273400	8.34042300
H	-1.39169700	-2.13641300	8.47155800
H	0.25554300	0.21246300	-6.50627100
H	0.46119200	1.91543600	-5.88970000
H	3.96168800	-3.37106500	-3.12706900
H	5.14865500	-2.40276200	-3.36192800
O	-4.14824600	4.55152800	1.23727200
H	-4.29380100	3.59575700	1.40806000
H	-3.72814800	4.55808500	0.36848800
O	4.31768100	1.20953900	-5.29176100
H	4.70627200	0.52598600	-4.70111600
H	3.38479300	0.95157400	-5.30109000
O	-4.53055100	1.80657500	1.66227600
H	-4.48318200	1.69147400	2.63606900
H	-5.44084100	1.59877000	1.40680300

Direct decarboxylation mechanism: Deprotonated carboxylate group of MG-CoA
Protonated Glu72$_B$

E:S (0.0)

C	3.33972800	8.22891900	-1.41232400
C	2.47762700	7.61257300	-2.52867900
C	2.23580500	6.12297400	-2.38922700
C	1.40990540	5.61557337	-1.37678594
C	2.80622014	5.21683989	-3.29360322
C	1.13899678	4.25014490	-1.28176776
C	2.55011169	3.84791150	-3.19646324
C	1.71283475	3.36161228	-2.19239228
H	2.84504600	8.14597100	-0.44030200
H	1.50990700	8.12747900	-2.55946800
H	2.95708100	7.80067400	-3.49695000
H	0.96022349	6.30069387	-0.66432577
H	3.44301105	5.59443084	-4.09039534
H	0.45471167	3.88147858	-0.52447145
H	2.98449340	3.15818434	-3.91582821
H	1.51312059	2.29970756	-2.11481700
C	6.07515000	6.07542600	0.16056800
C	5.89469000	4.71937700	-0.52946700
C	4.69687900	3.94197300	0.02769900
S	4.93758000	3.25484300	1.71285700
C	6.08516800	1.88321900	1.34374500
H	6.11596400	5.92712200	1.24482600
H	5.71868100	4.87272000	-1.60350200
H	6.81098500	4.11971600	-0.44863000
H	3.83430800	4.60342400	0.12758000
H	4.40382200	3.12004300	-0.62941600
H	6.24051400	1.33602000	2.27596200
H	5.66676200	1.19384100	0.60627500
H	7.05319800	2.24567300	0.98777600
C	-1.84200500	7.20583300	3.41413900
C	-0.41318200	6.71124100	3.57665500
O	0.31226700	7.19066300	4.46244300
H	-2.50753000	6.53921900	3.97460200
N	0.02661900	5.70905000	2.77478200
C	1.32494400	5.06724900	3.06495700
C	2.49280200	5.97701700	2.65392600
O	2.89071800	6.06761900	1.49099800
C	1.29023100	3.75937600	2.26485800
C	-0.19996800	3.49859500	2.03757300
C	-0.76957200	4.90374400	1.83486000
H	1.38171100	4.88450800	4.14388800
H	1.79000400	3.92655400	1.30774300
H	1.81479800	2.95368200	2.78274300
H	-0.38244500	2.86917300	1.17178000
H	-0.67317100	3.03215400	2.90799400
H	-0.59323100	5.24754800	0.80846200
H	-1.84012700	4.96877300	2.02838000
N	3.04252000	6.64517400	3.69739400
C	4.01436600	7.70500400	3.47237300
H	2.37741800	6.79227300	4.45340900
H	4.71703000	7.39200800	2.70069600
C	1.89686900	1.72673500	7.25614000
C	1.92184500	1.44547000	5.73611700
C	2.76572000	0.26353300	5.32068700
C	2.25557700	-1.03818000	5.44606600
C	4.05365300	0.42938800	4.79746200
C	3.00991500	-2.14468500	5.05886200
C	4.81710600	-0.67763700	4.41951400
C	4.29839200	-1.96597000	4.55041500
H	1.85723800	0.79718200	7.83101200
H	0.89189800	1.26409400	5.40795400
H	2.25585500	2.34467700	5.20640800
H	1.24933100	-1.17905900	5.83318300
H	4.45357200	1.43293900	4.67619700
H	2.58860800	-3.14322900	5.12658300
H	5.81645700	-0.53343300	4.01786500
H	4.88644400	-2.82504100	4.24825300
C	0.13962000	-4.75772800	-5.58662000
C	-0.02234700	-3.63327400	-4.59286000
O	-0.00294200	-2.44878000	-4.92849700
H	-0.07020500	-5.72647900	-5.12150700
N	-0.22759700	-4.03706800	-3.30465400
C	-0.08767300	-3.08633300	-2.23300700
C	1.39290200	-2.84557600	-1.93011900
O	2.25848100	-3.30701600	-2.67996800
H	0.04680600	-4.98456600	-3.08029900
H	-0.54135000	-2.14276900	-2.54094500
H	-0.62392100	-3.43388300	-1.34916300
N	1.67731500	-2.09298700	-0.85633700
C	3.04859400	-1.77144100	-0.51209300
C	3.79630100	-3.03191800	-0.03430100
O	3.20320400	-4.01658400	0.42758600
C	3.11291700	-0.63797600	0.53987900
C	2.71822000	0.70392700	-0.08046600
C	2.28640500	-0.94335900	1.79245000
H	0.91971900	-1.75263400	-0.28155600
H	3.54694800	-1.42536600	-1.42392400
H	4.16330400	-0.58059300	0.84585300
H	2.81474100	1.50986500	0.65121600
H	3.35126700	0.94546800	-0.93969200

H	1.68608400	0.68482000	-0.43996800
H	2.48523500	-0.19641600	2.56254300
H	1.20906300	-0.91128700	1.59102400
H	2.52758800	-1.92091100	2.21162700
N	5.13258100	-2.94851100	-0.14259000
C	6.05819600	-3.95251300	0.39235000
C	5.76545500	-5.37174600	-0.14558200
O	5.76619700	-6.34580300	0.60197100
C	6.08077200	-3.93628300	1.91712900
H	5.53036300	-2.14828100	-0.62375500
H	7.03685300	-3.67492000	-0.01251900
H	6.78015000	-4.68661800	2.28807900
H	6.37844600	-2.94660100	2.27479200
H	5.08980200	-4.17626500	2.30669300
N	5.59938400	-5.43317800	-1.49066400
C	5.18838000	-6.65846000	-2.15483800
C	3.73861500	-6.53506000	-2.65081900
O	2.79885700	-6.33855600	-1.60604100
H	5.55313400	-4.56900700	-2.03469400
H	5.26306500	-7.45982900	-1.41669100
H	3.68184700	-5.72292700	-3.39161700
H	3.45062400	-7.46541100	-3.15456100
H	3.06814600	-5.56156700	-1.08783800
C	0.18877800	1.22163400	-5.78784400
C	0.14694100	0.66824400	-4.37132600
S	1.76562000	0.02866100	-3.75905800
H	-0.83666800	1.49118000	-6.07459800
H	-0.15911600	1.44073000	-3.66210000
H	-0.54133500	-0.17442500	-4.29786700
H	1.60160400	-1.21596400	-4.25861500
C	8.87303000	-2.46239400	-4.39044900
C	8.08257000	-3.39330900	-3.46585300
O	8.10307530	-2.95009364	-2.10991698
H	9.02845600	-2.93685300	-5.36692300
H	7.05557117	-3.52516435	-3.82337782
H	8.55161938	-4.38283385	-3.45536705
H	7.56119279	-2.15056491	-2.02954663
C	8.00483900	1.26544000	-4.38486300
C	7.55532000	1.97099700	-3.08897600
C	6.13181800	1.60490300	-2.61197800
C	5.96129400	0.11191900	-2.49386200
O	6.31061700	-0.52084400	-1.50529900
O	5.47249800	-0.52937200	-3.55624800
H	7.16139700	1.11152800	-5.06734900
H	8.26061600	1.74605100	-2.28250700
H	7.57027700	3.05779800	-3.22038000
H	5.95032200	2.04504300	-1.63248800
H	5.39315600	2.00625600	-3.30892400
C	-5.30003900	5.20651300	-4.00511500
C	-4.74789000	5.65947900	-2.64311000
C	-3.22024100	5.88398200	-2.68744500
C	-5.15180400	4.70149200	-1.51913400
C	-2.37470800	4.64662200	-3.01689200
H	-4.91390100	4.21675600	-4.27559000
H	-5.19978000	6.63877700	-2.42458200
H	-3.00540300	6.67282400	-3.42268400
H	-2.90205100	6.27583700	-1.71269500
H	-4.89151100	5.09138600	-0.53160300
H	-6.22974700	4.50808000	-1.53033100
H	-4.64105600	3.74018600	-1.61932400

H	-1.31711900	4.91939300	-3.08721500
H	-2.46021000	3.87921200	-2.24271700
H	-2.66222400	4.20531100	-3.97795100
C	-7.64297300	1.35653400	-6.34431300
C	-6.29649800	1.24148000	-5.62122500
C	-6.00015800	-0.14525000	-5.02796300
C	-6.10135400	-1.27049100	-6.06381800
C	-4.61940800	-0.14912200	-4.36837000
H	-7.63116000	0.81287900	-7.29519200
H	-5.48602600	1.51744600	-6.31150700
H	-6.26045900	1.97356500	-4.80452900
H	-6.74777200	-0.33595900	-4.24466800
H	-5.86226100	-2.23660400	-5.60729700
H	-7.10524400	-1.34669400	-6.49183800
H	-5.39561300	-1.10567300	-6.88824400
H	-4.41723000	-1.10869900	-3.88605300
H	-3.83144000	0.02614100	-5.11247300
H	-4.54315300	0.63353300	-3.60837000
C	-10.20049000	0.90292000	-3.55857700
C	-10.19099500	1.64130200	-2.21055700
C	-8.90495100	1.30683000	-1.47837700
C	-8.89010307	0.35624295	-0.45060770
C	-7.68201283	1.84789814	-1.89892976
C	-7.69303906	-0.06273549	0.13416436
C	-6.48244005	1.43429922	-1.31972644
C	-6.48302620	0.47258510	-0.30898624
H	-9.90245900	-0.13722100	-3.37127600
H	-10.27977000	2.72352100	-2.36627400
H	-11.04911500	1.33440100	-1.60269600
H	-9.82870295	-0.08030674	-0.11837631
H	-7.66687685	2.59603782	-2.68795205
H	-7.70176516	-0.81888602	0.91435140
H	-5.54604746	1.86982750	-1.64833779
H	-5.54288412	0.15336186	0.12851605
C	-2.21488300	-2.83336400	7.71091200
C	-2.19237200	-2.28191800	6.29522800
O	-2.42598900	-2.98492200	5.31482000
H	-2.07606800	-3.91372900	7.66665500
N	-1.89642100	-0.94669600	6.19162100
C	-2.24420700	-0.22336000	4.96013900
C	-3.72231600	-0.54405900	4.63747000
O	-4.53784600	-0.61962800	5.55532800
C	-1.23802400	-0.49206500	3.84439400
H	-2.00276600	-0.41822400	7.04564700
H	-2.22791000	0.84475600	5.19667900
H	-1.45603900	0.14058700	2.98143300
H	-0.22741600	-0.26769700	4.19243800
H	-1.27722500	-1.54284100	3.55450500
N	-4.04968900	-0.75784100	3.33976700
C	-5.41442400	-1.08102300	2.97333000
C	-5.77120300	-2.57070900	2.90197700
O	-6.80902300	-2.90807400	2.32609500
H	-3.42854500	-0.45432700	2.59492500
H	-6.09836900	-0.62063600	3.69238100
H	-5.61813600	-0.67286000	1.98743200
N	-4.94263200	-3.44080800	3.51783000
C	-5.14250600	-4.87044900	3.37218500
H	-4.08491200	-3.11252200	3.94676100
H	-5.15644300	-5.13443700	2.30810500
C	-8.75118700	-5.12999900	-0.14268800

C	-7.40469200	-4.77469300	-0.77761800
C	-7.23018800	-3.29768300	-1.17512400
C	-5.91200100	-3.10857900	-1.93237300
C	-8.38787100	-2.75381700	-2.02176900
H	-9.56713300	-4.84370500	-0.81863100
H	-6.59198100	-5.03556000	-0.08947600
H	-7.26793300	-5.39790300	-1.67510200
H	-7.17207300	-2.71995900	-0.24685300
H	-5.72269700	-2.04553800	-2.11001000
H	-5.07137100	-3.50890800	-1.36173600
H	-5.94773900	-3.61416900	-2.90752200
H	-8.17576900	-1.72823000	-2.33934600
H	-8.53449000	-3.36485700	-2.92286600
H	-9.33308500	-2.73215600	-1.47158300
C	1.40391300	-6.22019400	2.97295500
O	0.41756700	-6.71572300	3.50735800
N	1.53121700	-4.85737600	2.78664400
C	0.28762100	-4.09758400	2.78022600
C	-0.45020500	-4.36190900	1.45604900
S	-2.27415700	-4.28617800	1.53517800
O	4.88802700	-3.35880700	-3.35232900
C	-1.94615500	-0.71185800	-0.38253200
C	-1.78136700	-1.66734900	0.55858900
C	-2.76548900	-2.74600900	0.77084900
O	-3.95358700	-2.61923400	0.51560700
C	-1.27391400	0.61437700	-0.25054400
C	-2.30859200	1.60185500	0.40522100
O	-2.44642100	2.72204200	-0.12624600
O	-2.87496100	1.15660700	1.44624600
C	-2.91215500	-0.80987300	-1.52432000
H	-3.76477100	-0.14511200	-1.35280600
H	-2.42810700	-0.46289900	-2.44395900
H	-3.30736300	-1.81161400	-1.66998500
H	-0.41742000	0.57252100	0.43018400
H	-1.02652000	-1.54056500	1.32661300
H	-0.95345900	1.02743400	-1.20999800
H	-0.25150800	-5.39198900	1.15419900
H	-0.07503500	-3.71696900	0.66056200
H	-0.32095900	-4.42816800	3.62195900
H	0.50410600	-3.03557100	2.91312900
H	2.22083600	-4.56814100	2.09591600
C	2.52754400	-7.05666300	2.41276400
H	2.36248700	-7.20201700	1.33785400
H	3.50822600	-6.58107800	2.50546500
H	2.53251600	-8.03083400	2.90196200
H	-0.57220000	-4.64459600	-6.41090700
H	1.17480000	-4.80192200	-5.94113400
H	5.85559400	-6.89170200	-2.99120800
H	8.32652200	-1.53060200	-4.56963600
H	9.85390400	-2.29402000	-3.93376900
H	8.43325500	0.28239300	-4.16363200
H	8.71235700	1.90874600	-4.91845900
H	0.98676200	2.29921200	7.46357400
H	2.75945100	2.31372600	7.58843700
H	7.00775900	6.57014300	-0.13040700
H	5.19452700	6.69376000	-0.04322800
H	3.54902200	9.28259600	-1.62437500
H	4.28882900	7.69025500	-1.32283200
H	3.48354100	8.60264500	3.13845300
H	4.53855800	7.87534400	4.41849000
H	-1.89691400	8.20346200	3.86222000
H	-2.24467600	7.24831400	2.39675200
H	-4.98753000	5.90644500	-4.78709000
H	-6.39125200	5.12085400	-3.97526400
H	-8.43832000	0.91556900	-5.73438100
H	-7.85753100	2.40547300	-6.57389400
H	-11.15905100	0.82249200	-4.08178100
H	-9.41946200	1.30403800	-4.21279700
H	-8.94014600	-4.60367600	0.79875800
H	-8.85399700	-6.21363100	-0.02349600
H	-4.31439100	-5.41641500	3.83601600
H	-6.09856400	-5.20949300	3.78449600
H	-3.19144400	-2.62544600	8.16050400
H	-1.42947800	-2.38787700	8.33035700
H	0.52623600	0.47974100	-6.51907900
H	0.76311900	2.15278900	-5.83512000
H	3.94295700	-3.33371000	-3.10555800
H	5.13404800	-2.43147500	-3.47808400
H	5.13350100	0.08844000	-4.27415300
O	-3.87464600	4.56068900	1.60140000
H	-3.89430500	4.01182300	2.40685800
H	-3.49227000	3.94912300	0.93952400
O	4.30735600	0.91257500	-5.38409200
H	4.37281100	1.86694200	-5.51061800
H	3.38430600	0.74049100	-5.07312700
O	-3.44705200	2.55819900	3.64404800
H	-4.29470400	2.20111300	3.93944100

TS1 (+18.8)

C	3.05913700	8.21565400	-1.72009900
C	2.22985500	7.53603200	-2.82447000
C	2.05894200	6.04159700	-2.64655100
C	1.13157100	5.52668300	-1.73073800
C	2.80952500	5.13621800	-3.40826500
C	0.94097500	4.15187100	-1.60011100
C	2.62871400	3.75802200	-3.27278200
C	1.68829600	3.26060900	-2.37145700
H	2.56087900	8.15159200	-0.74875300
H	1.23945200	8.00534700	-2.87166700
H	2.70439100	7.72198700	-3.79527700
H	0.55669300	6.21009100	-1.11339200
H	3.53185400	5.52017000	-4.12485800
H	0.20136800	3.77129300	-0.90531800
H	3.20804800	3.06756500	-3.88023800
H	1.54478200	2.19317500	-2.25911900
C	5.85104500	6.19827000	-0.06840400
C	5.71176900	4.81678200	-0.71355200
C	4.53174700	4.02693300	-0.14020700
S	4.75612800	3.44163900	1.58437400
C	6.00596000	2.13532500	1.33003000
H	5.89376700	6.08839100	1.02023600
H	5.53945600	4.92755800	-1.79350900
H	6.64272200	4.24492200	-0.60622500
H	3.64387300	4.66020100	-0.09560100
H	4.28646700	3.16179900	-0.75887800
H	6.14879400	1.64034900	2.29253800
H	5.66790500	1.38813900	0.60785700
H	6.96463600	2.54624200	1.00295600
C	-2.09830900	7.22852200	3.13970600

C	-0.65935400	6.78295100	3.31153500
O	0.06021300	7.30880500	4.17286600
H	-2.74679500	6.56292500	3.72008800
N	-0.19838700	5.76815600	2.53443200
C	1.11706500	5.16955600	2.84344300
C	2.26324300	6.09937400	2.40798600
O	2.65098300	6.16989900	1.24080900
C	1.11001600	3.83661700	2.08590000
C	-0.37833600	3.51868300	1.91842700
C	-0.99935800	4.89370900	1.66193700
H	1.17584900	5.01820400	3.92726400
H	1.57908300	3.98734700	1.11116700
H	1.67286700	3.06480100	2.61356900
H	-0.56161100	2.81679400	1.10615100
H	-0.79954300	3.09680400	2.83713500
H	-0.87167200	5.19060000	0.61242700
H	-2.06249800	4.94021100	1.90325400
N	2.80124300	6.80688700	3.43269900
C	3.74196300	7.89122800	3.18072600
H	2.13881000	6.95088000	4.19083300
H	4.45768600	7.57583000	2.42188600
C	1.78573100	2.00041300	7.18219700
C	1.82624300	1.65445200	5.67784200
C	2.78405700	0.54657500	5.30735900
C	2.39349900	-0.79376000	5.44761200
C	4.06570900	0.82053500	4.81523900
C	3.25268300	-1.83395100	5.09649100
C	4.93591900	-0.21870700	4.47922500
C	4.53186600	-1.54689600	4.61658300
H	1.77427800	1.09439500	7.79538500
H	0.81756100	1.35631000	5.36822800
H	2.06555300	2.55826900	5.10580900
H	1.39774700	-1.02102100	5.81999100
H	4.37671000	1.85390000	4.68354200
H	2.91802000	-2.86442800	5.16974700
H	5.92981600	0.00877800	4.10391000
H	5.20149500	-2.35361400	4.34123500
C	0.22428100	-4.99967300	-5.41274300
C	0.05476700	-3.81133400	-4.49630600
O	0.07600700	-2.65541700	-4.92422500
H	0.03635500	-5.94307500	-4.89076900
N	-0.14593600	-4.11755900	-3.18414500
C	0.02149500	-3.09955400	-2.17786100
C	1.50603100	-2.89646100	-1.88202500
O	2.35608200	-3.39587900	-2.62906300
H	0.10673000	-5.05395000	-2.89714900
H	-0.37917100	-2.15866200	-2.55710800
H	-0.54539600	-3.33812500	-1.27710200
N	1.81209200	-2.12806300	-0.82636100
C	3.18879100	-1.79378900	-0.52327400
C	3.94278800	-3.01556900	0.03130100
O	3.36048800	-4.00664300	0.49217400
C	3.29026100	-0.58769800	0.43970400
C	2.88311500	0.70202900	-0.27278200
C	2.50249700	-0.79557500	1.73704500
H	1.05278600	-1.74876500	-0.27597600
H	3.67791900	-1.52330300	-1.46500800
H	4.34943300	-0.51523600	0.70798700
H	2.93870700	1.55358700	0.41063200
H	3.53706300	0.90187700	-1.12726100
H	1.86490000	0.62980300	-0.65866600
H	2.73477700	-0.00034500	2.44775400
H	1.42120400	-0.76702000	1.56758600
H	2.74826200	-1.74568100	2.21501600
N	5.28045000	-2.89664700	-0.01212600
C	6.19880200	-3.87743900	0.57088300
C	5.92040700	-5.30907800	0.06297400
O	5.95502600	-6.27042100	0.82687100
C	6.18760400	-3.81945300	2.09522200
H	5.67789800	-2.10624900	-0.50820600
H	7.18537100	-3.60631300	0.18105100
H	6.85940900	-4.57699000	2.50084900
H	6.50360000	-2.82788200	2.43135300
H	5.18229100	-4.02223000	2.47055800
N	5.72454700	-5.39291800	-1.27521900
C	5.31931900	-6.63215000	-1.91199700
C	3.87123400	-6.53946600	-2.41722200
O	2.92378600	-6.34832100	-1.38104600
H	5.66129000	-4.54017900	-1.83479800
H	5.39907300	-7.41734000	-1.15699300
H	3.80287400	-5.74152400	-3.17174100
H	3.60667800	-7.48328500	-2.90942200
H	3.16476900	-5.54784000	-0.88405200
C	0.10857900	0.96722100	-5.83452700
C	0.11670300	0.45308700	-4.40213000
S	1.76963200	-0.12555000	-3.82641600
H	-0.92898600	1.19180000	-6.11540600
H	-0.19019800	1.22908100	-3.69829800
H	-0.54486200	-0.40562500	-4.28716500
H	1.62361700	-1.38632400	-4.29153700
C	8.88937200	-2.42118600	-4.30013800
C	8.13739900	-3.34923600	-3.34198900
O	8.04421200	-2.80360100	-2.02712100
H	9.06050900	-2.92362700	-5.26006200
H	7.14887300	-3.60830900	-3.73467200
H	8.69661400	-4.28416200	-3.22930700
H	7.43726000	-2.04800900	-2.04155900
C	7.91843100	1.27886200	-4.43279600
C	7.45679900	2.02820300	-3.16686900
C	6.06305400	1.62012000	-2.63989200
C	5.96567500	0.12602600	-2.46858000
O	6.35640700	-0.45490600	-1.46423600
O	5.49828500	-0.57111900	-3.50466000
H	7.07912800	1.07662000	-5.10733700
H	8.18795900	1.87903700	-2.36575700
H	7.41807800	3.10619600	-3.35501900
H	5.88838400	2.08461600	-1.67074600
H	5.28738300	1.96418300	-3.32779700
C	-5.49061400	4.86183700	-4.20139100
C	-4.96128400	5.39044000	-2.85747700
C	-3.42977300	5.59286300	-2.87418100
C	-5.41474800	4.51252200	-1.68612100
C	-2.58908400	4.34016000	-3.15228400
H	-5.07446100	3.87382300	-4.42775700
H	-5.40257000	6.38701200	-2.70783500
H	-3.18717400	6.35752700	-3.62514500
H	-3.12880100	6.01136200	-1.90467500
H	-5.11334800	4.94807700	-0.72719800
H	-6.50350300	4.39765500	-1.67346800
H	-4.98193900	3.51176700	-1.74265000

H	-1.52217700	4.58239900	-3.11931700
H	-2.76841400	3.55900000	-2.40983900
H	-2.79984500	3.92196600	-4.14219600
C	-7.72339700	0.86491700	-6.39732300
C	-6.37359400	0.80500000	-5.67060400
C	-6.07983500	-0.53045000	-4.96644200
C	-6.20851400	-1.73861500	-5.90123000
C	-4.68993600	-0.49668400	-4.32640800
H	-7.69877700	0.28593400	-7.32689200
H	-5.56398700	1.02421800	-6.38183900
H	-6.33377700	1.59978100	-4.91439300
H	-6.81815900	-0.64496700	-4.16035100
H	-5.97639400	-2.66438800	-5.36493700
H	-7.21864200	-1.83720300	-6.31008300
H	-5.51048700	-1.65519100	-6.74417800
H	-4.50586100	-1.39491900	-3.73156100
H	-3.90775900	-0.43394500	-5.09394500
H	-4.57899300	0.36708700	-3.66546300
C	-10.27076500	0.44378000	-3.59730900
C	-10.26558900	1.22357400	-2.27056900
C	-8.97198400	0.92679500	-1.53057800
C	-8.95564700	0.06140300	-0.42930300
C	-7.74499200	1.40652700	-2.01163900
C	-7.75528800	-0.34627900	0.15770400
C	-6.54186900	0.99699900	-1.43495700
C	-6.54081000	0.10607300	-0.36031300
H	-9.94425000	-0.58167300	-3.37832700
H	-10.36916400	2.29942900	-2.45929300
H	-11.11704300	0.92538800	-1.64927300
H	-9.89689300	-0.32194100	-0.04286700
H	-7.72902100	2.09022300	-2.85720900
H	-7.75643900	-1.03930900	0.99453300
H	-5.60076200	1.36394400	-1.83003800
H	-5.60336400	-0.25614300	0.05076500
C	-2.19901200	-2.65178200	7.80415600
C	-2.20117700	-2.16777500	6.36412200
O	-2.52219900	-2.90589100	5.43723600
H	-2.03324400	-3.72923400	7.80375100
N	-1.83718800	-0.86183600	6.18149900
C	-2.03868100	-0.16589800	4.89981500
C	-3.54401700	-0.20873600	4.55687100
O	-4.33629000	0.42033600	5.27500900
C	-1.07874300	-0.65494300	3.82146900
H	-1.81582700	-0.27891100	7.00488500
H	-1.84690700	0.89134800	5.10184600
H	-1.23699700	-0.09991800	2.89624500
H	-0.04679100	-0.50512500	4.14470300
H	-1.22155500	-1.71717700	3.62394500
N	-3.91545400	-0.93664200	3.49387300
C	-5.29476200	-1.14855700	3.10632600
C	-5.69971700	-2.62518700	3.03274000
O	-6.71885500	-2.95008800	2.42074900
H	-3.20801900	-1.36831200	2.91637300
H	-5.92462800	-0.63075200	3.83295000
H	-5.47792400	-0.72837700	2.11797600
N	-4.90537400	-3.49314400	3.69467600
C	-5.06387300	-4.92771600	3.54294300
H	-4.05759600	-3.16042300	4.14054500
H	-5.05859400	-5.19637700	2.48138500
C	-8.65961100	-5.41652500	0.03933300
C	-7.29912100	-5.05605900	-0.56851400
C	-7.13725900	-3.59069000	-1.00846400
C	-5.73445600	-3.35741900	-1.57903100
C	-8.19688700	-3.14695400	-2.02530200
H	-9.46717000	-5.16773000	-0.66033300
H	-6.50876000	-5.27927400	0.15827500
H	-7.11532700	-5.70657200	-1.43759600
H	-7.23569100	-2.97324000	-0.11018300
H	-5.60912400	-2.31002800	-1.87078700
H	-4.95987100	-3.57572300	-0.84103400
H	-5.56818400	-3.97980100	-2.46973500
H	-8.01766500	-2.11132100	-2.33158300
H	-8.16419800	-3.77656400	-2.92499100
H	-9.21246600	-3.19407800	-1.61956600
C	1.57286800	-6.03317900	3.20071800
O	0.65643400	-6.45487700	3.90030700
N	1.65876500	-4.71571900	2.81160700
C	0.44050100	-3.91654500	2.85100800
C	-0.43614800	-4.24338700	1.63353100
S	-2.20080600	-3.77945500	1.77545200
O	4.98304900	-3.39961800	-3.20684200
C	-1.55186400	-0.37481300	-0.54204400
C	-1.41536200	-1.44435200	0.38993100
C	-2.50271500	-2.26553200	0.76119800
O	-3.71226300	-2.08116300	0.54552700
C	-0.78080200	0.76419600	-0.48498300
C	-2.80314100	1.79830400	0.62771000
O	-3.13301400	2.55067400	-0.22187600
O	-2.79421200	1.23443300	1.66840400
C	-2.60097800	-0.46863900	-1.62862000
H	-3.60291300	-0.46783300	-1.19822700
H	-2.51012600	0.35674100	-2.33840000
H	-2.51909100	-1.41316800	-2.17473100
H	-0.02659900	0.88227300	0.28509600
H	-0.53234200	-1.47802500	1.01993100
H	-0.77303000	1.48405000	-1.29608400
H	-0.44733200	-5.32900900	1.50896200
H	-0.01117400	-3.80949600	0.72625100
H	-0.09512200	-4.16185900	3.76900500
H	0.70388400	-2.85703500	2.87748200
H	2.30426400	-4.50251900	2.05438000
C	2.66481700	-6.93492300	2.66661800
H	2.42211000	-7.21845700	1.63520700
H	3.64307900	-6.44671000	2.62308500
H	2.71846600	-7.83606300	3.27762600
H	-0.48945300	-4.93680600	-6.24078600
H	1.26065300	-5.02831200	-5.76510900
H	5.99374900	-6.87754800	-2.73902700
H	8.31765400	-1.51209800	-4.51396600
H	9.86471700	-2.20897500	-3.84991900
H	8.37360000	0.31698900	-4.17459800
H	8.60876800	1.92140500	-4.98924800
H	0.85991300	2.55477800	7.36818400
H	2.63137700	2.62291700	7.49280400
H	6.77009400	6.70736300	-0.37691600
H	4.95394400	6.78393200	-0.29473600
H	3.23953200	9.26617100	-1.97090700
H	4.02271600	7.70717100	-1.61057500
H	3.18696300	8.76092500	2.81379500
H	4.26007600	8.11084000	4.12006300

H	-2.18130800	8.24009800	3.55062800
H	-2.50073100	7.22225200	2.12135800
H	-5.19655100	5.54087100	-5.00864400
H	-6.57903600	4.74726500	-4.16868400
H	-8.50695700	0.42486000	-5.77176600
H	-7.96651100	1.89828500	-6.66560600
H	-11.22615800	0.31770500	-4.11744700
H	-9.50039700	0.84200800	-4.26577700
H	-8.86425400	-4.86123300	0.96060000
H	-8.73262500	-6.49746300	0.19839400
H	-4.22155700	-5.43312900	4.02684400
H	-6.01069800	-5.27766800	3.96736900
H	-3.18150900	-2.45444600	8.24555700
H	-1.42699400	-2.16212700	8.40690700
H	0.46713200	0.20844900	-6.53789400
H	0.65680800	1.91157800	-5.91611000
H	4.02566200	-3.39659100	-3.00099800
H	5.20215200	-2.47268300	-3.37492600
H	5.13838800	0.00659400	-4.24536400
O	-3.31398100	2.82760900	4.01647100
H	-3.11220400	2.23511400	3.27503200
H	-3.86116300	2.26761600	4.59830300
O	4.31693700	0.75472700	-5.41098200
H	4.36416000	1.70255400	-5.58442100
H	3.38659900	0.57258200	-5.12327100
O	-4.24477600	4.65457700	2.05471700
H	-4.09773000	4.07265000	2.82742800

Int (+0.8)

C	3.33728800	8.21545200	-1.45626400
C	2.50070500	7.60250400	-2.59270600
C	2.27166300	6.11193600	-2.46155200
C	1.31780700	5.60459300	-1.56908800
C	2.99977500	5.20398800	-3.24151900
C	1.08112800	4.23390600	-1.47832900
C	2.77157100	3.82983800	-3.14723500
C	1.80601300	3.34034300	-2.26883600
H	2.81928400	8.14597000	-0.49580700
H	1.52993200	8.11145800	-2.63869300
H	2.99634800	7.79930900	-3.55070300
H	0.76430600	6.28849900	-0.93319000
H	3.74314100	5.58374000	-3.93853200
H	0.33055100	3.85757300	-0.79207300
H	3.33476000	3.13674000	-3.76651200
H	1.62842000	2.27576800	-2.18781500
C	6.01974800	6.05344900	0.19422700
C	5.83723800	4.69299400	-0.47938800
C	4.61491300	3.94777800	0.06172400
S	4.77409200	3.33895800	1.78527800
C	5.99420200	1.99850500	1.56723900
H	6.03891300	5.91931100	1.28099200
H	5.68851100	4.82889300	-1.55993500
H	6.74061600	4.07959800	-0.36599000
H	3.75153800	4.61616000	0.09394000
H	4.34876200	3.10087900	-0.57203500
H	6.04058800	1.45841800	2.51515400
H	5.68537200	1.29531400	0.79013900
H	6.98985300	2.38373300	1.33370000
C	-1.94602200	7.30294000	3.28134200
C	-0.52492000	6.80139500	3.46079100
O	0.22607100	7.34323300	4.28215800
H	-2.62441800	6.65306300	3.84758900
N	-0.12896200	5.72734300	2.73136900
C	1.18754900	5.11213800	3.00439000
C	2.35117100	6.04484900	2.59952100
O	2.72153900	6.16471900	1.43123200
C	1.15638200	3.81136600	2.19200000
C	-0.33424400	3.46784000	2.09861600
C	-1.00929500	4.83430900	1.95187600
H	1.25731700	4.90939100	4.08012600
H	1.57395500	4.01444600	1.20338700
H	1.76043000	3.03012400	2.65590300
H	-0.56646100	2.79975100	1.26653000
H	-0.69919600	2.99178100	3.01256100
H	-1.04927300	5.16108000	0.90366400
H	-2.02172500	4.81721100	2.35830700
N	2.93570900	6.67756200	3.65029000
C	3.91302700	7.74338600	3.44629600
H	2.29109700	6.80979500	4.42368400
H	4.63523900	7.42683300	2.69352500
C	1.66173900	1.83278100	7.25886400
C	1.70408800	1.53063900	5.74265400
C	2.59490200	0.37630700	5.34875900
C	2.08997700	-0.93261000	5.35910800
C	3.92878600	0.57569600	4.97241400
C	2.89555400	-2.01499700	5.00684600
C	4.74158000	-0.50677500	4.62988100
C	4.22831000	-1.80417100	4.64856600
H	1.60327100	0.91063800	7.84410800
H	0.68374000	1.30529200	5.41157500
H	2.00784200	2.43593600	5.20375800
H	1.05086700	-1.09943300	5.63227100
H	4.32774500	1.58671600	4.94030600
H	2.47922200	-3.01786200	4.98547300
H	5.77619200	-0.33686000	4.34410200
H	4.85561100	-2.64415900	4.37246700
C	0.08332100	-4.78727600	-5.53771700
C	-0.02450100	-3.59019000	-4.62110700
O	0.10832300	-2.44106200	-5.05020600
H	-0.15439000	-5.72378300	-5.02502800
N	-0.28485900	-3.87009400	-3.31484700
C	-0.05155200	-2.86609100	-2.30652600
C	1.43556500	-2.80732500	-1.95826300
O	2.25255300	-3.44589400	-2.63221500
H	-0.13206100	-4.82490700	-3.01892000
H	-0.34784700	-1.89466800	-2.70337500
H	-0.67059100	-3.03612400	-1.42309700
N	1.78250900	-2.02014600	-0.92782100
C	3.17265400	-1.79597200	-0.58372400
C	3.82010500	-3.07209400	-0.02240500
O	3.16341300	-4.02958300	0.40536500
C	3.32335800	-0.62784600	0.41904000
C	3.03348600	0.70526000	-0.26945700
C	2.48387300	-0.82447900	1.68691900
H	1.07043000	-1.42554200	-0.52655000
H	3.71721400	-1.53920600	-1.49906100
H	4.37552200	-0.63544800	0.72136000
H	3.05458800	1.52444400	0.45343600
H	3.77013400	0.91161800	-1.05074700

H	2.05671100	0.69601100	-0.75054500
H	2.74728600	-0.06938500	2.43050700
H	1.41067600	-0.72924900	1.49637500
H	2.65833000	-1.80375500	2.13606100
N	5.16372600	-3.02944800	-0.01133000
C	6.00049700	-4.06698600	0.59231600
C	5.67338300	-5.46684900	0.03285400
O	5.65944800	-6.45333200	0.76435000
C	5.91538000	-4.04554800	2.11524400
H	5.62380300	-2.25388500	-0.47443200
H	7.01796100	-3.83889400	0.25853000
H	6.52815600	-4.84590000	2.53180300
H	6.26074700	-3.07957600	2.49426600
H	4.88331000	-4.20739500	2.43316900
N	5.48976300	-5.49679800	-1.30916900
C	5.04558000	-6.69771200	-1.98732100
C	3.60693000	-6.54729100	-2.50198000
O	2.66139600	-6.35587900	-1.46477400
H	5.47499900	-4.62656200	-1.84313100
H	5.08727900	-7.50604800	-1.25368500
H	3.56413100	-5.72940700	-3.23608200
H	3.31903800	-7.47087300	-3.01898900
H	2.90200400	-5.55090100	-0.97519100
C	0.19804000	1.18836200	-5.80871800
C	0.17696400	0.63760500	-4.39017900
S	1.80981200	0.00221700	-3.81528500
H	-0.82760700	1.45520000	-6.09522900
H	-0.11827700	1.40073500	-3.66800000
H	-0.50779300	-0.20603900	-4.30544500
H	1.64261200	-1.23153900	-4.34486400
C	8.81552900	-2.56631800	-4.20096800
C	8.01913200	-3.49682400	-3.28237300
O	7.93792800	-2.99636800	-1.94889000
H	8.98744600	-3.04755200	-5.17166600
H	7.02478900	-3.70310300	-3.69047400
H	8.54128200	-4.45607000	-3.20012100
H	7.38488500	-2.20022300	-1.94543300
C	7.98611600	1.16993400	-4.25602000
C	7.54794000	1.91012200	-2.97688100
C	6.14479900	1.53355600	-2.45404800
C	6.00083800	0.03806700	-2.33585200
O	6.37662100	-0.58892200	-1.35425800
O	5.50770500	-0.60712500	-3.39397000
H	7.14751100	1.02032100	-4.94426200
H	8.27610800	1.72363700	-2.18089500
H	7.54249100	2.99209200	-3.14548400
H	5.99292700	1.96863800	-1.46743900
H	5.37584500	1.92783500	-3.12226800
C	-5.28216600	5.24972600	-4.17894600
C	-4.74303900	5.65326100	-2.79412800
C	-3.22253700	5.40233900	-2.66435300
C	-5.52881500	4.96446600	-1.67206300
C	-2.79940700	3.93100500	-2.54857100
H	-4.92195800	4.25049000	-4.45272700
H	-4.89371900	6.73701000	-2.68542400
H	-2.71474700	5.85804800	-3.52504900
H	-2.85649900	5.93801000	-1.77822800
H	-5.12376900	5.21524700	-0.68569200
H	-6.58267800	5.26084500	-1.68928200
H	-5.49738600	3.87663200	-1.76815400
H	-1.70733800	3.85050200	-2.54578300
H	-3.16472600	3.47186600	-1.62449500
H	-3.17057900	3.32902700	-3.38519300
C	-7.61971200	1.39595900	-6.51708900
C	-6.30775500	1.28865600	-5.72172100
C	-6.11630800	-0.04294400	-4.97555600
C	-6.18609100	-1.25710900	-5.90850600
C	-4.79894400	-0.03824300	-4.19484700
H	-7.57438300	0.83168800	-7.45394400
H	-5.45047600	1.45854600	-6.38920400
H	-6.27556000	2.09610900	-4.97829100
H	-6.93421700	-0.12856700	-4.24630500
H	-6.04910000	-2.18497800	-5.34393800
H	-7.14842100	-1.32244100	-6.42555700
H	-5.39729600	-1.20829300	-6.67017100
H	-4.69713700	-0.93952200	-3.58408500
H	-3.93933700	0.00687200	-4.87599100
H	-4.73926000	0.82238500	-3.52257800
C	-10.23442300	1.00162600	-3.77592900
C	-10.25523800	1.74577900	-2.42877100
C	-9.04318700	1.35352700	-1.60082700
C	-9.16843700	0.45543100	-0.53310400
C	-7.75718400	1.79621800	-1.94473100
C	-8.04908700	-0.00937500	0.16182200
C	-6.63505000	1.33400900	-1.25487900
C	-6.77438100	0.42116600	-0.20813200
H	-9.95099800	-0.03996400	-3.57419500
H	-10.27714600	2.83033200	-2.59368600
H	-11.16566200	1.49206000	-1.87468300
H	-10.15736700	0.09876600	-0.25516400
H	-7.63360800	2.50303000	-2.76231100
H	-8.15844900	-0.72534900	0.97171100
H	-5.64256900	1.67848300	-1.52640500
H	-5.89465500	0.03738700	0.29623300
C	-2.50483100	-2.67970600	7.68836600
C	-2.48222700	-2.14718200	6.26400100
O	-2.84288800	-2.84030500	5.31722200
H	-2.37842000	-3.76210400	7.65765800
N	-2.06522900	-0.85004900	6.11935500
C	-2.38950300	-0.08364100	4.89955700
C	-3.92132500	-0.15483700	4.70556400
O	-4.64972000	0.23309700	5.62350100
C	-1.52415600	-0.48113600	3.71125100
H	-2.05705200	-0.30572300	6.96992400
H	-2.19632300	0.96491300	5.13431900
H	-1.77585600	0.13590600	2.84672700
H	-0.46846400	-0.32054000	3.94625500
H	-1.66315300	-1.53099200	3.45542100
N	-4.38555200	-0.66702800	3.54746500
C	-5.78633700	-0.94571000	3.31980000
C	-6.08164900	-2.42274800	3.02991600
O	-7.10043500	-2.73823500	2.40737300
H	-3.76869800	-0.93709500	2.78897600
H	-6.33049200	-0.64212500	4.21813500
H	-6.17222300	-0.37006300	2.47626400
N	-5.20979800	-3.31336800	3.54655800
C	-5.36993200	-4.73894300	3.31841600
H	-4.36243400	-2.98964500	4.00101700
H	-5.35089500	-4.96421400	2.24713700
C	-8.91327200	-5.00413500	-0.26179600

C	-7.48962000	-4.69535300	-0.74945700
C	-7.19639200	-3.21105900	-1.02633500
C	-5.70496400	-2.99753600	-1.30711100
C	-8.03942900	-2.64909800	-2.17839200
H	-9.66360600	-4.68870000	-0.99608700
H	-6.78019600	-5.04348200	0.01145100
H	-7.27775000	-5.27781200	-1.65940100
H	-7.43829200	-2.65985000	-0.11144400
H	-5.49378800	-1.94087500	-1.49647900
H	-5.08829500	-3.29720500	-0.45600800
H	-5.38587900	-3.57107500	-2.18865500
H	-7.80385200	-1.59401100	-2.34758500
H	-7.83775100	-3.19559000	-3.10991700
H	-9.11381900	-2.71499000	-1.97775100
C	1.14891400	-6.10529400	2.94431700
O	0.18274100	-6.51671600	3.58221400
N	1.25793400	-4.79699000	2.54094100
C	0.08164900	-3.93796500	2.60184800
C	-0.87563700	-4.23589200	1.43916800
S	-2.54853600	-3.51377700	1.61510800
O	4.87350400	-3.43593200	-3.23456200
C	-1.39770200	-0.14998000	-0.55860200
C	-1.39297300	-1.30993100	0.31130100
C	-2.55513500	-1.87403100	0.81698500
O	-3.71625500	-1.35004500	0.84498900
C	-0.46251700	0.83466900	-0.49561700
C	-2.46244000	-0.08055800	-1.63643200
H	-3.44970200	-0.27213300	-1.21682400
H	-2.46704300	0.89207000	-2.13540900
H	-2.29141500	-0.85661300	-2.39115500
H	0.28218500	0.85808300	0.29015500
H	-0.45087000	-1.67111300	0.70640900
H	-0.42680100	1.62947800	-1.23370700
H	-1.04920500	-5.31458700	1.41326000
H	-0.43133600	-3.94281800	0.48453200
H	-0.43228900	-4.12607100	3.54584100
H	0.40242800	-2.89315800	2.58956700
H	1.95721800	-4.56974000	1.83885900
C	2.29395500	-7.01408700	2.53265900
H	2.22497400	-7.23256200	1.46013300
H	3.27343300	-6.55037700	2.68371400
H	2.22604600	-7.94440000	3.09590700
H	-0.61141500	-4.67683500	-6.37690600
H	1.12455200	-4.84624300	-5.87175900
H	5.72625300	-6.94771800	-2.80784200
H	8.28226200	-1.63126500	-4.40173200
H	9.78921900	-2.40239100	-3.72754400
H	8.39996200	0.18531800	-4.01516700
H	8.71021600	1.79959900	-4.78362900
H	0.75388100	2.41684900	7.44211500
H	2.52393600	2.41491400	7.60082600
H	6.96279400	6.53510500	-0.08460800
H	5.14965200	6.67815800	-0.03373900
H	3.56156100	9.26433400	-1.67676500
H	4.27890100	7.66829300	-1.34225600
H	3.39800000	8.64231900	3.09165100
H	4.42077000	7.91982300	4.40026000
H	-1.99915500	8.30636100	3.71644200
H	-2.32870600	7.33722600	2.25595100
H	-4.94747600	5.93711000	-4.96298400
H	-6.37452200	5.17561800	-4.16909500
H	-8.43102500	0.97036200	-5.91751400
H	-7.81888500	2.44414700	-6.76323300
H	-11.18369300	0.92476700	-4.31652400
H	-9.43702900	1.38703300	-4.41975600
H	-9.11477200	-4.46465400	0.66949300
H	-9.02960300	-6.08517400	-0.13187800
H	-4.55657700	-5.28772600	3.80455600
H	-6.33718300	-5.06328400	3.71638000
H	-3.48765700	-2.45649800	8.11656800
H	-1.72691600	-2.23476700	8.31755400
H	0.54197400	0.43448400	-6.52430600
H	0.78300400	2.11310600	-5.85576700
H	3.91495200	-3.42395600	-3.03360400
H	5.11843000	-2.50739200	-3.34891600
H	5.18354200	0.00866500	-4.11964200
O	-3.15942800	2.66202800	3.50455300
H	-3.31065300	2.18732400	2.65432400
H	-3.93624900	2.44911900	4.03731900
O	4.43232800	0.83854300	-5.28250800
H	4.53352200	1.78823000	-5.41828900
H	3.48457800	0.69342100	-5.03414700
O	-3.42302200	1.39608600	1.05780100
H	-3.60948400	0.42594000	1.00941100

TS2 (+13.1)

C	3.19760900	8.23171500	-1.58707300
C	2.33212000	7.63680700	-2.70655000
C	2.09012800	6.15371900	-2.57011300
C	1.15236800	5.65875400	-1.65532200
C	2.79925900	5.24309800	-3.36192000
C	0.91249700	4.28962600	-1.55701000
C	2.56567300	3.87157600	-3.26391900
C	1.61475200	3.39339200	-2.36319900
H	2.70105600	8.16123100	-0.61543000
H	1.36893100	8.16221300	-2.72792600
H	2.80998800	7.83130400	-3.67402900
H	0.61433900	6.35058700	-1.01398800
H	3.52828500	5.61935300	-4.07603400
H	0.17148000	3.91670300	-0.85806100
H	3.10290100	3.17839800	-3.90382300
H	1.42389100	2.33109500	-2.31010500
C	5.94175500	6.11075900	0.01423400
C	5.75489100	4.74938000	-0.65261300
C	4.56017500	3.99466200	-0.06850300
S	4.78223800	3.42864500	1.66473900
C	6.06156500	2.14418800	1.44866600
H	5.98809900	5.98045000	1.10052100
H	5.56649100	4.88008900	-1.72711200
H	6.66797600	4.14603900	-0.56932900
H	3.68730100	4.64997700	-0.02525200
H	4.29384700	3.13365500	-0.68163800
H	6.20086200	1.67133700	2.42294500
H	5.74920300	1.37647400	0.73689100
H	7.01531800	2.56988000	1.12723000
C	-1.96737300	7.28388700	3.27253100
C	-0.53897500	6.79138900	3.42211400
O	0.22054000	7.32853200	4.23817800
H	-2.62651100	6.63027200	3.85721800

N	-0.14419400	5.72909700	2.67330800
C	1.18600500	5.12881900	2.91039900
C	2.32893200	6.08173800	2.49051800
O	2.67247400	6.21244200	1.31617100
C	1.15946500	3.84199000	2.07560000
C	-0.32784000	3.48226500	1.98445900
C	-1.02394700	4.84459000	1.88492900
H	1.28163800	4.91244800	3.98143300
H	1.56374800	4.06712900	1.08650600
H	1.77516700	3.05841300	2.52007800
H	-0.54739300	2.84156400	1.12574300
H	-0.67957000	2.97196000	2.88522400
H	-1.08541700	5.19610900	0.84637400
H	-2.02830700	4.80646900	2.30995900
N	2.92812500	6.71679800	3.53189900
C	3.88828800	7.79395900	3.30383000
H	2.30012700	6.83892500	4.32040100
H	4.59751600	7.48267600	2.53671300
C	1.79305100	1.87884500	7.19730500
C	1.81072600	1.56751400	5.68370200
C	2.71731200	0.43035200	5.27449000
C	2.25439600	-0.89292600	5.33405200
C	4.02221200	0.66136700	4.82256400
C	3.06883300	-1.95699900	4.94777900
C	4.84503900	-0.40255700	4.44683300
C	4.37106600	-1.71338600	4.50704600
H	1.75944600	0.95989000	7.78977000
H	0.78812900	1.32012100	5.37583900
H	2.08345800	2.47504800	5.13244500
H	1.23820000	-1.08587900	5.66943700
H	4.38794600	1.68267600	4.75027500
H	2.68178700	-2.97168100	4.96209900
H	5.85501800	-0.20713000	4.09729700
H	5.00328600	-2.53818300	4.19779200
C	0.00761200	-4.83092800	-5.52506700
C	-0.23600600	-3.70099600	-4.54731200
O	-0.58992000	-2.58474500	-4.90913000
H	-0.18748800	-5.79611500	-5.04282500
N	-0.09018500	-4.06492900	-3.23452000
C	0.04746700	-3.05701700	-2.21522200
C	1.52230400	-2.89931400	-1.85944200
O	2.37292700	-3.58538400	-2.43553400
H	0.43485000	-4.91279600	-3.05555700
H	-0.33111800	-2.11534200	-2.61412900
H	-0.54362200	-3.30133500	-1.32963300
N	1.84356600	-2.02184800	-0.89062500
C	3.23217400	-1.75788300	-0.56234400
C	3.91754800	-3.01537500	-0.00824400
O	3.29883400	-3.94737000	0.51863900
C	3.37268400	-0.58964600	0.44195100
C	3.15908400	0.75390800	-0.25266600
C	2.47428800	-0.74999200	1.67248600
H	1.12436800	-1.39968600	-0.55408200
H	3.76096700	-1.48882500	-1.48324700
H	4.41047100	-0.62826000	0.79049600
H	3.16603700	1.56620900	0.47737200
H	3.94474800	0.94439300	-0.98661800
H	2.21494100	0.78718300	-0.79009800
H	2.76582700	-0.03358700	2.44217700
H	1.42196600	-0.56015000	1.44109500
H	2.55478600	-1.75066000	2.09952000
N	5.25850100	-2.97962300	-0.11483600
C	6.13327000	-4.01150700	0.44113300
C	5.76923300	-5.41244200	-0.09207100
O	5.80188100	-6.39787600	0.64078500
C	6.15057300	-3.98401900	1.96662500
H	5.68797600	-2.21525100	-0.62557000
H	7.12621700	-3.78646200	0.03889600
H	6.79476200	-4.77917300	2.34397100
H	6.51701200	-3.01484400	2.31773900
H	5.14325300	-4.15027600	2.35324500
N	5.50090600	-5.44651700	-1.42013600
C	5.07016700	-6.66309600	-2.07652300
C	3.62103200	-6.55830800	-2.56993600
O	2.68629200	-6.36436200	-1.52075000
H	5.47030500	-4.58278200	-1.96461300
H	5.14623700	-7.46191900	-1.33521000
H	3.54453400	-5.75722000	-3.31880300
H	3.34662900	-7.50014100	-3.05996300
H	2.90472800	-5.52422300	-1.08515800
C	0.04516300	1.14406000	-5.82970800
C	-0.18853500	0.60524800	-4.42715000
S	1.28094900	-0.01358800	-3.50935500
H	-0.95001800	1.42668600	-6.20739800
H	-0.63605300	1.39221000	-3.81324600
H	-0.87941100	-0.23828300	-4.48422400
H	0.57509200	0.24720000	-2.17864600
C	8.74037700	-2.50030500	-4.39606400
C	7.98791100	-3.44224000	-3.45368200
O	7.96077000	-2.95403600	-2.11371800
H	8.89659300	-2.98300400	-5.36884300
H	6.97964200	-3.65317100	-3.82184400
H	8.52094600	-4.39783400	-3.40139200
H	7.45439800	-2.12650300	-2.08636800
C	7.86545600	1.22567300	-4.45321400
C	7.48578800	1.97005900	-3.15720800
C	6.11731000	1.58520900	-2.55664100
C	5.99870200	0.08951400	-2.40443400
O	6.52130800	-0.52555700	-1.48129900
O	5.35154500	-0.57196600	-3.36220500
H	6.99367500	1.07629700	-5.09876200
H	8.26061600	1.79918000	-2.40246100
H	7.45582300	3.04981600	-3.33649500
H	6.02314700	2.03133300	-1.56700400
H	5.31413300	1.96823400	-3.18878400
C	-5.44441700	5.14818300	-4.10025600
C	-4.87371200	5.57920600	-2.73498300
C	-3.35812200	5.29212200	-2.62447600
C	-5.65828800	4.95603700	-1.57445400
C	-2.97156800	3.81553200	-2.46142800
H	-5.07815700	4.15171900	-4.37506600
H	-4.99285600	6.66966900	-2.66052800
H	-2.85635200	5.70034100	-3.51172000
H	-2.95829500	5.85099100	-1.76781100
H	-5.22897400	5.23629400	-0.60618100
H	-6.70305300	5.28389300	-1.58678200
H	-5.66234400	3.86478900	-1.62465700
H	-1.88282000	3.70540600	-2.49308400
H	-3.31661000	3.40750200	-1.50579000
H	-3.38686500	3.18883200	-3.25808800

C	-7.78822300	1.25598900	-6.36452300
C	-6.44772300	1.15463500	-5.62070800
C	-6.20994400	-0.18815700	-4.90887300
C	-6.32522700	-1.38807600	-5.85597900
C	-4.84894900	-0.19014900	-4.20714000
H	-7.76997900	0.69090600	-7.30217700
H	-5.62014500	1.34690600	-6.31861600
H	-6.39546900	1.94953000	-4.86495300
H	-6.98552900	-0.28963100	-4.13649400
H	-6.14116700	-2.32295100	-5.31691000
H	-7.31747200	-1.45801800	-6.31213500
H	-5.58619400	-1.31765900	-6.66405000
H	-4.71456600	-1.09367500	-3.60615400
H	-4.03074100	-0.14974800	-4.93706400
H	-4.74517500	0.67034500	-3.53989400
C	-10.33620800	0.84621000	-3.56353900
C	-10.32786900	1.60142900	-2.22335300
C	-9.06110400	1.25751700	-1.45959600
C	-9.09564900	0.38292900	-0.36640700
C	-7.81075600	1.71268200	-1.90285500
C	-7.92208900	-0.04714700	0.25810100
C	-6.63524100	1.28215500	-1.28693700
C	-6.68425300	0.39282800	-0.21216200
H	-10.03525600	-0.19048600	-3.36198700
H	-10.39656100	2.68301400	-2.39474600
H	-11.20036500	1.31898200	-1.62417900
H	-10.05561000	0.01441600	-0.01306100
H	-7.75637100	2.39924700	-2.74448700
H	-7.96452000	-0.74654700	1.08837900
H	-5.67414500	1.63099500	-1.65002100
H	-5.76489200	0.03485100	0.23767100
C	-2.30922900	-2.67975300	7.74425400
C	-2.31597700	-2.14239000	6.32112600
O	-2.66370100	-2.83807900	5.37009400
H	-2.16987300	-3.76019800	7.71079100
N	-1.93690100	-0.83466800	6.18654300
C	-2.23253000	-0.05625400	4.96976400
C	-3.75940200	-0.10484600	4.73682500
O	-4.50754200	0.40607700	5.57469300
C	-1.34760800	-0.44760300	3.79263600
H	-1.89804400	-0.29840200	7.04088800
H	-2.03707900	0.98801200	5.21774900
H	-1.60225900	0.16558300	2.92581800
H	-0.29690000	-0.27397200	4.03681900
H	-1.46828800	-1.50076300	3.54034400
N	-4.19606900	-0.73929400	3.63019300
C	-5.59257500	-0.99159600	3.34978300
C	-5.92041100	-2.47134600	3.11296300
O	-6.93826000	-2.78110900	2.48874700
H	-3.54142000	-1.08795600	2.94589600
H	-6.16855400	-0.62445700	4.20315700
H	-5.92008700	-0.45037400	2.45995400
N	-5.07132000	-3.36803300	3.65857400
C	-5.24676100	-4.79682700	3.45066200
H	-4.23475800	-3.04947300	4.13687600
H	-5.25209600	-5.03010600	2.38105200
C	-8.86563300	-5.12346000	-0.04759800
C	-7.46176300	-4.79506800	-0.57607800
C	-7.20783000	-3.30888000	-0.88077700
C	-5.72572100	-3.06642600	-1.18721100

C	-8.07967800	-2.78242700	-2.02806000
H	-9.63970700	-4.82319000	-0.76342400
H	-6.72588900	-5.11809300	0.17076300
H	-7.26143700	-5.38583600	-1.48289400
H	-7.44959500	-2.74872300	0.02844900
H	-5.54321000	-2.00929800	-1.40259000
H	-5.09171800	-3.33630000	-0.33834000
H	-5.40391800	-3.65026000	-2.06030500
H	-7.87696200	-1.72222800	-2.20795200
H	-7.87303700	-3.33000900	-2.95748100
H	-9.14918600	-2.87829000	-1.81374400
C	1.29260100	-6.08564400	2.96223000
O	0.30271500	-6.51950600	3.54608100
N	1.43713600	-4.75232500	2.65677600
C	0.26473900	-3.89273400	2.73388900
C	-0.61042000	-4.07347300	1.48484900
S	-2.32447300	-3.44705600	1.65801700
O	4.86533000	-3.41778000	-3.39283300
C	-1.40876100	-0.16839600	-0.67253000
C	-1.31543600	-1.17860900	0.29992600
C	-2.44739400	-1.84885000	0.82889600
O	-3.61900900	-1.39158000	0.84436000
C	-0.37241500	0.74499500	-0.94585400
C	-2.63271900	-0.08650200	-1.56405800
H	-3.27435900	0.74631100	-1.26210300
H	-2.31810800	0.10009300	-2.59294900
H	-3.23433300	-0.99224200	-1.53409900
H	-0.64851900	1.63152200	-1.51317100
H	-0.36408400	-1.37692100	0.78002500
H	0.36170300	0.92781600	-0.16648200
H	-0.72885100	-5.14357100	1.29969300
H	-0.13207300	-3.62930800	0.61127800
H	-0.30234900	-4.17078600	3.62355100
H	0.58438800	-2.85275600	2.84029000
H	2.14540900	-4.49689600	1.97197900
C	2.42414200	-6.98632600	2.50562100
H	2.30694000	-7.20201000	1.43619700
H	3.40773500	-6.52045400	2.61447600
H	2.38187000	-7.92092200	3.06418900
H	-0.70711500	-4.73335300	-6.34891100
H	1.04160500	-4.87952000	-5.88190300
H	5.73522300	-6.90970000	-2.91074500
H	8.19201500	-1.57265400	-4.59016300
H	9.72256200	-2.32225800	-3.94568200
H	8.29646200	0.24737600	-4.21512100
H	8.57067200	1.86073900	-4.99951900
H	0.88256000	2.45332800	7.39780700
H	2.65561800	2.47297500	7.51691200
H	6.87252900	6.60179000	-0.28796400
H	5.05926600	6.72378500	-0.19709000
H	3.40438700	9.28195100	-1.81813300
H	4.14798600	7.69642000	-1.49124500
H	3.35484700	8.68481700	2.95600700
H	4.41509000	7.98174700	4.24522700
H	-2.02264100	8.28912300	3.70341300
H	-2.37323000	7.30802900	2.25587000
H	-5.13639800	5.83744000	-4.89450200
H	-6.53574500	5.06392300	-4.06549500
H	-8.58085500	0.82436800	-5.74455900
H	-8.00527800	2.30032900	-6.61177500

H	-11.29627900	0.75550000	-4.08213800
H	-9.55780300	1.23745900	-4.22687200
H	-9.05283300	-4.58141000	0.88493900
H	-8.96626900	-6.20530300	0.09057200
H	-4.41617800	-5.33329900	3.92152900
H	-6.20082100	-5.13035000	3.87206300
H	-3.28481500	-2.46559400	8.19335600
H	-1.52275500	-2.22212700	8.35350700
H	0.38224300	0.39020700	-6.54981000
H	0.61823500	2.07527800	-5.89580300
H	3.92951900	-3.43761500	-3.10790000
H	5.09434700	-2.47752000	-3.40660800
H	4.83142700	0.03091800	-3.99051700
O	3.87924600	0.95989700	-4.82832700
H	3.71156300	0.63474300	-5.72340000
H	3.00612800	0.83362000	-4.35638400
O	-3.06512000	2.70020400	3.66989600
H	-3.24574800	2.21941800	2.83407600
H	-3.78268900	2.43507000	4.26125500
O	-3.30220200	1.38785500	1.21210900
H	-3.51788000	0.43353400	1.10795100

E:P (-2.0)

C	3.24609900	8.21254200	-1.58924600
C	2.40265900	7.59725300	-2.71692000
C	2.18614500	6.10552300	-2.59700400
C	1.28170200	5.57861800	-1.66609400
C	2.87262200	5.21688500	-3.43451000
C	1.05054800	4.20466900	-1.60068700
C	2.64477100	3.84151600	-3.36956100
C	1.72283000	3.33000600	-2.45629700
H	2.73647900	8.14824400	-0.62392200
H	1.42873200	8.10269600	-2.74859000
H	2.88570000	7.80482400	-3.67906000
H	0.76438500	6.24804100	-0.98493500
H	3.57651500	5.61491500	-4.16215000
H	0.33595900	3.81554400	-0.88218800
H	3.15726900	3.16107300	-4.04343500
H	1.54952000	2.25819700	-2.44791700
C	5.96422300	6.09286800	0.05687900
C	5.77689500	4.73284700	-0.61163800
C	4.57248300	3.99068000	-0.03396900
S	4.78025900	3.43392000	1.70447900
C	6.05684000	2.14432000	1.49825700
H	5.99708700	5.96298400	1.14381400
H	5.59528600	4.86367500	-1.68701900
H	6.68383400	4.12146700	-0.52261900
H	3.70328700	4.65131200	-0.00355300
H	4.30613200	3.12791900	-0.64312200
H	6.16708100	1.64956200	2.46543300
H	5.75970600	1.39607900	0.75997900
H	7.02085900	2.57128800	1.21178600
C	-1.98271900	7.30433500	3.20935000
C	-0.55935900	6.80891800	3.38661900
O	0.18686600	7.34444900	4.21499500
H	-2.65306200	6.65801100	3.78971700
N	-0.15612000	5.74192000	2.64955800
C	1.16762600	5.13793600	2.91022900
C	2.31622300	6.08607900	2.49520100
O	2.66440700	6.21233800	1.32267800
C	1.14530900	3.84349300	2.08633800
C	-0.34427700	3.49508500	1.97942800
C	-1.02298900	4.86261100	1.84492100
H	1.24771600	4.93101400	3.98436500
H	1.56333200	4.05680300	1.10031100
H	1.75019100	3.06026200	2.54570100
H	-0.56027400	2.84503900	1.12739000
H	-0.71495200	3.00256800	2.88282900
H	-1.04867200	5.20135000	0.80058100
H	-2.04025000	4.84251500	2.23967100
N	2.90883600	6.72466900	3.53791900
C	3.87359100	7.79864100	3.31225100
H	2.27890900	6.84926100	4.32397600
H	4.59113100	7.48049100	2.55582700
C	1.71261900	1.91017100	7.21011100
C	1.75013000	1.59006300	5.69908200
C	2.64848900	0.44041500	5.30632700
C	2.17234300	-0.87768000	5.37461500
C	3.95478600	0.65549300	4.85141500
C	2.97399700	-1.95183100	4.98937300
C	4.76376800	-0.41838300	4.47416000
C	4.27560600	-1.72361100	4.53835300
H	1.66933300	0.99473800	7.80750800
H	0.72904300	1.35271400	5.37682500
H	2.04071900	2.49125800	5.14673100
H	1.15512400	-1.05823000	5.71506700
H	4.33084500	1.67238600	4.77175100
H	2.57727100	-2.96277400	5.00986000
H	5.77293900	-0.23482900	4.11665600
H	4.89547300	-2.55479000	4.22127900
C	0.07021200	-4.86145600	-5.49842000
C	-0.21531900	-3.73016500	-4.53066500
O	-0.77040700	-2.69252500	-4.87081500
H	-0.13056300	-5.82150700	-5.00757100
N	0.13270500	-4.00821700	-3.23741200
C	0.21372500	-2.95469800	-2.26025900
C	1.62456400	-2.90887600	-1.69972400
O	2.46434800	-3.75450200	-2.03456300
H	0.75530200	-4.78966400	-3.06632200
H	0.00746300	-2.00254600	-2.75668700
H	-0.51214400	-3.08776200	-1.44955600
N	1.89962300	-1.94566000	-0.80033900
C	3.28168300	-1.68224800	-0.42250700
C	3.94302400	-2.92166000	0.18679700
O	3.33001600	-3.75799400	0.85773400
C	3.35351800	-0.49403100	0.56225500
C	3.13043100	0.82092700	-0.18396300
C	2.40015000	-0.65171500	1.74936300
H	1.29478100	-1.13755800	-0.83812900
H	3.84203100	-1.41877800	-1.32554200
H	4.37578400	-0.49564500	0.95683500
H	3.04041900	1.65040400	0.51956600
H	3.96567400	1.02613400	-0.85463900
H	2.24657100	0.79538100	-0.81972500
H	2.61805000	0.10252200	2.50719600
H	1.35789400	-0.51880700	1.44510900
H	2.49644900	-1.63468500	2.21030500
N	5.27136700	-2.96243800	-0.02790100
C	6.14315800	-4.00401600	0.51133400

C	5.74715100	-5.40527200	-0.00139700
O	5.77294000	-6.38349200	0.74201000
C	6.22327700	-3.96760000	2.03500500
H	5.68650500	-2.25883200	-0.63060500
H	7.12493000	-3.79666800	0.07344800
H	6.89218600	-4.75344100	2.38851600
H	6.59720700	-2.99284200	2.36293900
H	5.23584800	-4.14045900	2.46521500
N	5.47204700	-5.45463200	-1.32936500
C	5.08344600	-6.68959200	-1.97626400
C	3.64553800	-6.65238300	-2.50712100
O	2.67531600	-6.47299500	-1.48925400
H	5.44379600	-4.59854000	-1.88454300
H	5.16782500	-7.47383200	-1.22030500
H	3.55801600	-5.87050600	-3.27576600
H	3.42742700	-7.61452600	-2.98652000
H	2.76015100	-5.55301900	-1.19229700
C	0.13149100	1.10965600	-5.83167000
C	0.07166300	0.54771200	-4.41317700
S	1.66137200	-0.03714200	-3.67583000
H	-0.89260400	1.37091200	-6.14125300
H	-0.35672700	1.31791700	-3.75830700
H	-0.60645700	-0.31122900	-4.41458700
H	0.23028600	0.46885500	-1.73641800
C	8.79411300	-2.54906100	-4.27104000
C	8.01104200	-3.45789800	-3.32030900
O	7.97288200	-2.93438700	-1.99414100
H	8.95568600	-3.04833500	-5.23427200
H	7.00368700	-3.65626700	-3.69915000
H	8.52257200	-4.42317000	-3.23648200
H	7.45929300	-2.10928600	-1.99227100
C	7.92895400	1.17914500	-4.35977500
C	7.47290100	1.92113800	-3.08934100
C	6.06555900	1.52545800	-2.59512900
C	5.96200400	0.02686400	-2.44465600
O	6.42790600	-0.56104200	-1.46812100
O	5.41493600	-0.65181000	-3.43914300
H	7.08329300	1.01682800	-5.03673000
H	8.19260600	1.74757900	-2.28217300
H	7.45466100	3.00138300	-3.26796200
H	5.88981800	1.97668000	-1.61873600
H	5.30932700	1.89035100	-3.29218700
C	-5.37243500	5.14090400	-4.19498000
C	-4.82317900	5.57274000	-2.82244700
C	-3.30142900	5.32564700	-2.70004800
C	-5.60174000	4.91052700	-1.67941600
C	-2.87481900	3.85870000	-2.54828500
H	-5.00316000	4.14237700	-4.45882800
H	-4.97316800	6.65836700	-2.73509100
H	-2.80149700	5.75504700	-3.57780200
H	-2.92562500	5.88656800	-1.83393200
H	-5.18690600	5.18012100	-0.70181400
H	-6.65411400	5.21273300	-1.69310900
H	-5.57886400	3.82049000	-1.75362500
H	-1.78288700	3.78013300	-2.55741400
H	-3.23539800	3.43161800	-1.60561400
H	-3.25301700	3.23119500	-3.36223600
C	-7.69753400	1.24126900	-6.46884000
C	-6.37463100	1.14623200	-5.69351400
C	-6.15548700	-0.19286800	-4.96948300
C	-6.21227000	-1.39363400	-5.92061600
C	-4.83063200	-0.17729000	-4.20261300
H	-7.66133600	0.66980000	-7.40171300
H	-5.52921900	1.33621800	-6.36977700
H	-6.34389800	1.94526700	-4.94059400
H	-6.96641300	-0.30294500	-4.23478200
H	-6.04907300	-2.32763200	-5.37327800
H	-7.17916100	-1.47006200	-6.42750500
H	-5.43275300	-1.31772800	-6.68872100
H	-4.70680700	-1.08899700	-3.61157600
H	-3.97749200	-0.10650900	-4.88803300
H	-4.78340600	0.67486700	-3.51775200
C	-10.28186100	0.85330300	-3.69837900
C	-10.30789600	1.61922800	-2.36430100
C	-9.08596000	1.25667100	-1.53983200
C	-9.19271300	0.38639800	-0.44767400
C	-7.80785300	1.69530400	-1.91575400
C	-8.06133500	-0.05405200	0.24303900
C	-6.67387500	1.25759600	-1.23109400
C	-6.79598500	0.37468400	-0.15743300
H	-9.98720000	-0.18236500	-3.48369700
H	-10.34515400	2.70037300	-2.54678700
H	-11.21150700	1.36220700	-1.80082700
H	-10.17570400	0.03192300	-0.14753900
H	-7.69923800	2.37755400	-2.75546000
H	-8.15415700	-0.74989000	1.07199600
H	-5.68936700	1.59856400	-1.53341300
H	-5.90923900	0.01294100	0.34906400
C	-2.40908000	-2.63433500	7.72895800
C	-2.39966000	-2.09370200	6.30825000
O	-2.73326000	-2.78371400	5.34632600
H	-2.27156700	-3.71498500	7.69861900
N	-2.02793400	-0.78361100	6.18604600
C	-2.32320300	0.00475900	4.97762600
C	-3.85230600	-0.02728300	4.75536900
O	-4.59338000	0.51113300	5.57976500
C	-1.45444600	-0.39180500	3.78938200
H	-1.98176000	-0.25484300	7.04469300
H	-2.11116700	1.04488900	5.22716900
H	-1.71435500	0.23395100	2.93288800
H	-0.39867400	-0.23329100	4.02154500
H	-1.58765600	-1.44481900	3.53876300
N	-4.29716400	-0.69031500	3.66748800
C	-5.69367900	-0.96171900	3.40100200
C	-5.98783800	-2.43892500	3.10811000
O	-6.99527300	-2.74850100	2.46959400
H	-3.63651300	-1.07976600	3.01507100
H	-6.26012700	-0.64421300	4.28071000
H	-6.05738400	-0.38907000	2.54473500
N	-5.11727700	-3.33722300	3.61960700
C	-5.29757800	-4.76611600	3.40959700
H	-4.31283200	-3.02463200	4.15384600
H	-5.29122400	-5.00100000	2.34061200
C	-8.87285500	-5.10190100	-0.13278900
C	-7.46400800	-4.77410700	-0.64662100
C	-7.20765700	-3.28492200	-0.93459800
C	-5.72500100	-3.04037600	-1.23604300
C	-8.07446500	-2.74538200	-2.07949900
H	-9.63822900	-4.80364500	-0.85849600
H	-6.73446200	-5.10615900	0.10294500

H	-7.25727700	-5.35630100	-1.55718800
H	-7.45099000	-2.73255200	-0.02060700
H	-5.54272700	-1.97755400	-1.42292500
H	-5.09318500	-3.34163500	-0.39545900
H	-5.40456500	-3.59917700	-2.12519000
H	-7.86414200	-1.68599500	-2.25445400
H	-7.87152700	-3.29005600	-3.01114900
H	-9.14422300	-2.83417300	-1.86510400
C	1.18497000	-6.10470500	2.90086000
O	0.11809900	-6.56329600	3.30408900
N	1.36576100	-4.75692500	2.69710200
C	0.19984500	-3.89397000	2.71671300
C	-0.55503300	-3.97313700	1.37688300
S	-2.33391700	-3.52730100	1.50879100
O	4.83055000	-3.46338900	-3.35470900
C	-1.50553400	-0.20586900	-0.62568800
C	-1.41765000	-1.07058200	0.41165300
C	-2.54517100	-1.88899500	0.87639300
O	-3.70107900	-1.44074700	0.91770200
C	-0.41219000	0.78008000	-0.89055000
C	-2.63415100	-0.18812400	-1.61551900
H	-3.14162900	0.78216800	-1.59748800
H	-2.22019500	-0.30666400	-2.62280400
H	-3.37492100	-0.96431400	-1.43929700
H	-0.83799800	1.74307500	-1.19330500
H	-0.51056700	-1.11652900	1.00431700
H	0.22000900	0.93774500	-0.01540400
H	-0.57247600	-5.01293400	1.04449200
H	-0.06616800	-3.37596900	0.60718900
H	-0.45300200	-4.22367600	3.52687100
H	0.51371500	-2.86660000	2.92098200
H	2.15296800	-4.45020000	2.12809900
C	2.37852400	-6.98140300	2.57362300
H	2.42159400	-7.14681300	1.48992200
H	3.32881200	-6.51642700	2.84972000
H	2.26215300	-7.94110700	3.07674300
H	-0.63387100	-4.76728100	-6.33156300
H	1.10854200	-4.91550300	-5.84184400
H	5.75829700	-6.94245100	-2.80050300
H	8.25159200	-1.62104000	-4.47763900
H	9.77089000	-2.37124800	-3.80835700
H	8.35483200	0.20063500	-4.10893200
H	8.64520400	1.80880000	-4.89866500
H	0.80099600	2.48800300	7.39620600
H	2.57247600	2.50373700	7.53761200
H	6.89976900	6.57986500	-0.23415500
H	5.08506900	6.70622000	-0.16733300
H	3.45851000	9.26096900	-1.82293900
H	4.19377900	7.67550500	-1.47795400
H	3.34673900	8.68928100	2.95334100
H	4.38845100	7.99032900	4.25965900
H	-2.04082500	8.31212000	3.63438900
H	-2.37581400	7.32434000	2.18769900
H	-5.05137200	5.82260400	-4.98997400
H	-6.46384900	5.05710000	-4.17410300
H	-8.49919100	0.81501300	-5.85682100
H	-7.90851700	2.28490300	-6.72430100
H	-11.23574700	0.76228100	-4.22865300
H	-9.49432600	1.23892800	-4.35386100
H	-9.07015300	-4.55412400	0.79461600
H	-8.97813300	-6.18241000	0.00993600
H	-4.47465400	-5.30249500	3.89380000
H	-6.25797100	-5.09507800	3.82044200
H	-3.38962700	-2.41531700	8.16446000
H	-1.62906200	-2.17569100	8.34569300
H	0.47041100	0.35312100	-6.54738500
H	0.70369000	2.04114400	-5.89883000
H	3.91843200	-3.50472000	-3.01509500
H	5.03297900	-2.51562800	-3.40392400
H	4.93270000	-0.05695500	-4.14698100
O	4.14564800	0.78396600	-5.07500500
H	4.07090200	0.36064300	-5.94156600
H	3.20518600	0.64387100	-4.65783700
O	-3.16898000	2.68698800	3.58551100
H	-3.44144300	2.23531100	2.76050900
H	-3.87814300	2.49274500	4.21335600
O	-3.58921000	1.43085700	1.10501100
H	-3.70980700	0.46162900	1.04301700

TS (Cys56$_B$-SH) (-3.4)

C	3.24583300	8.20790500	-1.62988200
C	2.40329600	7.58184300	-2.75325400
C	2.19020900	6.08946000	-2.62422100
C	1.27733400	5.56629800	-1.69920900
C	2.88749600	5.19594200	-3.44772800
C	1.04882500	4.19229400	-1.62616900
C	2.66372900	3.81997600	-3.37487700
C	1.73319300	3.31318600	-2.46770500
H	2.73671400	8.14803200	-0.66400500
H	1.42762700	8.08357200	-2.78846700
H	2.88553600	7.78395500	-3.71692800
H	0.75166900	6.23892000	-1.02775600
H	3.59940700	5.58969300	-4.16967200
H	0.32884000	3.80591400	-0.91170000
H	3.19006400	3.13688900	-4.03515900
H	1.56145700	2.24219900	-2.44418700
C	5.96463500	6.09641500	0.02561100
C	5.78200200	4.73235200	-0.63670000
C	4.57884500	3.98633200	-0.06111400
S	4.78475100	3.42756800	1.67716500
C	6.05857200	2.13458400	1.47077600
H	5.99751900	5.97179100	1.11328800
H	5.60443600	4.85732900	-1.71338300
H	6.69020100	4.12419700	-0.54272900
H	3.70695400	4.64356700	-0.03231200
H	4.31964200	3.12198600	-0.67106500
H	6.16604400	1.63896900	2.43781800
H	5.76303700	1.38794400	0.73032300
H	7.02369300	2.56040200	1.18671100
C	-1.98203600	7.32091200	3.17367200
C	-0.55844800	6.82698600	3.35314700
O	0.18805200	7.36691900	4.17817600
H	-2.65215700	6.67717800	3.75717500
N	-0.15555200	5.75622100	2.62130300
C	1.16893800	5.15407500	2.88277300
C	2.31611600	6.10166200	2.46259800
O	2.66042200	6.22540000	1.28874200
C	1.14632900	3.85680700	2.06345100
C	-0.34326000	3.50662700	1.96255800

C	-1.02345600	4.87281800	1.82292200
H	1.25036400	4.95078900	3.95750800
H	1.56121100	4.06774300	1.07555100
H	1.75412900	3.07607600	2.52316600
H	-0.56192600	2.85238800	1.11449500
H	-0.71113400	3.01859100	2.86956300
H	-1.05161100	5.20635500	0.77685700
H	-2.03993100	4.85379500	2.21977300
N	2.91067800	6.74272600	3.50271100
C	3.87418900	7.81684700	3.27335700
H	2.28292100	6.86802100	4.29025500
H	4.59242700	7.49648900	2.51845800
C	1.71500200	1.94610300	7.19885900
C	1.75261200	1.61954800	5.68921800
C	2.65491600	0.47155900	5.30051100
C	2.18413000	-0.84814500	5.37464800
C	3.96042300	0.68980500	4.84469900
C	2.99048100	-1.92089700	4.99508900
C	4.77394100	-0.38254500	4.47293000
C	4.29134400	-1.68951400	4.54353400
H	1.67223100	1.03325800	7.80024400
H	0.73190000	1.37757500	5.36914900
H	2.03954700	2.51949100	5.13291500
H	1.16765000	-1.03131000	5.71603200
H	4.33243100	1.70774000	4.75984400
H	2.59809500	-2.93341300	5.02060500
H	5.78221300	-0.19663700	4.11436900
H	4.91483800	-2.51943800	4.23011900
C	0.07183000	-4.88467500	-5.47792300
C	-0.21584900	-3.75275800	-4.51099700
O	-0.74190000	-2.70215000	-4.85850200
H	-0.12838300	-5.84411300	-4.98584500
N	0.09035600	-4.04678900	-3.20954000
C	0.17359000	-2.99847800	-2.22633300
C	1.60085700	-2.92425600	-1.70399500
O	2.44821700	-3.73891500	-2.08635600
H	0.71142600	-4.82939200	-3.03616200
H	-0.07711900	-2.05266700	-2.71164600
H	-0.52783600	-3.15814700	-1.39986500
N	1.87988700	-1.97841800	-0.78763900
C	3.26603100	-1.69702100	-0.43654000
C	3.94549400	-2.92969600	0.16796000
O	3.33828100	-3.78447000	0.82454100
C	3.34916300	-0.50297500	0.53993300
C	3.11364100	0.80898400	-0.20746700
C	2.41672700	-0.65745900	1.74417900
H	1.23638500	-1.20311500	-0.73859700
H	3.80900600	-1.43554500	-1.35039700
H	4.37756700	-0.50113000	0.91680800
H	3.03919300	1.64228100	0.49377100
H	3.93450000	1.00866800	-0.89709100
H	2.21448900	0.77941900	-0.82056200
H	2.64603000	0.09913700	2.49623900
H	1.36800200	-0.52583700	1.45990500
H	2.52150000	-1.63906200	2.20653500
N	5.27288300	-2.94653800	-0.03968100
C	6.15440300	-3.97813000	0.50221800
C	5.76104200	-5.38593100	0.00704100
O	5.79544100	-6.35707000	0.75990500
C	6.24175100	-3.92605600	2.02527800
H	5.68223900	-2.22874700	-0.63548100
H	7.13186300	-3.76959800	0.05520900
H	6.91305800	-4.70725000	2.38465100
H	6.61551100	-2.94732800	2.34139200
H	5.25614100	-4.09591800	2.46166600
N	5.47485500	-5.45053800	-1.31805500
C	5.08600600	-6.69552100	-1.94812000
C	3.64613700	-6.65433900	-2.47283500
O	2.68345500	-6.45572300	-1.45019900
H	5.44500300	-4.59937600	-1.88334300
H	5.17305100	-7.47089500	-1.18356500
H	3.56108600	-5.87751900	-3.24700900
H	3.41461800	-7.61884700	-2.94081700
H	2.80483900	-5.54587400	-1.13516000
C	0.13277100	1.08467700	-5.83868000
C	0.06700700	0.53076100	-4.41997400
S	1.63362300	-0.11281600	-3.68752600
H	-0.89049900	1.34583200	-6.14854400
H	-0.33155800	1.31173600	-3.76087700
H	-0.62805400	-0.31366700	-4.41129500
H	0.21487800	0.46736500	-1.71770200
C	8.79545600	-2.56495500	-4.26266300
C	8.01536300	-3.46165900	-3.29789200
O	7.98372100	-2.92003500	-1.98011400
H	8.95457600	-3.07362000	-5.22154800
H	7.00688900	-3.66330000	-3.67120100
H	8.52770700	-4.42627000	-3.20515800
H	7.45347200	-2.10260700	-1.97963900
C	7.92938300	1.16270400	-4.36878000
C	7.48719800	1.89539900	-3.08709800
C	6.08408500	1.50412300	-2.58288100
C	5.94155900	-0.00239100	-2.45759600
O	6.41981900	-0.58901300	-1.47049900
O	5.36004600	-0.63371500	-3.43310600
H	7.07704800	0.99655000	-5.03612300
H	8.21423300	1.70497800	-2.29007800
H	7.48039100	2.97854200	-3.25261100
H	5.92745900	1.94625800	-1.59855300
H	5.32580700	1.89784900	-3.26279000
C	-5.37207600	5.11849100	-4.21955400
C	-4.81925700	5.54835200	-2.84827500
C	-3.29726700	5.30088500	-2.73001600
C	-5.59506400	4.88427900	-1.70449900
C	-2.87072900	3.83357400	-2.58141100
H	-5.00693400	4.11916400	-4.48578500
H	-4.96875500	6.63381800	-2.75898000
H	-2.79934900	5.73158000	-3.60826300
H	-2.91925900	5.86016100	-1.86385400
H	-5.17800300	5.15202000	-0.72736800
H	-6.64747500	5.18630000	-1.71522100
H	-5.57218900	3.79441900	-1.78097500
H	-1.77872500	3.75520100	-2.59187300
H	-3.23004400	3.40514000	-1.63890700
H	-3.25059900	3.20748300	-3.39572700
C	-7.69714400	1.21192000	-6.47538900
C	-6.37463000	1.12230300	-5.69858000
C	-6.15539900	-0.21161400	-4.96508900
C	-6.21115500	-1.41905400	-5.90776500
C	-4.83102100	-0.19015800	-4.19738500
H	-7.66013600	0.63580300	-7.40530200

H	-5.52887200	1.30799000	-6.37567300
H	-6.34461800	1.92650300	-4.95114500
H	-6.96653500	-0.31689000	-4.23001100
H	-6.04872900	-2.34925600	-5.35378000
H	-7.17754000	-1.49882100	-6.41502700
H	-5.43098800	-1.34856300	-6.67574800
H	-4.70732800	-1.09776600	-3.60002300
H	-3.97779100	-0.12395500	-4.88330700
H	-4.78425900	0.66682500	-3.51846600
C	-10.28112400	0.83593600	-3.70310700
C	-10.30792500	1.60650000	-2.37160400
C	-9.08836100	1.24511100	-1.54313400
C	-9.19926300	0.38083400	-0.44659200
C	-7.80856400	1.67934800	-1.91838400
C	-8.07032700	-0.05813000	0.24900700
C	-6.67702800	1.24307700	-1.22875600
C	-6.80329400	0.36600900	-0.15090600
H	-9.98661200	-0.19899400	-3.48458700
H	-10.34317100	2.68705200	-2.55788800
H	-11.21301700	1.35295000	-1.80897500
H	-10.18365400	0.03012500	-0.14666800
H	-7.69679000	2.35737300	-2.76109100
H	-8.16585300	-0.74909300	1.08168400
H	-5.69125300	1.58136800	-1.52989200
H	-5.91859000	0.00483400	0.35952400
C	-2.40577200	-2.59673200	7.73936400
C	-2.39698100	-2.05837100	6.31774700
O	-2.72487900	-2.75100600	5.35554200
H	-2.26761900	-3.67728900	7.71174100
N	-2.03330700	-0.74626600	6.19606800
C	-2.32175500	0.04234300	4.98647700
C	-3.84987800	0.01676600	4.75739900
O	-4.59214600	0.58490700	5.56063900
C	-1.45087500	-0.35813100	3.80097900
H	-1.98344300	-0.21771300	7.05457200
H	-2.10674300	1.08167800	5.23634600
H	-1.71290300	0.26301200	2.94188200
H	-0.39566700	-0.19602000	4.03301700
H	-1.58062700	-1.41287800	3.55566600
N	-4.29275000	-0.67509200	3.68700700
C	-5.68918800	-0.94404300	3.41680300
C	-5.98324200	-2.42126600	3.12458100
O	-6.98891900	-2.73107300	2.48366300
H	-3.62922200	-1.08770700	3.05228200
H	-6.25828800	-0.62418500	4.29402200
H	-6.05045000	-0.37215900	2.55884400
N	-5.11364100	-3.31957000	3.63806600
C	-5.29445700	-4.74894500	3.43031500
H	-4.31289900	-3.00752900	4.17824600
H	-5.28791500	-4.98610500	2.36196800
C	-8.87035900	-5.10147200	-0.10977000
C	-7.46129900	-4.77634500	-0.62470500
C	-7.20442700	-3.28846500	-0.91850800
C	-5.72148400	-3.04528000	-1.21968300
C	-8.07020200	-2.75331500	-2.06622500
H	-9.63565100	-4.80660600	-0.83689600
H	-6.73206300	-5.10574200	0.12636000
H	-7.25456900	-5.36232800	-1.53282000
H	-7.44818300	-2.73230400	-0.00697900
H	-5.53945400	-1.98313300	-1.41077300
H	-5.09052000	-3.34291900	-0.37712700
H	-5.40021600	-3.60777700	-2.10621200
H	-7.85996000	-1.69454500	-2.24497800
H	-7.86644400	-3.30156200	-2.99559900
H	-9.14007900	-2.84144100	-1.85235700
C	1.19991900	-6.07690600	2.93432900
O	0.13290000	-6.52253400	3.35193700
N	1.38887400	-4.73202600	2.71648100
C	0.22588400	-3.86523800	2.73964000
C	-0.54251800	-3.96097800	1.40876100
S	-2.32365500	-3.52651900	1.54755400
O	4.86165300	-3.42059900	-3.33112200
C	-1.51187500	-0.21982900	-0.60725000
C	-1.42184000	-1.06916400	0.44086700
C	-2.54624300	-1.89424500	0.90783700
O	-3.70314300	-1.45011200	0.94316200
C	-0.42627700	0.77643500	-0.87559000
C	-2.63776100	-0.21953100	-1.60021900
H	-3.16457700	0.74028800	-1.57572100
H	-2.22187900	-0.32400800	-2.60811200
H	-3.36399900	-1.01059000	-1.42928300
H	-0.86215900	1.73540900	-1.17717000
H	-0.52114800	-1.09509300	1.04469900
H	0.20605300	0.94073500	-0.00205100
H	-0.55731400	-5.00395100	1.08659500
H	-0.06299300	-3.36956900	0.62922900
H	-0.41876300	-4.18371600	3.56072200
H	0.54459000	-2.83628800	2.92728100
H	2.16989400	-4.43651800	2.13176700
C	2.38182900	-6.96675200	2.60349400
H	2.41416500	-7.13934900	1.52050600
H	3.33882000	-6.50856900	2.86759800
H	2.26074900	-7.92186000	3.11414000
H	-0.63237000	-4.79449900	-6.31137700
H	1.11014500	-4.94010900	-5.82124900
H	5.76074000	-6.95205200	-2.77129000
H	8.25286000	-1.63799700	-4.47356100
H	9.77230400	-2.38479800	-3.80091700
H	8.35562100	0.18536300	-4.11300800
H	8.64575600	1.78989000	-4.91033400
H	0.80321800	2.52461500	7.38238800
H	2.57472000	2.54131000	7.52344200
H	6.90003600	6.58221200	-0.26781200
H	5.08531300	6.70853700	-0.20128300
H	3.45801700	9.25527200	-1.86847000
H	4.19364000	7.67157100	-1.51623700
H	3.34710100	8.70571000	2.91044600
H	4.38915400	8.01303500	4.21982600
H	-2.04031200	8.33072100	3.59408000
H	-2.37530600	7.33614800	2.15204900
H	-5.05256300	5.80220500	-5.01782200
H	-6.46377700	5.04078600	-4.19938000
H	-8.49892500	0.78873500	-5.86126800
H	-7.90852600	2.25439000	-6.73569800
H	-11.23490400	0.74298200	-4.23234900
H	-9.49322100	1.21905400	-4.35992700
H	-9.06760900	-4.55009600	0.81503900
H	-8.97527800	-6.18193000	0.03790300
H	-4.47152100	-5.28303100	3.91698800
H	-6.25486800	-5.07631600	3.84291800

H	-3.38633300	-2.37589600	8.17403400
H	-1.62577200	-2.13514300	8.35386400
H	0.47055600	0.32501600	-6.55123100
H	0.70362000	2.01630700	-5.91047100
H	3.94142800	-3.45993400	-3.01789400
H	5.07254400	-2.46926300	-3.36433100
H	4.73467900	0.04706400	-4.23903500
O	4.04558300	0.68538000	-4.97245500
H	4.03075300	0.24031600	-5.83201800
H	2.98151000	0.48903000	-4.52564500
O	-3.17281500	2.71447200	3.57311000
H	-3.45582300	2.25789700	2.75485900
H	-3.87119000	2.51882600	4.21285200
O	-3.60329400	1.43066600	1.10355600
H	-3.72310300	0.46071500	1.05784000

E:P (Cys56$_B$-SH) (-13.0)

C	3.06955700	8.20000600	-2.05889800
C	2.22791900	7.50766400	-3.14230000
C	1.95971200	6.04650900	-2.86808800
C	0.91995500	5.65568800	-2.01621800
C	2.74779800	5.05157900	-3.46098300
C	0.66403900	4.30587500	-1.78066200
C	2.50114400	3.69970400	-3.22351300
C	1.45321800	3.32654200	-2.38324800
H	2.56270000	8.18118900	-1.09009100
H	1.27246200	8.03751500	-3.24374800
H	2.73686400	7.60117000	-4.10901300
H	0.31077900	6.41521400	-1.53394500
H	3.55711100	5.34373800	-4.12571100
H	-0.15364400	4.01933500	-1.12718100
H	3.10370400	2.93100100	-3.69533200
H	1.26731700	2.27679400	-2.20543300
C	5.83339800	6.23836600	-0.29668700
C	5.68921700	4.83570100	-0.88781000
C	4.52811000	4.06693400	-0.25651300
S	4.81227800	3.53570300	1.47920500
C	6.11530400	2.27506000	1.23517500
H	5.87002900	6.17230900	0.79627000
H	5.49081900	4.90397200	-1.96582900
H	6.62044100	4.26635500	-0.78478700
H	3.63847600	4.69919600	-0.20848900
H	4.27748700	3.18304200	-0.84235100
H	6.24642000	1.76714900	2.19315600
H	5.83939000	1.53313200	0.48160600
H	7.06512300	2.73352200	0.95055800
C	-2.13754700	7.45374500	2.79086300
C	-0.70140500	7.00177300	2.99122000
O	0.03627700	7.60490000	3.77932900
H	-2.79236600	6.82591200	3.40802000
N	-0.27836900	5.90063800	2.31730700
C	1.06424600	5.34378100	2.58908800
C	2.19023800	6.29808800	2.12701500
O	2.53839900	6.37164500	0.95050000
C	1.07077300	4.01990100	1.81421100
C	-0.40769100	3.61821800	1.75505200
C	-1.13906500	4.95527500	1.58520100
H	1.15897400	5.17965600	3.66967500
H	1.45621800	4.21262100	0.81064100
H	1.71542300	3.27644000	2.28672600
H	-0.61896600	2.93021200	0.93119700
H	-0.74286400	3.14985700	2.68474200
H	-1.21451100	5.24669400	0.52946300
H	-2.13970600	4.91574700	2.01906200
N	2.76732000	6.99913100	3.13833700
C	3.70630600	8.08197400	2.85735400
H	2.13276600	7.14997200	3.91592900
H	4.43294100	7.74115600	2.11955700
C	1.67610900	2.38024600	7.08936900
C	1.72329900	1.97908800	5.59816600
C	2.66113600	0.84144900	5.26794800
C	2.23860000	-0.48701700	5.42804100
C	3.95474700	1.07720200	4.78722300
C	3.08197200	-1.55206300	5.11284700
C	4.80497400	0.01317800	4.47919000
C	4.37180900	-1.30320700	4.63952100
H	1.65408900	1.49877800	7.73690300
H	0.70997500	1.69151000	5.29311000
H	1.98248400	2.85794100	4.99655100
H	1.23161800	-0.68479000	5.78901600
H	4.28910400	2.09980600	4.63280200
H	2.72703100	-2.57432100	5.20672200
H	5.80297000	0.21258400	4.10022900
H	5.02484000	-2.12838700	4.37792500
C	0.18226000	-5.14414800	-5.20790300
C	-0.07227900	-3.93182200	-4.33743700
O	-0.39741800	-2.84434200	-4.80053600
H	-0.00601500	-6.06615400	-4.64726200
N	0.02904200	-4.16936900	-2.98867400
C	0.15316200	-3.05473100	-2.08386600
C	1.61171600	-2.90527200	-1.66010400
O	2.46831500	-3.62659600	-2.17388400
H	0.56913800	-4.98350500	-2.71690600
H	-0.15457600	-2.15454500	-2.62071400
H	-0.50229800	-3.17089900	-1.21644900
N	1.89869400	-1.99232300	-0.71671800
C	3.28041400	-1.65414500	-0.40794600
C	4.00843900	-2.85428700	0.20913600
O	3.41246500	-3.76319100	0.81185800
C	3.35816100	-0.43318400	0.53562800
C	3.06447700	0.85294300	-0.23188700
C	2.47281800	-0.58477900	1.77572300
H	1.17089100	-1.36208300	-0.42254100
H	3.78946300	-1.39663500	-1.34194800
H	4.39787200	-0.39191200	0.87339100
H	3.03521200	1.70840700	0.44602500
H	3.83379400	1.02761600	-0.98387300
H	2.12000100	0.79464700	-0.76804800
H	2.69864700	0.20082300	2.49818900
H	1.40770300	-0.49475600	1.53077500
H	2.62598900	-1.54752700	2.26498300
N	5.33733300	-2.79524600	0.06103600
C	6.24271500	-3.76324000	0.67852800
C	5.88964800	-5.21057500	0.28028200
O	5.91572300	-6.12506000	1.10151200
C	6.29722800	-3.60597600	2.19609600
H	5.74051500	-2.02290400	-0.48887400
H	7.21957400	-3.55483900	0.23052800
H	6.97775700	-4.34414400	2.62313900

H	6.64031400	-2.59928300	2.45279500
H	5.30587600	-3.77192200	2.62309800
N	5.63387000	-5.37574500	-1.04147000
C	5.23501300	-6.65587100	-1.59348500
C	3.78478800	-6.61164700	-2.09175400
O	2.85196000	-6.33159500	-1.05631100
H	5.61071100	-4.57283000	-1.67168100
H	5.32892900	-7.38857900	-0.78876600
H	3.70244000	-5.87498800	-2.90323100
H	3.51278400	-7.59323500	-2.49769100
H	3.09074800	-5.46975700	-0.67568400
C	0.10973100	0.79921300	-5.88565900
C	0.15762700	0.31555600	-4.44216100
S	1.85345700	0.07926300	-3.75831800
H	-0.93526800	0.99580300	-6.16153600
H	-0.29726800	1.06199700	-3.78435500
H	-0.38694900	-0.62552000	-4.34342400
H	0.15966300	0.25176300	-1.71130300
C	8.85362500	-2.57320900	-4.12690000
C	8.08680300	-3.42500000	-3.11456100
O	8.00920500	-2.80434700	-1.83407900
H	9.02134400	-3.13385400	-5.05466700
H	7.09546500	-3.68323100	-3.49671500
H	8.62483000	-4.36593800	-2.94773600
H	7.46576100	-1.99472000	-1.88213800
C	7.90762700	1.12352800	-4.42741400
C	7.32354400	1.87025300	-3.21863900
C	5.92720900	1.35992400	-2.81567200
C	5.91920300	-0.13342300	-2.48592600
O	6.33770600	-0.48620700	-1.34108300
O	5.52747000	-0.92792600	-3.39025900
H	7.09862300	0.86320900	-5.12109000
H	7.99564600	1.78213800	-2.35715600
H	7.23746600	2.94096100	-3.44084700
H	5.59237100	1.91872600	-1.93882000
H	5.22052100	1.53756000	-3.63222500
C	-5.47902800	4.79482100	-4.47320100
C	-4.93151400	5.31191100	-3.12731400
C	-3.43082900	4.98352000	-2.94939100
C	-5.77093700	4.80547600	-1.94781400
C	-3.10483700	3.50992800	-2.66652200
H	-5.09210700	3.79063700	-4.68470600
H	-5.01355100	6.40778900	-3.13744400
H	-2.88872200	5.30378200	-3.84818800
H	-3.03365300	5.59048100	-2.12502900
H	-5.35563000	5.13642500	-0.98939500
H	-6.79948100	5.17553100	-2.01328700
H	-5.82169800	3.71408400	-1.92211200
H	-2.02016800	3.36114600	-2.66525800
H	-3.49156500	3.19093800	-1.69224700
H	-3.52409400	2.84153700	-3.42642400
C	-7.71809900	0.72147600	-6.51609000
C	-6.39060700	0.69702000	-5.74331900
C	-6.13962000	-0.59706700	-4.95059500
C	-6.18828800	-1.84942300	-5.83329400
C	-4.80506200	-0.51931700	-4.20404800
H	-7.67141300	0.09946200	-7.41547600
H	-5.55283300	0.86867000	-6.43387700
H	-6.37351200	1.53538200	-5.03407100
H	-6.93861000	-0.67962400	-4.19966000
H	-6.00325800	-2.74914900	-5.23749300
H	-7.16005600	-1.96905700	-6.32176500
H	-5.42046800	-1.80401300	-6.61551500
H	-4.66042500	-1.39483300	-3.56454900
H	-3.96332400	-0.47467900	-4.90604700
H	-4.76046400	0.37026700	-3.56854500
C	-10.29291500	0.43614700	-3.72453100
C	-10.32974500	1.27784000	-2.43768300
C	-9.08371200	1.00578000	-1.61442100
C	-9.14413800	0.20855800	-0.46464900
C	-7.82607700	1.45310100	-2.04506900
C	-7.98760000	-0.15211100	0.23099200
C	-6.66706100	1.09573600	-1.35546700
C	-6.74297200	0.28497600	-0.22193500
H	-9.97539300	-0.57902400	-3.45173500
H	-10.40668200	2.34444800	-2.68234200
H	-11.21578800	1.02368300	-1.84593500
H	-10.11022300	-0.15289000	-0.12125600
H	-7.75305500	2.07798000	-2.93182600
H	-8.04585600	-0.79227900	1.10662600
H	-5.69978700	1.44444000	-1.70241200
H	-5.83734100	-0.01523600	0.29171700
C	-2.34410900	-2.21655800	7.87319900
C	-2.34209600	-1.74043900	6.42836300
O	-2.62728700	-2.48574200	5.49122500
H	-2.18091700	-3.29345700	7.89585600
N	-2.03332500	-0.42055600	6.25946300
C	-2.29337200	0.31939500	5.01336800
C	-3.81382500	0.28646100	4.73838100
O	-4.57423200	1.00321200	5.39301500
C	-1.39301600	-0.12329600	3.86343000
H	-1.98248400	0.14044800	7.09685900
H	-2.08670500	1.36792700	5.22768600
H	-1.65459500	0.44781500	2.97039100
H	-0.34574000	0.07180700	4.10458800
H	-1.49232800	-1.19172800	3.66796100
N	-4.22834400	-0.57988700	3.79345000
C	-5.61589600	-0.85829100	3.48534200
C	-5.89772900	-2.34932400	3.26563000
O	-6.88633200	-2.69809500	2.61986200
H	-3.54074500	-1.12077200	3.29641200
H	-6.22201800	-0.48485700	4.31547300
H	-5.94007200	-0.33929900	2.57969800
N	-5.03054500	-3.21551100	3.83753000
C	-5.18536100	-4.65574900	3.68702500
H	-4.25252900	-2.87444600	4.39294700
H	-5.17311400	-4.93688300	2.63000500
C	-8.75252900	-5.27252800	0.17478200
C	-7.35389300	-4.93884600	-0.36083000
C	-7.14347500	-3.46243000	-0.73776500
C	-5.66761400	-3.18835600	-1.04493700
C	-8.01851000	-3.02053600	-1.91756800
H	-9.52601100	-5.03421000	-0.56430400
H	-6.61459700	-5.20379700	0.40582000
H	-7.13095300	-5.56856200	-1.23511300
H	-7.41016700	-2.86394200	0.13994100
H	-5.52174400	-2.13495400	-1.30332100
H	-5.03550000	-3.40877000	-0.17990800
H	-5.32013000	-3.79554500	-1.89125400
H	-7.85023000	-1.96373900	-2.14553600

H	-7.78240000	-3.60391100	-2.81735100
H	-9.08611100	-3.14299800	-1.70934700
C	1.35764700	-5.83850200	3.24819800
O	0.30976600	-6.24896200	3.74385500
N	1.51936100	-4.51824300	2.89411000
C	0.34021600	-3.67285600	2.89145200
C	-0.49848000	-3.93040600	1.62717200
S	-2.28088300	-3.51600300	1.80076600
O	4.92574900	-3.53015300	-3.22407300
C	-1.55372200	-0.34039000	-0.54666100
C	-1.43850700	-1.10505800	0.56014500
C	-2.54600600	-1.92179000	1.08536700
O	-3.70631200	-1.49400700	1.10197400
C	-0.47663100	0.63818800	-0.90577000
C	-2.69867100	-0.40746400	-1.51446500
H	-3.25989200	0.53274600	-1.49091100
H	-2.30521300	-0.51517200	-2.53087500
H	-3.39232800	-1.21896500	-1.30846800
H	-0.92066000	1.56446300	-1.28553200
H	-0.53585300	-1.06721700	1.16184700
H	0.15912300	0.88307200	-0.05421200
H	-0.51263200	-5.00443700	1.43303500
H	-0.07175900	-3.43441700	0.75603300
H	-0.25175400	-3.91396400	3.77609600
H	0.64384100	-2.62457800	2.95125300
H	2.27208600	-4.27017500	2.25122700
C	2.53766400	-6.74570000	2.96914400
H	2.57424200	-6.97806500	1.89748100
H	3.49388500	-6.27162700	3.20761700
H	2.41616200	-7.66903000	3.53467800
H	-0.52348800	-5.11241600	-6.04449700
H	1.22163400	-5.19412300	-5.54913000
H	5.91531900	-6.94061400	-2.40300500
H	8.28992900	-1.67048400	-4.38479800
H	9.82617900	-2.34763100	-3.67702800
H	8.35429600	0.17111500	-4.12311600
H	8.60761600	1.73763200	-5.00370100
H	0.75225000	2.94782300	7.24289600
H	2.52291000	3.01047700	7.38084200
H	6.75853300	6.72816600	-0.61802200
H	4.94216600	6.81919600	-0.55604500
H	3.25903300	9.23764500	-2.35284600
H	4.02883900	7.69083700	-1.91878800
H	3.16025600	8.93852200	2.44846800
H	4.21735700	8.33856300	3.79124600
H	-2.21767700	8.48250500	3.15726900
H	-2.53085500	7.40623700	1.77003600
H	-5.17351300	5.43699200	-5.30589700
H	-6.56856900	4.68860700	-4.44558700
H	-8.50997000	0.31290200	-5.87979600
H	-7.95194900	1.74349800	-6.83098000
H	-11.24461500	0.29363800	-4.24748100
H	-9.51424400	0.80062000	-4.40229500
H	-8.96195600	-4.67688900	1.06935100
H	-8.83414800	-6.34512700	0.37917000
H	-4.35076800	-5.14497100	4.20017100
H	-6.13819900	-4.98131000	4.11751400
H	-3.32929000	-1.99441600	8.29674100
H	-1.57445700	-1.70615700	8.46162100
H	0.46922600	0.00956100	-6.55297300
H	0.66356400	1.73688700	-6.00311900
H	4.05201800	-3.50145900	-2.78990700
H	5.25932600	-2.59881400	-3.16379400
H	2.31351500	-0.94442500	-4.54010800
O	3.78116200	-1.93564200	-5.34674200
H	4.41550600	-1.30064100	-4.96653800
H	4.01898500	-2.73662200	-4.84445200
O	-3.15904900	2.94034200	3.47541700
H	-3.45055500	2.43605700	2.69050800
H	-3.82358300	2.73425000	4.14897800
O	-3.57598900	1.42963800	1.11537300
H	-3.73572600	0.46721400	1.16710500

Direct decarboxylation mechanism:
Deprotonated carboxylate group of MG-CoA
Deprotonated Glu72$_B$

E:S$_{dpGlu72}$ (0.0)

C	3.28310100	8.22425000	-1.44264500
C	2.44622200	7.60704400	-2.57794400
C	2.23852000	6.11092500	-2.46836700
C	1.24227900	5.58389400	-1.63647600
C	3.01770400	5.22065900	-3.21951700
C	1.01808100	4.20885100	-1.57154300
C	2.80634300	3.84115800	-3.15734600
C	1.80081200	3.33734000	-2.33082500
H	2.77699900	8.13609300	-0.47741900
H	1.46785600	8.10315600	-2.61106900
H	2.93339700	7.82366000	-3.53652700
H	0.63149100	6.25823800	-1.04295400
H	3.78900100	5.61552300	-3.87677100
H	0.21385000	3.82035200	-0.95517100
H	3.40263100	3.16658700	-3.76616300
H	1.63324000	2.26933700	-2.27344700
C	6.02667100	6.09661600	0.15101200
C	5.86509400	4.74091100	-0.54573200
C	4.68330900	3.93523700	0.00100300
S	4.93071900	3.24410500	1.68649200
C	6.14375200	1.92186600	1.33330200
H	6.06525900	5.94773800	1.23585600
H	5.68527600	4.89457800	-1.61800800
H	6.78833500	4.15321700	-0.47330300
H	3.80159400	4.57284700	0.09132000
H	4.42994200	3.10763300	-0.66247300
H	6.28784900	1.37016500	2.26504600
H	5.79148800	1.22643200	0.56804400
H	7.10595700	2.33305800	1.01825900
C	-1.90637800	7.19951800	3.37516200
C	-0.47582300	6.70821500	3.53880800
O	0.24198200	7.18455700	4.43404700
H	-2.57083200	6.53162800	3.93524800
N	-0.03162900	5.71702900	2.72915600
C	1.27511500	5.08314700	3.00339400
C	2.43231700	6.01487700	2.60953800
O	2.81924600	6.14079100	1.44895000
C	1.25592500	3.79477600	2.17057600

C	-0.23036700	3.52551300	1.92564800
C	-0.81767400	4.92868200	1.76358900
H	1.33490000	4.87697900	4.07800800
H	1.75964100	3.98900500	1.22111700
H	1.78479400	2.98077300	2.67027200
H	-0.39891800	2.92211300	1.03830000
H	-0.70404900	3.02371400	2.77613700
H	-0.64448900	5.30427200	0.74763800
H	-1.88836600	4.97369700	1.96430500
N	2.98727800	6.66203800	3.66828800
C	3.94673300	7.73386400	3.44703700
H	2.32122500	6.79730800	4.42510700
H	4.65613200	7.42660600	2.67887000
C	1.85385100	1.76606000	7.26087900
C	1.88402600	1.47874600	5.74256800
C	2.76457000	0.32436000	5.32527300
C	2.29087500	-0.99220700	5.43425400
C	4.04845500	0.53268400	4.80790200
C	3.07645300	-2.07242100	5.03460000
C	4.84267000	-0.54776500	4.41792700
C	4.35985700	-1.85142900	4.53062700
H	1.81942400	0.83832300	7.83955500
H	0.85916000	1.26340200	5.41937400
H	2.18614000	2.38686600	5.20882000
H	1.28632100	-1.16566000	5.81310600
H	4.41746100	1.54818600	4.68894200
H	2.68049200	-3.08254700	5.08251600
H	5.83354900	-0.37024000	4.00976500
H	4.96803700	-2.68805200	4.20632400
C	0.17205600	-4.80614300	-5.54728900
C	-0.03522400	-3.66175400	-4.58203700
O	-0.10049500	-2.49453500	-4.95758100
H	-0.03212000	-5.76873800	-5.06656500
N	-0.20583600	-4.04152000	-3.27727200
C	-0.05504800	-3.05127800	-2.24027700
C	1.43102500	-2.86232100	-1.92152000
O	2.28095800	-3.43810200	-2.60476100
H	0.17043500	-4.95081500	-3.03811500
H	-0.46491000	-2.10698600	-2.60213600
H	-0.61972600	-3.34240900	-1.35317200
N	1.72957300	-2.05437300	-0.89347900
C	3.10681100	-1.72500000	-0.56805400
C	3.85469200	-2.97051200	-0.05575100
O	3.26180800	-3.94137000	0.44989800
C	3.16550100	-0.57322000	0.46302500
C	2.73510300	0.75158000	-0.16937600
C	2.36625900	-0.87451100	1.73481900
H	0.97991600	-1.56294300	-0.43018600
H	3.60890900	-1.39393000	-1.48322800
H	4.22085700	-0.48992100	0.74089300
H	2.82820200	1.56730000	0.55200000
H	3.35012900	0.98911800	-1.04054700
H	1.69556100	0.71273400	-0.51009900
H	2.56497400	-0.11439800	2.49266000
H	1.28449200	-0.86517700	1.55023000
H	2.62815300	-1.84525900	2.15753900
N	5.18326400	-2.88290800	-0.18104500
C	6.10861400	-3.87487300	0.36512300
C	5.81517300	-5.30666700	-0.13131000
O	5.85785100	-6.26899900	0.63628800

C	6.15241700	-3.82218100	1.88996300
H	5.58840400	-2.08278200	-0.69174000
H	7.08290500	-3.60863900	-0.05925900
H	6.85952900	-4.56050900	2.27160000
H	6.45096400	-2.82175100	2.21641800
H	5.16615000	-4.05486700	2.29675000
N	5.60863000	-5.40118900	-1.46716700
C	5.22219000	-6.65600500	-2.08972200
C	3.76616300	-6.57112600	-2.57438000
O	2.83861800	-6.36415200	-1.51971500
H	5.52506600	-4.54890700	-2.03857400
H	5.32072000	-7.43399800	-1.32936400
H	3.68812400	-5.77481800	-3.32927500
H	3.48523000	-7.51809700	-3.05259800
H	3.07929000	-5.54002800	-1.06174200
C	0.18654700	1.17199900	-5.78452000
C	0.18105400	0.60693100	-4.36854600
S	1.83240800	0.03749100	-3.78664300
H	-0.84710600	1.42464600	-6.06114400
H	-0.13513200	1.37101300	-3.65314900
H	-0.49224300	-0.24775100	-4.29357300
H	1.77700400	-1.17392200	-4.37289000
C	8.88823600	-2.45178700	-4.34023200
C	8.11002100	-3.34381400	-3.36541400
O	8.09785200	-2.80461500	-2.04784100
H	9.03447300	-2.95566700	-5.30400000
H	7.09749800	-3.53375700	-3.73303400
H	8.61294300	-4.31596100	-3.28158500
H	7.49005000	-2.03943900	-2.00275700
C	7.99777400	1.27074300	-4.35983500
C	7.51709300	1.92633000	-3.05047300
C	6.12300600	1.45440200	-2.58717400
C	5.99455400	-0.06535000	-2.47785400
O	6.36504300	-0.61500400	-1.39458800
O	5.54668200	-0.68800200	-3.48892000
H	7.15122600	1.11179700	-5.03402700
H	8.24595300	1.73532800	-2.25293700
H	7.46610100	3.01566800	-3.17042900
H	5.91468700	1.89223000	-1.60970300
H	5.38224900	1.80912200	-3.30564200
C	-5.33110900	5.13490400	-4.04164900
C	-4.79829800	5.63021900	-2.68435900
C	-3.27364400	5.87505300	-2.71706400
C	-5.20214300	4.70281600	-1.53514200
C	-2.40349700	4.63886300	-2.97626200
H	-4.92752200	4.14709700	-4.29005000
H	-5.26669900	6.60975800	-2.50097100
H	-3.06004100	6.63497400	-3.48292600
H	-2.97724400	6.31576200	-1.75629000
H	-4.95213300	5.12632200	-0.55848800
H	-6.27851100	4.49895300	-1.54726700
H	-4.67965800	3.74534900	-1.59833600
H	-1.34868100	4.92369400	-3.03402300
H	-2.49510600	3.90188300	-2.17368300
H	-2.66086800	4.15288100	-3.92438400
C	-7.64441500	1.25700100	-6.36415100
C	-6.30034600	1.14951100	-5.63715300
C	-5.99888700	-0.23747000	-5.04773500
C	-6.08070400	-1.35852700	-6.09003100
C	-4.62539000	-0.23183100	-4.37481600

H	-7.62825900	0.70828100	-7.31233600
H	-5.48876800	1.43274600	-6.32266300
H	-6.27262400	1.87890400	-4.81785700
H	-6.75366900	-0.43806800	-4.27330300
H	-5.83506100	-2.32389900	-5.63555700
H	-7.08014500	-1.44323300	-6.52788500
H	-5.36844300	-1.18334600	-6.90649400
H	-4.41894800	-1.19031800	-3.89281800
H	-3.83024800	-0.04705500	-5.10800400
H	-4.56532100	0.54890500	-3.61179100
C	-10.20721400	0.80501000	-3.58301200
C	-10.20901300	1.55598400	-2.24199800
C	-8.92058200	1.24821200	-1.50214100
C	-8.90105900	0.32575400	-0.44914600
C	-7.70103600	1.78676700	-1.93549300
C	-7.70238400	-0.06564000	0.15146900
C	-6.49975900	1.39946300	-1.34167600
C	-6.49597000	0.46878500	-0.30227900
H	-9.90218100	-0.23098000	-3.38540300
H	-10.31256500	2.63541800	-2.40899800
H	-11.06459900	1.24339000	-1.63285700
H	-9.83721400	-0.10971400	-0.10798200
H	-7.68921300	2.51246500	-2.74535700
H	-7.70766300	-0.79863100	0.95356800
H	-5.56503800	1.83167200	-1.67843000
H	-5.55385700	0.17607100	0.14893300
C	-2.23209700	-2.81559300	7.73157200
C	-2.21574100	-2.27483000	6.31147400
O	-2.45445400	-2.98904300	5.34120300
H	-2.08591700	-3.89529700	7.69400100
N	-1.92674600	-0.94106600	6.19703300
C	-2.26772700	-0.22858100	4.95768500
C	-3.74209500	-0.55829400	4.62638900
O	-4.56936700	-0.62245300	5.53578100
C	-1.25012300	-0.50217200	3.85346100
H	-2.01880000	-0.40447400	7.04713600
H	-2.25990800	0.84141000	5.18638200
H	-1.46249300	0.12432200	2.98491100
H	-0.24368000	-0.27036700	4.20833400
H	-1.28022600	-1.55478100	3.56903400
N	-4.05178100	-0.79148000	3.32843500
C	-5.41051000	-1.11646600	2.94593900
C	-5.77727600	-2.60449400	2.91418800
O	-6.82534000	-2.94865900	2.35913700
H	-3.42752800	-0.47483900	2.59044100
H	-6.10974500	-0.62740700	3.63093000
H	-5.58696400	-0.74467800	1.94071000
N	-4.94421900	-3.46517900	3.53569900
C	-5.13507100	-4.89621400	3.39694600
H	-4.07189900	-3.13076100	3.92994900
H	-5.14138600	-5.16620700	2.33396400
C	-8.73199500	-5.19844600	-0.12654000
C	-7.38727200	-4.83245600	-0.75820400
C	-7.22745600	-3.35443100	-1.15851800
C	-5.90914200	-3.15484600	-1.91203100
C	-8.38914700	-2.82495000	-2.00914900
H	-9.54853900	-4.92128900	-0.80605000
H	-6.57416100	-5.08249600	-0.06688800
H	-7.24097800	-5.45627900	-1.65378500
H	-7.17821200	-2.77401300	-0.23117100

H	-5.73087600	-2.09152000	-2.09726400
H	-5.06606800	-3.53916800	-1.33465800
H	-5.93504800	-3.66812200	-2.88345400
H	-8.18613800	-1.79826200	-2.32883600
H	-8.52658200	-3.43969300	-2.90938500
H	-9.33656700	-2.81134500	-1.46180600
C	1.45033000	-6.15583000	3.00166700
O	0.45169300	-6.60993100	3.55508900
N	1.61490100	-4.80825400	2.77000000
C	0.39977800	-4.00657700	2.76411400
C	-0.35929000	-4.24849000	1.44545600
S	-2.18416700	-4.23515000	1.57875000
O	4.94087100	-3.35499500	-3.34060700
C	-1.99733200	-0.67405500	-0.38498400
C	-1.80014100	-1.62364900	0.55557100
C	-2.74190200	-2.73520700	0.77759200
O	-3.93614100	-2.66215800	0.52214300
C	-1.36388600	0.67208500	-0.25361600
C	-2.41191900	1.61807000	0.43671900
O	-2.62769100	2.72526300	-0.09486400
O	-2.91970000	1.15844100	1.50436100
C	-2.95497900	-0.80520200	-1.52891200
H	-3.81542200	-0.14548800	-1.37808600
H	-2.46492700	-0.47315600	-2.45052900
H	-3.33469300	-1.81507000	-1.65653700
H	-0.48801700	0.64803400	0.40292100
H	-1.03534200	-1.47678200	1.30945400
H	-1.08056500	1.10390200	-1.21601000
H	-0.12860400	-5.25553200	1.09349800
H	-0.03381700	-3.55702700	0.66835500
H	-0.21357400	-4.31078200	3.61261300
H	0.65604700	-2.95230400	2.88479600
H	2.31393200	-4.54417800	2.07523300
C	2.55054500	-7.04251500	2.47261200
H	2.38483800	-7.21534400	1.40174100
H	3.54335100	-6.58920600	2.54946400
H	2.52719900	-7.99998700	2.99382800
H	-0.53798400	-4.70224500	-6.37431000
H	1.20854000	-4.84637100	-5.89858000
H	5.89307200	-6.89045400	-2.92277500
H	8.33683100	-1.52428100	-4.52659400
H	9.86677400	-2.27475900	-3.88177300
H	8.43147200	0.29170800	-4.13110700
H	8.70327600	1.91511200	-4.89505100
H	0.93973400	2.33433600	7.46229100
H	2.71194500	2.36016800	7.59210400
H	6.95719100	6.59510100	-0.14022300
H	5.14298100	6.70843300	-0.05899300
H	3.48669700	9.27785700	-1.66045400
H	4.23508900	7.69178300	-1.34715700
H	3.41154800	8.62629000	3.10619900
H	4.46716200	7.91302200	4.39361600
H	-1.96851300	8.19948600	3.81705600
H	-2.30637500	7.23346000	2.35640800
H	-5.02050400	5.83194600	-4.82695700
H	-6.42187600	5.04293500	-4.01438700
H	-8.43888500	0.81500100	-5.75382800
H	-7.86452700	2.30324000	-6.60069200
H	-11.16376900	0.71572200	-4.10844500
H	-9.42669700	1.20680900	-4.23742400

H	-8.92679900	-4.66758300	0.81115200
H	-8.82873100	-6.28193400	-0.00109100
H	-4.30507300	-5.43443900	3.86643200
H	-6.09028600	-5.23845000	3.80858200
H	-3.21116900	-2.61078100	8.17712200
H	-1.45114000	-2.36171000	8.35054600
H	0.53026000	0.42778500	-6.51039700
H	0.75522900	2.10635100	-5.83589900
H	3.99416200	-3.32460500	-3.12012300
H	5.21711200	-2.40798700	-3.38573100
O	-3.97188800	4.56735100	1.66115300
H	-3.96290800	4.01788700	2.46658900
H	-3.60964000	3.95335300	0.98881100
O	4.56341900	1.10947300	-5.41838600
H	4.95939200	0.41999000	-4.84135600
H	3.61941300	0.99779000	-5.22963500
O	-3.52090900	2.54964100	3.69033500
H	-4.36541200	2.17205800	3.96854300

TS1$_{dpGlu72}$ (+20.3)

C	3.16479900	8.17609600	-1.72011300
C	2.34594100	7.51216500	-2.84298800
C	2.19712500	6.01135400	-2.70794500
C	1.26069000	5.45891700	-1.82474300
C	2.98304800	5.14053500	-3.47479700
C	1.10434900	4.07662100	-1.72803900
C	2.83715600	3.75390800	-3.38124600
C	1.88956800	3.22296800	-2.50500000
H	2.65447300	8.11248900	-0.75556800
H	1.35004800	7.97222700	-2.88191600
H	2.82265300	7.73025500	-3.80634000
H	0.65895500	6.11748000	-1.20483500
H	3.71204300	5.55679600	-4.16630400
H	0.36542100	3.66131100	-1.05114300
H	3.44351100	3.09154600	-3.99439900
H	1.77397500	2.15045600	-2.41685900
C	5.92361200	6.13037900	-0.04778800
C	5.77902000	4.75012600	-0.69335200
C	4.60266900	3.95925000	-0.11907700
S	4.83175700	3.38217300	1.61083500
C	6.09083000	2.07975100	1.36621600
H	5.96113800	6.02176300	1.04185500
H	5.60226900	4.85799300	-1.77160300
H	6.70643800	4.17375700	-0.59276300
H	3.70944400	4.58628400	-0.08340000
H	4.37643700	3.08912100	-0.73486700
H	6.23500700	1.59458100	2.33369800
H	5.76953400	1.32670200	0.64348300
H	7.04493500	2.50018300	1.03927100
C	-2.02914400	7.26638900	3.11587500
C	-0.59633200	6.79650700	3.28824200
O	0.12353000	7.30122300	4.16310200
H	-2.68787900	6.61007900	3.69560800
N	-0.14884200	5.78902100	2.49780000
C	1.16721300	5.17604400	2.78033700
C	2.31436100	6.11727700	2.37483800
O	2.68871700	6.23703400	1.20991700
C	1.15823400	3.87608000	1.96670600
C	-0.33009300	3.56603600	1.79013000
C	-0.95185200	4.95007900	1.59078000
H	1.22836700	4.98175200	3.85727800
H	1.62297700	4.06835800	0.99752700
H	1.72356800	3.08308200	2.45868000
H	-0.52030900	2.89487600	0.95286700
H	-0.74490300	3.10868100	2.69443500
H	-0.82325000	5.28747200	0.55370700
H	-2.01419000	4.98354100	1.83897500
N	2.87276400	6.77739900	3.42440400
C	3.81841200	7.85878000	3.18515900
H	2.21489600	6.91058800	4.18768700
H	4.53838700	7.54140600	2.43067800
C	1.77064500	2.00450500	7.19445100
C	1.81234200	1.65202600	5.69149700
C	2.76640000	0.54052100	5.32174600
C	2.36929600	-0.79768900	5.46092300
C	4.04513300	0.80952500	4.82024800
C	3.21896900	-1.84103900	5.09665700
C	4.90539300	-0.23335700	4.47098200
C	4.49475000	-1.55957700	4.60502100
H	1.74597200	1.10039200	7.81063500
H	0.80259900	1.35509200	5.38309000
H	2.05295900	2.55320700	5.11587900
H	1.37310800	-1.02034200	5.83585000
H	4.35795100	1.84106600	4.67955300
H	2.87665700	-2.86952900	5.16292800
H	5.89202500	-0.01030300	4.07568400
H	5.15253200	-2.36755800	4.30586700
C	0.18970800	-5.01557700	-5.38692100
C	0.00081300	-3.80025500	-4.50794600
O	-0.01863800	-2.66545400	-4.98124500
H	-0.01244700	-5.94632000	-4.84771600
N	-0.19011900	-4.06682200	-3.18233500
C	0.00907800	-3.00927600	-2.22084700
C	1.49504700	-2.90002900	-1.88216200
O	2.32283900	-3.54682000	-2.53199800
H	0.12847200	-4.97632900	-2.87252600
H	-0.30951000	-2.06893600	-2.67206100
H	-0.60369500	-3.15679700	-1.32918000
N	1.82008300	-2.07386700	-0.87645700
C	3.20729900	-1.76738100	-0.57515900
C	3.93501000	-2.99746700	-0.00847400
O	3.33564200	-3.96886700	0.48636900
C	3.31150300	-0.57513500	0.40366400
C	2.91757500	0.72848700	-0.29053500
C	2.51833400	-0.79875600	1.69613300
H	1.08949900	-1.49187300	-0.49207500
H	3.71545400	-1.49175100	-1.50563700
H	4.37159700	-0.51163000	0.66630700
H	2.99269200	1.56828800	0.40500300
H	3.56761800	0.92334100	-1.14680400
H	1.89348400	0.68538400	-0.66695700
H	2.74696600	-0.01202300	2.41816300
H	1.43672000	-0.77220100	1.52296600
H	2.76023800	-1.75767000	2.15862500
N	5.26811200	-2.89655700	-0.07068900
C	6.17143100	-3.87545900	0.52785300
C	5.87382500	-5.31576700	0.06553900
O	5.93073400	-6.26256500	0.85195100
C	6.17704700	-3.77638800	2.05150700

H	5.68546000	-2.10773500	-0.58676000
H	7.15831300	-3.63096100	0.12002800
H	6.84446700	-4.52938900	2.47382200
H	6.50608400	-2.77815300	2.35491800
H	5.17158500	-3.95693300	2.43876700
N	5.64452000	-5.43462100	-1.26312800
C	5.24657700	-6.69839800	-1.85481000
C	3.79288400	-6.62383500	-2.34518100
O	2.86443400	-6.40843000	-1.29612700
H	5.55789200	-4.59492600	-1.85080000
H	5.33984400	-7.45918700	-1.07645700
H	3.71256500	-5.84056000	-3.11310900
H	3.52047500	-7.57970500	-2.81145100
H	3.08349000	-5.56229000	-0.86685900
C	0.14820200	0.95093000	-5.82757200
C	0.17021100	0.42886300	-4.39519800
S	1.83973700	-0.11261600	-3.84314000
H	-0.89047300	1.18086600	-6.10524700
H	-0.13737100	1.20499800	-3.68998500
H	-0.49561700	-0.42703300	-4.28257700
H	1.76013700	-1.34759500	-4.37619800
C	8.87944400	-2.53825300	-4.23728600
C	8.12215200	-3.41603800	-3.23391900
O	8.12865600	-2.85269100	-1.92699100
H	9.03452500	-3.06951800	-5.18506300
H	7.10601200	-3.62236400	-3.58099900
H	8.63597700	-4.38175800	-3.14060600
H	7.53220300	-2.07725700	-1.89244400
C	7.95392500	1.17279600	-4.38631100
C	7.50919500	1.88213900	-3.09177900
C	6.14854200	1.41368000	-2.53604700
C	6.03322600	-0.10533700	-2.40076700
O	6.45044700	-0.63678700	-1.32598500
O	5.54788800	-0.74061400	-3.38580100
H	7.09824700	0.99242700	-5.04192200
H	8.27794400	1.74287200	-2.32113300
H	7.43401300	2.96347000	-3.26243400
H	6.00185400	1.86384300	-1.55296200
H	5.36088700	1.75849000	-3.20803000
C	-5.41191500	4.91783500	-4.23543700
C	-4.88265900	5.44118400	-2.88875600
C	-3.34624700	5.60172400	-2.88806400
C	-5.37328300	4.58221300	-1.71796900
C	-2.53334200	4.31081300	-3.04747800
H	-5.00592400	3.92462300	-4.45758000
H	-5.29906200	6.45069100	-2.75124800
H	-3.06876100	6.30124300	-3.68889400
H	-3.05378800	6.08635500	-1.94695100
H	-5.03846900	4.99230800	-0.75868200
H	-6.46693100	4.52689200	-1.69626500
H	-4.99711800	3.55958200	-1.78570300
H	-1.46153400	4.53088000	-3.03924100
H	-2.72812300	3.60757900	-2.23417200
H	-2.75460200	3.80185400	-3.99173200
C	-7.68143600	0.94127400	-6.43067000
C	-6.33667100	0.85745100	-5.69851400
C	-6.06168800	-0.49476900	-5.01904600
C	-6.18229600	-1.68014900	-5.98399300
C	-4.68217800	-0.48396400	-4.35741300
H	-7.66208600	0.36072200	-7.35968200

H	-5.52069200	1.08127000	-6.40055200
H	-6.29387200	1.63768100	-4.92761900
H	-6.81451400	-0.62300500	-4.22799100
H	-5.95815300	-2.61863800	-5.46687200
H	-7.18733800	-1.76703600	-6.40877100
H	-5.47206500	-1.57852300	-6.81449100
H	-4.51072300	-1.39727300	-3.78245700
H	-3.88654200	-0.40520800	-5.10864600
H	-4.57846400	0.36128300	-3.67254400
C	-10.24808600	0.55962200	-3.64263100
C	-10.23996700	1.34197500	-2.31700800
C	-8.95984300	1.02364900	-1.56158300
C	-8.97435300	0.16824300	-0.45261700
C	-7.71736200	1.47193300	-2.03364400
C	-7.78924200	-0.26151200	0.14997700
C	-6.52953800	1.04141200	-1.44028700
C	-6.55924700	0.15894800	-0.35879900
H	-9.93451100	-0.46883700	-3.41950700
H	-10.32262500	2.41925600	-2.50945800
H	-11.10307400	1.06090200	-1.70326500
H	-9.92822600	-0.19135100	-0.07357600
H	-7.67712900	2.14811300	-2.88453800
H	-7.81399400	-0.94854500	0.99161300
H	-5.57518600	1.38247800	-1.82575900
H	-5.63386200	-0.22323200	0.06203200
C	-2.27310400	-2.59735900	7.80997700
C	-2.27136600	-2.13972400	6.36190500
O	-2.64736600	-2.88483200	5.46215900
H	-2.12111900	-3.67716900	7.82306900
N	-1.84661000	-0.86042400	6.14175100
C	-2.02910000	-0.19629800	4.83910600
C	-3.54215600	-0.18580200	4.52507800
O	-4.29879400	0.44607800	5.28108400
C	-1.10125700	-0.77112400	3.77634300
H	-1.78294800	-0.25666800	6.94737100
H	-1.78504600	0.85658300	5.00858900
H	-1.21799000	-0.23449600	2.83528200
H	-0.06321400	-0.67592100	4.10134500
H	-1.30930000	-1.82696900	3.60579700
N	-3.96361700	-0.87702300	3.45769500
C	-5.36033200	-1.06958300	3.12750800
C	-5.77881800	-2.54342800	3.05075600
O	-6.81895000	-2.85627600	2.46620200
H	-3.29159900	-1.29103400	2.82621000
H	-5.95265900	-0.55923400	3.89030800
H	-5.58451400	-0.63339800	2.15506700
N	-4.97665800	-3.41887400	3.69161600
C	-5.14324200	-4.85190300	3.54095200
H	-4.11231800	-3.09279400	4.10976100
H	-5.13364100	-5.12444700	2.48047200
C	-8.72652600	-5.30828300	0.02025100
C	-7.35623300	-4.96748100	-0.57726200
C	-7.16708900	-3.50354300	-1.01049200
C	-5.76088100	-3.29501900	-1.58167600
C	-8.22034900	-3.03691300	-2.02400600
H	-9.52562500	-5.05058600	-0.68619900
H	-6.57431400	-5.20667500	0.15330400
H	-7.17770600	-5.61769900	-1.44774000
H	-7.25221400	-2.88846400	-0.10900800
H	-5.62081600	-2.25200900	-1.88157800

H	-4.99129700	-3.51649300	-0.83967100
H	-5.60220400	-3.92617400	-2.46745300
H	-8.01920400	-2.00567900	-2.33081200
H	-8.20346100	-3.66716600	-2.92391100
H	-9.23595300	-3.06138900	-1.61550500
C	1.45576000	-6.02415600	3.15073400
O	0.52511100	-6.40719600	3.85941800
N	1.54044200	-4.74297100	2.67327400
C	0.33685400	-3.92463500	2.68886400
C	-0.58900300	-4.32371600	1.53044200
S	-2.34437500	-3.83824200	1.71340900
O	4.97850500	-3.43204900	-3.20625200
C	-1.64054100	-0.39517700	-0.52450200
C	-1.53503500	-1.49225900	0.38722100
C	-2.63338700	-2.27266500	0.77873300
O	-3.85179000	-2.02988700	0.64040400
C	-0.81121400	0.69885600	-0.46420600
C	-2.84742200	1.75117200	0.71802000
O	-3.31279100	2.36756400	-0.17376000
O	-2.67347800	1.31354700	1.80212700
C	-2.67973500	-0.44826500	-1.62195000
H	-3.66975000	-0.63723200	-1.20693400
H	-2.69013200	0.47555800	-2.20357500
H	-2.46849700	-1.28256100	-2.29905400
H	-0.04801400	0.77418400	0.30311000
H	-0.59960800	-1.63691400	0.91563200
H	-0.77797200	1.42949800	-1.26592400
H	-0.61774200	-5.41502900	1.48347600
H	-0.19693400	-3.95756200	0.57963200
H	-0.17410200	-4.09030500	3.63857800
H	0.62050100	-2.87257400	2.62391800
H	2.21886300	-4.53918600	1.93981700
C	2.56507100	-6.95484600	2.71088000
H	2.35914400	-7.29993700	1.69036500
H	3.54282700	-6.46501100	2.66705500
H	2.59924100	-7.81543900	3.37957000
H	-0.51894100	-4.94670000	-6.21884400
H	1.22746000	-5.05780700	-5.73381800
H	5.92224700	-6.95449100	-2.67757600
H	8.31984800	-1.62301300	-4.45686000
H	9.85494600	-2.33640700	-3.78266600
H	8.39611600	0.20631900	-4.12281100
H	8.65482200	1.80523000	-4.94114000
H	0.85064100	2.57056600	7.37393500
H	2.62213300	2.61773900	7.50753300
H	6.85031500	6.62739200	-0.35308500
H	5.03482200	6.72610100	-0.28056300
H	3.35914900	9.22357400	-1.97319800
H	4.12159300	7.65638100	-1.60403500
H	3.27584600	8.73395200	2.81269200
H	4.33429300	8.07506500	4.12649300
H	-2.10204300	8.28017100	3.52326100
H	-2.42636300	7.26178800	2.09547700
H	-5.10552500	5.59074900	-5.04323000
H	-6.50179500	4.81649200	-4.20802500
H	-8.47346100	0.51265000	-5.80784100
H	-7.91067600	1.97665300	-6.70337400
H	-11.20224100	0.44344700	-4.16732900
H	-9.46953600	0.94644500	-4.30831000
H	-8.92919500	-4.74768900	0.93873900
H	-8.81340000	-6.38776000	0.18224100
H	-4.30959000	-5.36591600	4.03076000
H	-6.09639500	-5.18908900	3.96153000
H	-3.25540800	-2.38681600	8.24567000
H	-1.49834700	-2.11516300	8.41522200
H	0.50118900	0.18569800	-6.52674200
H	0.70820200	1.88835100	-5.90920200
H	4.02375300	-3.41925800	-3.01422200
H	5.23750700	-2.48301500	-3.26542900
O	-3.26313400	2.91064300	4.12620600
H	-2.97128400	2.35776400	3.38456100
H	-3.80462100	2.29481000	4.65532400
O	4.56968000	1.04509100	-5.33994200
H	4.94784900	0.36941300	-4.73451800
H	3.62085800	0.86525100	-5.25664600
O	-4.22304100	4.63863800	2.08822300
H	-4.05852800	4.09095200	2.88262400

Int$_{dpGlu72}$ (+2.3)

C	3.36371600	8.20224300	-1.43264000
C	2.53678600	7.59800000	-2.58204200
C	2.32306200	6.10256300	-2.48126100
C	1.34814300	5.57204300	-1.62681400
C	3.08824200	5.21595200	-3.25103600
C	1.13423100	4.19578000	-1.56162800
C	2.88516000	3.83498100	-3.18881000
C	1.89934600	3.32658400	-2.34185000
H	2.83948400	8.12692500	-0.47633000
H	1.56163700	8.10045800	-2.62434900
H	3.03652800	7.81688300	-3.53346100
H	0.76419200	6.24078500	-1.00077000
H	3.84792300	5.61559900	-3.91890300
H	0.37122900	3.79712900	-0.90092400
H	3.48002200	3.16106200	-3.80068900
H	1.73996200	2.25824200	-2.27802800
C	6.03635100	6.02782600	0.21748200
C	5.85861300	4.66874100	-0.45923700
C	4.64253500	3.91424400	0.07778500
S	4.80235900	3.31265500	1.80725700
C	6.02271500	1.96813200	1.59927900
H	6.05461000	5.89195600	1.30483300
H	5.70844800	4.80261800	-1.53859500
H	6.76180700	4.05644400	-0.35033900
H	3.76927100	4.57040700	0.09858100
H	4.40215800	3.06048600	-0.55414400
H	6.05126800	1.42223900	2.54470700
H	5.73261700	1.27342200	0.80861800
H	7.02118100	2.35614100	1.38471500
C	-1.92860600	7.30092000	3.29704300
C	-0.50906300	6.78978900	3.47463500
O	0.23976500	7.32155400	4.30618200
H	-2.61057000	6.65217400	3.86041800
N	-0.11932100	5.72434100	2.73441100
C	1.20271700	5.10676100	2.97781400
C	2.35600900	6.05955700	2.59506800
O	2.70359200	6.23462500	1.42953500
C	1.17322800	3.83702900	2.11740700
C	-0.31581700	3.48740100	2.02513800
C	-0.99712000	4.85491700	1.92456600

H	1.28069800	4.86657100	4.04539100
H	1.57658100	4.08047500	1.13204900
H	1.78970000	3.04394200	2.54276900
H	-0.54877600	2.84409000	1.17405800
H	-0.67081000	2.98132100	2.92725400
H	-1.03433100	5.21511100	0.88754300
H	-2.00950300	4.82293400	2.33080900
N	2.96043400	6.64869600	3.66418800
C	3.93178900	7.71993900	3.46981800
H	2.32389400	6.76259200	4.44637300
H	4.65723500	7.40949800	2.71715300
C	1.65456000	1.81115800	7.26978600
C	1.69928500	1.50838700	5.75395500
C	2.58869100	0.35338200	5.35764300
C	2.07976200	-0.95377200	5.35858300
C	3.92078300	0.55261400	4.97595300
C	2.87899400	-2.03426700	4.98674900
C	4.72663500	-0.52821000	4.61337600
C	4.20885100	-1.82370100	4.61835600
H	1.59241500	0.88857500	7.85458500
H	0.67888000	1.28446900	5.42185200
H	2.00385000	2.41333900	5.21507500
H	1.04009100	-1.11948300	5.63115800
H	4.32090700	1.56308200	4.94321400
H	2.45757500	-3.03460800	4.95068900
H	5.75587700	-0.35744000	4.31051100
H	4.82727600	-2.65976700	4.31161300
C	0.06782500	-4.78184000	-5.53972900
C	-0.07694500	-3.57728100	-4.63601300
O	-0.05258300	-2.43374600	-5.08923600
H	-0.17079800	-5.71666800	-5.02358400
N	-0.26554900	-3.86021900	-3.31483300
C	-0.02760400	-2.83132500	-2.33050100
C	1.45206000	-2.81886900	-1.94824400
O	2.24614900	-3.55577300	-2.54265900
H	0.01802100	-4.78721200	-3.02317300
H	-0.28095800	-1.86632700	-2.77018300
H	-0.66997700	-2.95797600	-1.45620800
N	1.80637800	-1.98340400	-0.95842100
C	3.20406700	-1.76532700	-0.62172700
C	3.84870500	-3.03879600	-0.05826100
O	3.19376500	-3.98482000	0.41255800
C	3.34854400	-0.60663900	0.39169900
C	3.06088400	0.73159000	-0.28760600
C	2.50316900	-0.81826600	1.65324700
H	1.13642400	-1.27092500	-0.69825500
H	3.75237500	-1.49587000	-1.53087200
H	4.40120500	-0.61407300	0.68918400
H	3.06869600	1.54244700	0.44484200
H	3.80502800	0.94201100	-1.05806300
H	2.09005700	0.72884200	-0.78143400
H	2.75177300	-0.06347000	2.40293700
H	1.43005400	-0.73786400	1.45288500
H	2.68404500	-1.79987600	2.09520400
N	5.18730000	-2.99876900	-0.08782900
C	6.02901200	-4.02912500	0.51092100
C	5.68905200	-5.43742400	-0.01312300
O	5.71651700	-6.41817600	0.73170600
C	5.98144300	-3.98317500	2.03646400
H	5.64927600	-2.21741200	-0.57371400
H	7.03911800	-3.81210500	0.14701000
H	6.60829100	-4.77256900	2.45431800
H	6.32929700	-3.00755100	2.38865400
H	4.95654000	-4.14415200	2.37823900
N	5.45188900	-5.49091700	-1.34463600
C	5.01884000	-6.71608500	-1.98652900
C	3.57360100	-6.58605500	-2.48660100
O	2.64435100	-6.38051000	-1.43615600
H	5.40714500	-4.62760700	-1.89945900
H	5.07954800	-7.50621300	-1.23440300
H	3.51688500	-5.77714700	-3.22896100
H	3.28104100	-7.51981600	-2.98446700
H	2.86831100	-5.53966500	-0.99953300
C	0.20443000	1.19378800	-5.80061400
C	0.19569100	0.62823800	-4.38536500
S	1.84655800	0.03956200	-3.82657000
H	-0.82371100	1.46031900	-6.08415600
H	-0.10698000	1.38784400	-3.66110000
H	-0.48813200	-0.21706300	-4.30791800
H	1.75954400	-1.16753100	-4.41980200
C	8.80635700	-2.59464500	-4.18871400
C	8.01891400	-3.49297100	-3.22813100
O	8.03376100	-2.98869300	-1.89791700
H	8.96382100	-3.09624800	-5.15240000
H	6.99985300	-3.65741100	-3.58843000
H	8.50735100	-4.47492600	-3.17588800
H	7.47884100	-2.18354800	-1.84015500
C	7.99049600	1.14466300	-4.23853100
C	7.57272700	1.83074700	-2.92292300
C	6.20688200	1.38185400	-2.36623300
C	6.04514600	-0.13820600	-2.29886300
O	6.46868200	-0.73062500	-1.26016100
O	5.51452100	-0.71031800	-3.29977300
H	7.13247100	1.01533100	-4.90244200
H	8.34480700	1.65068600	-2.16420400
H	7.52438300	2.91788100	-3.06399200
H	6.09167700	1.79017400	-1.36051100
H	5.42031400	1.78342800	-3.00741700
C	-5.26306600	5.27218800	-4.17069200
C	-4.72541800	5.67108300	-2.78372700
C	-3.20681500	5.41176300	-2.64859800
C	-5.51954600	4.98450700	-1.66600200
C	-2.78985900	3.93904700	-2.53214100
H	-4.90460200	4.27271300	-4.44516700
H	-4.87187100	6.75570500	-2.67365900
H	-2.69347500	5.86587600	-3.50665800
H	-2.84175100	5.94578300	-1.76112800
H	-5.11419700	5.22821100	-0.67803500
H	-6.57152300	5.28915300	-1.68457100
H	-5.49601000	3.89679600	-1.76533400
H	-1.69855900	3.85599900	-2.52509500
H	-3.16021200	3.47963800	-1.61006100
H	-3.15741200	3.33885400	-3.37162400
C	-7.61165600	1.43078200	-6.51809200
C	-6.30070100	1.31295000	-5.72320500
C	-6.11627700	-0.02510300	-4.98694400
C	-6.18895800	-1.23145400	-5.93006800
C	-4.80034100	-0.03195400	-4.20448300
H	-7.56924900	0.86830900	-7.45645400
H	-5.44241600	1.48375100	-6.38860600

H	-6.26535100	2.11498100	-4.97420300
H	-6.93710600	-0.11300900	-4.26078300
H	-6.05389000	-2.16373800	-5.37241800
H	-7.15113100	-1.29083400	-6.44902100
H	-5.39844800	-1.17886500	-6.68942100
H	-4.70346200	-0.93700200	-3.59893600
H	-3.93870300	0.01275800	-4.88204400
H	-4.73696500	0.82391700	-3.52682800
C	-10.23110200	1.04132600	-3.78075800
C	-10.24782300	1.78137900	-2.43103100
C	-9.03838600	1.38090800	-1.60221700
C	-9.17131800	0.48641000	-0.53241200
C	-7.74853300	1.81206600	-1.94650500
C	-8.05581600	0.01378500	0.16372600
C	-6.63019900	1.34212300	-1.25525600
C	-6.77705100	0.43251300	-0.20652500
H	-9.95082600	-0.00158500	-3.58157700
H	-10.26435900	2.86666800	-2.59324800
H	-11.15997600	1.53086900	-1.87769400
H	-10.16340300	0.13788800	-0.25434400
H	-7.61800300	2.51567700	-2.76579200
H	-8.17189300	-0.70046400	0.97435800
H	-5.63442200	1.67631300	-1.52708400
H	-5.89942600	0.04166000	0.29665400
C	-2.52878100	-2.68696900	7.68671400
C	-2.51329600	-2.15692900	6.26140700
O	-2.90311100	-2.84847700	5.32561800
H	-2.40560700	-3.76988000	7.65569700
N	-2.07935300	-0.86829700	6.10576200
C	-2.42267600	-0.10588100	4.88843900
C	-3.95635300	-0.19725800	4.71427000
O	-4.67754600	0.12761900	5.66321700
C	-1.56356700	-0.49772000	3.69411400
H	-2.04356100	-0.32191600	6.95390300
H	-2.23688000	0.94546300	5.11897400
H	-1.81689100	0.12196700	2.83235000
H	-0.50712000	-0.33721300	3.92507300
H	-1.70280500	-1.54618000	3.43289000
N	-4.43096400	-0.64904000	3.53568800
C	-5.83103400	-0.93866400	3.32276300
C	-6.12503500	-2.41765200	3.04056200
O	-7.15685700	-2.73773800	2.43942100
H	-3.82794000	-0.88684300	2.75067000
H	-6.37039400	-0.63669900	4.22455000
H	-6.22508500	-0.36881000	2.47930300
N	-5.24045700	-3.30367200	3.54069800
C	-5.39597900	-4.72857100	3.30987000
H	-4.38015000	-2.97608500	3.96765700
H	-5.37421500	-4.95299600	2.23832400
C	-8.93589800	-4.97500900	-0.27506200
C	-7.50829500	-4.67139700	-0.75515900
C	-7.20479800	-3.18734500	-1.02178400
C	-5.71085400	-2.98162400	-1.29478900
C	-8.04021700	-2.61500600	-2.17457600
H	-9.68212100	-4.65414300	-1.01145300
H	-6.80454900	-5.02796400	0.00689500
H	-7.29551000	-5.24980800	-1.66749000
H	-7.44831100	-2.64042000	-0.10451900
H	-5.49193500	-1.92647100	-1.48166900
H	-5.09910800	-3.28539500	-0.44172100
H	-5.39036700	-3.55547700	-2.17535100
H	-7.79750500	-1.56075100	-2.33797500
H	-7.83671800	-3.15801700	-3.10781000
H	-9.11633500	-2.67568900	-1.97938600
C	1.11401200	-6.10093800	2.89163100
O	0.14464900	-6.48834500	3.54528500
N	1.22138700	-4.81689200	2.43158200
C	0.06458400	-3.93383800	2.50258900
C	-0.94612300	-4.25770000	1.39273600
S	-2.59838500	-3.49849200	1.60758900
O	4.88717400	-3.40622500	-3.26105900
C	-1.41517200	-0.15822700	-0.58575000
C	-1.42378200	-1.32207700	0.28676100
C	-2.58267200	-1.85466800	0.81395700
O	-3.73658000	-1.30024500	0.87472100
C	-0.46195900	0.80572300	-0.52395000
C	-2.47827500	-0.08000900	-1.66300000
H	-3.46548600	-0.27674000	-1.24442100
H	-2.48249000	0.89585000	-2.15588300
H	-2.30017200	-0.84935100	-2.42272200
H	0.28902600	0.80747400	0.25624400
H	-0.47654800	-1.74257200	0.60013500
H	-0.40023900	1.59273900	-1.26875200
H	-1.14142500	-5.33263300	1.41905500
H	-0.54097800	-4.01187700	0.40835400
H	-0.41773100	-4.07295800	3.47203200
H	0.40687800	-2.89882600	2.43262100
H	1.94489600	-4.58549800	1.75235900
C	2.26060000	-7.03005200	2.52958500
H	2.21024800	-7.28038800	1.46298900
H	3.23943400	-6.56408300	2.67954100
H	2.17744300	-7.94017500	3.12335700
H	-0.62548800	-4.66749400	-6.37957200
H	1.10924100	-4.84400900	-5.87261000
H	5.69960400	-6.96718000	-2.80664300
H	8.27671100	-1.65734100	-4.38856200
H	9.78005600	-2.43502300	-3.71384000
H	8.40049400	0.15816000	-3.99882200
H	8.71750300	1.77258800	-4.76421200
H	0.74859300	2.39819300	7.45291400
H	2.51843600	2.38960500	7.61376200
H	6.98146600	6.50653900	-0.05940800
H	5.16879200	6.65605100	-0.01049300
H	3.59203900	9.25067500	-1.65111800
H	4.30321000	7.65150000	-1.31840700
H	3.42044000	8.62131400	3.11605200
H	4.43900500	7.89295300	4.42468800
H	-1.97864700	8.30380000	3.73375300
H	-2.30991700	7.33829400	2.27124800
H	-4.92494500	5.95966400	-4.95317600
H	-6.35569300	5.20200700	-4.16228600
H	-8.42522700	1.00711900	-5.92021000
H	-7.80674700	2.48009100	-6.76272600
H	-11.17998500	0.96879400	-4.32262800
H	-9.43154100	1.42492400	-4.42297600
H	-9.13658200	-4.43635500	0.65688200
H	-9.05628600	-6.05583500	-0.14709000
H	-4.58520000	-5.28109500	3.79607700
H	-6.36487600	-5.05008000	3.70612200
H	-3.51131500	-2.46092800	8.11409900

H	-1.75003100	-2.24588900	8.31758500
H	0.54651100	0.43986600	-6.51704300
H	0.79278300	2.11649200	-5.84541100
H	3.93360000	-3.39139400	-3.06378700
H	5.16234100	-2.46057600	-3.27311200
O	-3.16206400	2.65927600	3.47437600
H	-3.28445500	2.19617500	2.61147700
H	-3.95932100	2.43740800	3.97155700
O	4.63413300	1.19344300	-5.18347600
H	4.96986900	0.48080000	-4.59494800
H	3.67891000	1.03323200	-5.15167700
O	-3.41177300	1.42256300	1.02170000
H	-3.59863500	0.44892000	0.98517200

TS2$_{dpGlu72}$ (+18.9)

C	3.22127600	8.24137700	-1.51838700
C	2.37239900	7.65002900	-2.65415200
C	2.14989100	6.16075300	-2.54396600
C	1.17069100	5.64422500	-1.68755100
C	2.92023200	5.26749100	-3.29960000
C	0.95353900	4.26891700	-1.61143500
C	2.70960800	3.88998400	-3.22702500
C	1.71604500	3.39143500	-2.38312900
H	2.71583900	8.15908800	-0.55258500
H	1.40255900	8.16426600	-2.67855100
H	2.85891600	7.86550200	-3.61332000
H	0.58365900	6.32397000	-1.07535000
H	3.68336800	5.66213600	-3.96713200
H	0.18208000	3.87950400	-0.95392800
H	3.29460900	3.20141500	-3.83226800
H	1.54392100	2.32252300	-2.36625300
C	5.95395500	6.10275200	0.07902500
C	5.77560000	4.74356600	-0.59397100
C	4.58920500	3.97527400	-0.01498500
S	4.81506500	3.40981100	1.72069800
C	6.09322100	2.12166200	1.50294400
H	5.99712300	5.96722400	1.16560800
H	5.58546800	4.87442800	-1.66709100
H	6.68974900	4.14318500	-0.51653000
H	3.70522300	4.61640900	0.02048000
H	4.34864800	3.10939300	-0.62883800
H	6.22601600	1.64368500	2.47596500
H	5.79018800	1.36189300	0.77994100
H	7.04738200	2.55186200	1.18971300
C	-1.96472400	7.27367400	3.31484400
C	-0.53770000	6.77195700	3.46356500
O	0.21749800	7.29714600	4.29286600
H	-2.62902600	6.61847000	3.89200500
N	-0.14646900	5.72088200	2.70188500
C	1.19014600	5.12114500	2.90974600
C	2.32137000	6.09764900	2.51875700
O	2.64266500	6.29104700	1.34937100
C	1.16767600	3.86846600	2.02439400
C	-0.31691500	3.49806800	1.93933100
C	-1.02035700	4.85885400	1.88103800
H	1.29281100	4.86414800	3.97126300
H	1.55159800	4.13757400	1.03810800
H	1.80073600	3.07595300	2.42598100
H	-0.54049700	2.87709300	1.06779900
H	-0.65509300	2.96142700	2.83031300
H	-1.07435000	5.24174500	0.85342300
H	-2.02704200	4.80451800	2.29936800
N	2.93886800	6.68398000	3.58200500
C	3.89202400	7.76881400	3.37205000
H	2.31901500	6.78139600	4.37947100
H	4.60700800	7.46877600	2.60531800
C	1.76711100	1.83278000	7.21730300
C	1.79038900	1.52506000	5.70309100
C	2.69171900	0.38501600	5.28836300
C	2.21653800	-0.93449500	5.32611200
C	3.99649300	0.61237800	4.83517600
C	3.01795200	-1.99791800	4.91167600
C	4.80498400	-0.45110000	4.42902600
C	4.31796300	-1.75788100	4.46265100
H	1.72909000	0.91120700	7.80612100
H	0.76755200	1.28318700	5.39074700
H	2.06832200	2.43296300	5.15513400
H	1.19732800	-1.12348000	5.65575500
H	4.36916300	1.63170500	4.77128800
H	2.61897300	-3.00808800	4.90008900
H	5.80927900	-0.25746100	4.06354800
H	4.93414800	-2.57914400	4.11349000
C	0.01401100	-4.78627400	-5.55698300
C	-0.28233000	-3.68833500	-4.55360200
O	-0.85779700	-2.65089000	-4.86435300
H	-0.17919100	-5.76003500	-5.08890800
N	0.06904600	-4.00844100	-3.27385900
C	0.15782400	-2.98689200	-2.26105700
C	1.59666800	-2.92165700	-1.76878700
O	2.43503900	-3.72620900	-2.19553000
H	0.72551200	-4.77079300	-3.14396600
H	-0.10077900	-2.02705900	-2.71295500
H	-0.53221300	-3.16730100	-1.43098500
N	1.90307500	-1.98810300	-0.85347600
C	3.29484200	-1.72011500	-0.51062900
C	3.97755400	-2.96278300	0.07126600
O	3.37149800	-3.84655800	0.69571500
C	3.39476000	-0.53954300	0.48063300
C	3.19768100	0.78594400	-0.25343600
C	2.45129300	-0.68964900	1.67801200
H	1.23681900	-1.24084400	-0.72355600
H	3.83475800	-1.45016400	-1.42418600
H	4.42136900	-0.56458400	0.86059300
H	3.11898100	1.60971700	0.45901100
H	4.03613400	0.97774800	-0.92292400
H	2.30581500	0.78226300	-0.87659100
H	2.69886000	0.04818200	2.44349400
H	1.40515300	-0.52683600	1.39980100
H	2.53299400	-1.68154100	2.12418800
N	5.30403300	-2.95768700	-0.12445200
C	6.18388800	-4.00155600	0.38735500
C	5.79069600	-5.39730700	-0.13818600
O	5.86682100	-6.39345900	0.58312700
C	6.27780300	-3.98646800	1.91187800
H	5.72442700	-2.19655600	-0.67524000
H	7.16067100	-3.78528100	-0.05925000
H	6.93855600	-4.78517400	2.25292800
H	6.66450900	-3.01931000	2.24803800
H	5.28933800	-4.15105800	2.34499800

N	5.45804000	-5.42480800	-1.45132700
C	5.05867700	-6.65443800	-2.10154300
C	3.61776500	-6.59651100	-2.61981900
O	2.65813500	-6.44090300	-1.58776000
H	5.40364600	-4.55594600	-1.99628800
H	5.14663400	-7.44644300	-1.35385000
H	3.53023700	-5.78996300	-3.36164900
H	3.38841000	-7.54406100	-3.12464100
H	2.76488100	-5.53400200	-1.25662200
C	0.06758400	1.19052500	-5.82099600
C	-0.15935300	0.62441400	-4.42397200
S	1.33037900	0.05430200	-3.51417500
H	-0.93057800	1.47714300	-6.19602300
H	-0.65295900	1.38918600	-3.81114200
H	-0.83650700	-0.22993300	-4.50879600
H	0.47100900	0.27999000	-2.07495600
C	8.74812900	-2.48528300	-4.37874100
C	8.00473400	-3.40067200	-3.40021100
O	8.06624700	-2.91053500	-2.06589500
H	8.89363700	-2.98417900	-5.34589000
H	6.97536300	-3.57371200	-3.72276200
H	8.50690500	-4.37712300	-3.37750200
H	7.50655300	-2.11050700	-1.97709800
C	7.88269800	1.24318300	-4.41400300
C	7.49373100	1.92590400	-3.08886600
C	6.17495700	1.42609000	-2.46737900
C	6.07076500	-0.09953900	-2.39618200
O	6.51120100	-0.67764600	-1.35463900
O	5.56456300	-0.68806700	-3.39741600
H	7.00547700	1.09528900	-5.04946100
H	8.30772300	1.78691400	-2.36556600
H	7.39472600	3.00874600	-3.23826200
H	6.09460900	1.83344600	-1.45741900
H	5.34051000	1.79071700	-3.07019900
C	-5.41864500	5.19663200	-4.08551900
C	-4.85445000	5.61093100	-2.71359100
C	-3.33134500	5.36377600	-2.61276900
C	-5.61877100	4.93247000	-1.57058700
C	-2.90171300	3.89502300	-2.49652400
H	-5.05262300	4.20212600	-4.36687400
H	-5.00578200	6.69555200	-2.61066400
H	-2.84191900	5.81355800	-3.48649700
H	-2.94755500	5.90740100	-1.73911100
H	-5.19361400	5.19228300	-0.59492800
H	-6.67300100	5.23007600	-1.56988300
H	-5.59079300	3.84378600	-1.65648700
H	-1.81057000	3.81883800	-2.52258200
H	-3.24504800	3.44485200	-1.55893100
H	-3.28918400	3.28479600	-3.31935400
C	-7.76338000	1.32574300	-6.38507100
C	-6.43181400	1.21504500	-5.62689800
C	-6.20650600	-0.13743200	-4.92966800
C	-6.27967900	-1.32090500	-5.90141300
C	-4.87153600	-0.13962300	-4.18085500
H	-7.73897100	0.76603500	-7.32586200
H	-5.59418600	1.41560800	-6.30968200
H	-6.39057100	2.00021400	-4.86034400
H	-7.00860600	-0.25830200	-4.18688000
H	-6.10807500	-2.26374900	-5.37234700
H	-7.25430000	-1.38887200	-6.39553100

H	-5.51098800	-1.23210900	-6.67908000
H	-4.74286900	-1.05857800	-3.60278600
H	-4.02682700	-0.06337500	-4.87577500
H	-4.80892400	0.70114400	-3.48425500
C	-10.32314300	0.90342600	-3.59671800
C	-10.32442800	1.64914500	-2.25062800
C	-9.08383600	1.27764600	-1.45660500
C	-9.16601200	0.38963200	-0.37665700
C	-7.81405500	1.72473700	-1.85094000
C	-8.01961400	-0.06089200	0.28260800
C	-6.66502900	1.27611600	-1.19813200
C	-6.76226400	0.37405900	-0.13756000
H	-10.02529000	-0.13500800	-3.40036700
H	-10.36881600	2.73302300	-2.41653300
H	-11.21564700	1.38074800	-1.67224100
H	-10.14209800	0.02774100	-0.06195200
H	-7.72306100	2.42127600	-2.68121300
H	-8.09599500	-0.77092600	1.10144200
H	-5.68574500	1.61954900	-1.51391200
H	-5.86230700	0.00244500	0.33901700
C	-2.34856400	-2.71909700	7.71762600
C	-2.36421200	-2.17868800	6.29587700
O	-2.75597000	-2.86621700	5.35638000
H	-2.21136400	-3.79988800	7.67960700
N	-1.95606800	-0.88270300	6.14699200
C	-2.30055100	-0.10961700	4.93889400
C	-3.83237200	-0.20556700	4.75382800
O	-4.56623200	0.16116300	5.67515900
C	-1.43401600	-0.47985800	3.74196700
H	-1.88291500	-0.34501600	6.99786100
H	-2.12428600	0.93991100	5.18179500
H	-1.70154600	0.14290100	2.88573200
H	-0.38057700	-0.30215400	3.97175000
H	-1.55289900	-1.53083600	3.47977800
N	-4.29234800	-0.71264700	3.59024500
C	-5.68975700	-1.00354700	3.35925500
C	-5.99079000	-2.48578000	3.10179300
O	-7.02126000	-2.80734200	2.50177700
H	-3.66403500	-0.97602400	2.84233200
H	-6.24013600	-0.67828700	4.24604100
H	-6.06909300	-0.44931500	2.49898500
N	-5.11090800	-3.37174300	3.61194500
C	-5.27479000	-4.79969400	3.39855800
H	-4.26436600	-3.04411100	4.06627400
H	-5.27516500	-5.03042800	2.32801800
C	-8.88096300	-5.09356700	-0.11568400
C	-7.47249100	-4.76521200	-0.63204700
C	-7.20924100	-3.27641200	-0.91474800
C	-5.72654300	-3.03940800	-1.22131100
C	-8.08021900	-2.72901700	-2.05310800
H	-9.64978300	-4.78527000	-0.83398900
H	-6.74257100	-5.10227900	0.11426500
H	-7.27006600	-5.34434200	-1.54586100
H	-7.44630900	-2.72808300	0.00323500
H	-5.53829200	-1.98130900	-1.42525100
H	-5.09132200	-3.32384500	-0.37823600
H	-5.40924000	-3.61304000	-2.10235100
H	-7.86585200	-1.66985000	-2.22433600
H	-7.88256400	-3.26985600	-2.98844400
H	-9.15023700	-2.81447100	-1.83554000

C	1.23987600	-6.10130700	2.86895400
O	0.23109900	-6.53969600	3.42322900
N	1.38353100	-4.77723800	2.55035800
C	0.22250400	-3.90560900	2.62407500
C	-0.67405200	-4.10208500	1.39243000
S	-2.37610700	-3.44770300	1.58638400
O	4.88825400	-3.34731700	-3.36573700
C	-1.41690700	-0.16961800	-0.72518500
C	-1.35061000	-1.18360900	0.23787600
C	-2.48511200	-1.84038800	0.78372400
O	-3.64914000	-1.35649300	0.84937200
C	-0.32825700	0.70686200	-1.00489700
C	-2.59294700	-0.08942900	-1.67152800
H	-2.83685600	0.94730900	-1.91835300
H	-2.30210100	-0.57960200	-2.60851900
H	-3.47924700	-0.58820000	-1.28926100
H	-0.61284300	1.64349500	-1.48532700
H	-0.39140300	-1.42630200	0.68086600
H	0.38977500	0.85363600	-0.20050900
H	-0.81545300	-5.17392400	1.23677300
H	-0.20847000	-3.68743100	0.49827300
H	-0.33958500	-4.15454700	3.52623300
H	0.55851500	-2.86838900	2.69875600
H	2.13374900	-4.49368400	1.91884700
C	2.39423000	-7.00200900	2.46812700
H	2.29486900	-7.26322100	1.40728100
H	3.36770700	-6.51329700	2.56644600
H	2.35869600	-7.91304100	3.06572800
H	-0.69729400	-4.68133500	-6.38288100
H	1.04924600	-4.83504900	-5.91017200
H	5.72632700	-6.89706200	-2.93486000
H	8.20282600	-1.55496500	-4.56865100
H	9.72901100	-2.31275300	-3.92340000
H	8.31035000	0.26221800	-4.18089200
H	8.59158900	1.88015500	-4.95328600
H	0.85728500	2.40817800	7.41820000
H	2.62991400	2.42256600	7.54423200
H	6.88710300	6.59347700	-0.21626900
H	5.07381200	6.71940900	-0.13152500
H	3.43155200	9.29263000	-1.74153300
H	4.16994400	7.70306200	-1.42254500
H	3.36214200	8.66334100	3.02822700
H	4.41566000	7.94890600	4.31671000
H	-2.01915200	8.27610700	3.75231100
H	-2.36660100	7.30571100	2.29682000
H	-5.10585800	5.89047000	-4.87389200
H	-6.51030500	5.11487900	-4.05551500
H	-8.55946400	0.89193000	-5.77108900
H	-7.97688500	2.37227300	-6.62607400
H	-11.28143400	0.81863800	-4.11959700
H	-9.54122100	1.29719700	-4.25439600
H	-9.07040500	-4.55736700	0.81977900
H	-8.98482000	-6.17606400	0.01476600
H	-4.44736300	-5.34142300	3.86896200
H	-6.23129300	-5.13366200	3.81403000
H	-3.32533500	-2.50553100	8.16442300
H	-1.56330500	-2.26758000	8.33297600
H	0.40556200	0.44071600	-6.54488900
H	0.64322100	2.12072600	-5.87858200
H	3.95391200	-3.34547800	-3.10204700
H	5.15930300	-2.39839000	-3.37646300
O	4.12452700	1.17436100	-4.98299000
H	4.64727200	0.47216000	-4.54068900
H	3.22352400	0.97802500	-4.67087600
O	-3.11266300	2.60939100	3.52907500
H	-3.26333200	2.16403800	2.66544700
H	-3.90000300	2.39753200	4.04636100
O	-3.37922700	1.41052700	1.03245500
H	-3.55265500	0.44242100	0.96253100

E:P$_{dpGlu72}$ (+12.1)

C	3.21092400	8.21758000	-1.59082400
C	2.37344300	7.60798700	-2.72568700
C	2.14512600	6.12015400	-2.59758300
C	1.16561000	5.61889200	-1.73276600
C	2.90295100	5.21236700	-3.34949300
C	0.93308000	4.24608100	-1.64911200
C	2.67463500	3.83775700	-3.26951200
C	1.67642500	3.35305800	-2.42225900
H	2.69968300	8.14412700	-0.62730700
H	1.40460100	8.12330100	-2.76920300
H	2.87064000	7.80831500	-3.68264900
H	0.58886900	6.30852900	-1.12135100
H	3.66814800	5.59376800	-4.02256000
H	0.15948800	3.87371000	-0.98384200
H	3.24673500	3.13588400	-3.87199200
H	1.49769700	2.28149600	-2.41570000
C	5.94169600	6.10067700	0.03785700
C	5.76584200	4.73894200	-0.62735200
C	4.58012300	3.97964700	-0.03922300
S	4.80961000	3.44651500	1.70675800
C	6.09403300	2.16178000	1.50580900
H	5.98113000	5.97173400	1.12529900
H	5.57352500	4.86181500	-1.70093000
H	6.67926500	4.13797800	-0.54712000
H	3.69472300	4.61926100	-0.01514500
H	4.34283100	3.10389000	-0.63866000
H	6.22886400	1.69439300	2.48370300
H	5.79528900	1.39426600	0.78935400
H	7.04617800	2.59370700	1.18918500
C	-1.99260400	7.28801400	3.23110000
C	-0.56496400	6.79526600	3.39623500
O	0.18387100	7.33501500	4.22141600
H	-2.65801700	6.63755800	3.81250900
N	-0.16842200	5.73321800	2.65369800
C	1.16662800	5.13592100	2.87599400
C	2.29859100	6.10682700	2.47247000
O	2.62217400	6.28117800	1.30141900
C	1.14376800	3.86797000	2.01240200
C	-0.34235600	3.49886400	1.93713300
C	-1.04098300	4.85967300	1.84663200
H	1.26532500	4.89655700	3.94209000
H	1.52554500	4.11879700	1.02026800
H	1.77711900	3.08239900	2.42691900
H	-0.56837600	2.86274000	1.07828000
H	-0.68176000	2.98368200	2.84078400
H	-1.08800800	5.22023400	0.81068600
H	-2.05020400	4.82048200	2.26083200
N	2.91064100	6.71159100	3.52852500

C	3.86302100	7.79520300	3.30672700
H	2.28839400	6.82007800	4.32253000
H	4.58058700	7.48673800	2.54589900
C	1.73402800	1.89406200	7.20303200
C	1.76254900	1.57847100	5.69040700
C	2.65796900	0.43209800	5.28063800
C	2.18519900	-0.88707400	5.34989400
C	3.95193600	0.65315800	4.79531100
C	2.97758900	-1.95625500	4.93326000
C	4.75147500	-0.41601200	4.38660400
C	4.26636200	-1.72203500	4.45011700
H	1.69618800	0.97646000	7.79796400
H	0.73892900	1.34194800	5.37523200
H	2.04675800	2.48246200	5.13945500
H	1.17445300	-1.07168800	5.70776800
H	4.32183700	1.67150200	4.70618300
H	2.58066800	-2.96722200	4.94660700
H	5.74571600	-0.22751300	3.99265200
H	4.87410100	-2.54728800	4.09667100
C	0.04534300	-4.85556200	-5.51112500
C	-0.25555300	-3.73740300	-4.53369200
O	-0.92086900	-2.75418900	-4.84147700
H	-0.14855800	-5.81955100	-5.02342500
N	0.21008300	-3.97188300	-3.27600900
C	0.29091000	-2.90777600	-2.31021600
C	1.67953500	-2.92112400	-1.69875200
O	2.45101700	-3.87001800	-1.89851100
H	0.88746700	-4.71300000	-3.13155300
H	0.15942200	-1.94437300	-2.81702100
H	-0.46985800	-2.99881200	-1.52559900
N	1.99153700	-1.85052400	-0.94576600
C	3.36418000	-1.62275000	-0.49587200
C	3.98997800	-2.86291700	0.14611400
O	3.35725800	-3.65501800	0.86003900
C	3.39522900	-0.44501900	0.50363300
C	3.15752900	0.87316200	-0.22794400
C	2.41634100	-0.63173500	1.66478300
H	1.53337100	-1.00494300	-1.29017800
H	3.97038500	-1.35265800	-1.36529300
H	4.41237400	-0.43172600	0.91122300
H	3.11086300	1.69616800	0.48715300
H	3.95645600	1.06589500	-0.94457500
H	2.23515900	0.86765800	-0.80670100
H	2.57752100	0.13794600	2.42268800
H	1.38310600	-0.54853700	1.31684600
H	2.53824500	-1.60803700	2.13345800
N	5.30981500	-2.94066600	-0.08254600
C	6.16043800	-3.99400800	0.45646800
C	5.74934500	-5.39202500	-0.05208200
O	5.79086000	-6.37525000	0.69063900
C	6.25314600	-3.95883800	1.98066700
H	5.75385900	-2.21368400	-0.66035400
H	7.14414200	-3.80438500	0.01233400
H	6.91297400	-4.75386800	2.33222700
H	6.64331200	-2.98802500	2.30244000
H	5.26465300	-4.11422200	2.41570100
N	5.45427100	-5.43982000	-1.37356000
C	5.07956800	-6.67959000	-2.01692700
C	3.64168000	-6.67161600	-2.54661600
O	2.66456300	-6.56917000	-1.52576600
H	5.39932000	-4.57760600	-1.92862500
H	5.17675400	-7.46343100	-1.26123600
H	3.53077600	-5.86049000	-3.28092500
H	3.46145000	-7.62102400	-3.06891200
H	2.66630700	-5.63625600	-1.25308200
C	0.09163600	1.11641000	-5.83257500
C	-0.03488900	0.56633900	-4.41286100
S	1.53873600	0.05900600	-3.60782600
H	-0.92450000	1.38599600	-6.17334400
H	-0.52304900	1.33639600	-3.79667600
H	-0.70203000	-0.30305400	-4.44495100
H	0.20079700	0.50527400	-1.77760900
C	8.76987600	-2.52614900	-4.32118700
C	8.01522200	-3.42891800	-3.33823800
O	8.08418300	-2.93924400	-2.00479000
H	8.92025400	-3.03556600	-5.28211000
H	6.98292300	-3.58628800	-3.66109400
H	8.50107400	-4.41373700	-3.31486000
H	7.53931000	-2.12767300	-1.91745300
C	7.89596900	1.20027800	-4.39816700
C	7.48384700	1.89411900	-3.08687500
C	6.16736800	1.37424300	-2.47773300
C	6.10530000	-0.15146600	-2.37846200
O	6.55016000	-0.69695700	-1.31927100
O	5.62837800	-0.77523200	-3.37126100
H	7.02690300	1.04379400	-5.04261800
H	8.29206900	1.78031900	-2.35237500
H	7.36535300	2.97258500	-3.25321300
H	6.05826300	1.79805100	-1.47685200
H	5.33613300	1.70265300	-3.10546400
C	-5.41322800	5.13193400	-4.16115500
C	-4.85394800	5.56465900	-2.79353800
C	-3.32762500	5.33718600	-2.69481700
C	-5.60873700	4.88505000	-1.64484500
C	-2.88015600	3.87495900	-2.56681300
H	-5.04173500	4.13545800	-4.42830600
H	-5.01915700	6.64793600	-2.69853900
H	-2.84623200	5.78377400	-3.57435300
H	-2.94699900	5.89374800	-1.82796300
H	-5.18648800	5.15695700	-0.67113000
H	-6.66664200	5.16923000	-1.64509300
H	-5.56778100	3.79604000	-1.72384500
H	-1.78871200	3.80907100	-2.59650500
H	-3.21791800	3.43456500	-1.62201100
H	-3.26403300	3.25008500	-3.37993800
C	-7.74060300	1.23169500	-6.43167500
C	-6.41077800	1.13286000	-5.66940000
C	-6.17784000	-0.21481600	-4.96571100
C	-6.23977600	-1.40291700	-5.93258000
C	-4.84415200	-0.20331800	-4.21454900
H	-7.71171700	0.66315100	-7.36696200
H	-5.57284300	1.33570400	-6.35082600
H	-6.37647000	1.92219200	-4.90660300
H	-6.98101500	-0.33834000	-4.22414600
H	-6.06152100	-2.34237900	-5.39965300
H	-7.21294500	-1.48028500	-6.42818800
H	-5.47010800	-1.31142500	-6.70869000
H	-4.70947000	-1.11970300	-3.63362600
H	-3.99806900	-0.12362300	-4.90701200
H	-4.79069900	0.64190200	-3.52210800

C	-10.31063400	0.83237100	-3.64954700	C	-0.51661300	-3.94790900	1.31834200
C	-10.32805000	1.59457700	-2.31311900	S	-2.29173800	-3.50052200	1.49148200
C	-9.09641300	1.23469900	-1.50144200	O	4.84962800	-3.40305500	-3.29965600
C	-9.18989000	0.36664900	-0.40644800	C	-1.53208800	-0.14097500	-0.64679200
C	-7.82288900	1.67094200	-1.89516700	C	-1.41552500	-1.05823700	0.35112700
C	-8.04973400	-0.07446900	0.26963000	C	-2.52452100	-1.86779200	0.83573500
C	-6.68042700	1.23189100	-1.22570100	O	-3.69261600	-1.43676900	0.90232400
C	-6.78895100	0.35078000	-0.14897600	C	-0.44355100	0.83056100	-0.91931700
H	-10.01158100	-0.20282200	-3.43912600	C	-2.68398300	-0.09938600	-1.61022500
H	-10.37204400	2.67620100	-2.49282000	H	-3.14448000	0.89390600	-1.62433300
H	-11.22536600	1.33193400	-1.74161200	H	-2.28780200	-0.27416000	-2.61698000
H	-10.16942200	0.01296600	-0.09336200	H	-3.45278900	-0.83748500	-1.39629700
H	-7.72374100	2.35071200	-2.73810200	H	-0.85713200	1.79433000	-1.23408600
H	-8.13259100	-0.76977700	1.10028400	H	-0.47164400	-1.15337200	0.87627600
H	-5.69916100	1.56716300	-1.54352300	H	0.20810400	0.97826200	-0.05740600
H	-5.89479200	-0.01149700	0.34389800	H	-0.53720700	-4.98645500	0.98229500
C	-2.37498500	-2.66068600	7.73255700	H	-0.04856500	-3.34650500	0.53907700
C	-2.38228900	-2.12569200	6.30945800	H	-0.37790700	-4.17615700	3.46885700
O	-2.75476500	-2.81738500	5.36404300	H	0.60151900	-2.84255900	2.82527700
H	-2.23487100	-3.74119700	7.70229100	H	2.20449800	-4.45459900	2.03798400
N	-1.99002800	-0.82520700	6.16865000	C	2.39733100	-6.98661700	2.54537600
C	-2.30490900	-0.04505000	4.95911700	H	2.37418600	-7.22545100	1.47482100
C	-3.83621700	-0.10058000	4.75670400	H	3.36556900	-6.51156000	2.72608100
O	-4.57521000	0.38019700	5.61952600	H	2.30805200	-7.90951100	3.11898300
C	-1.44112700	-0.43681100	3.76642600	H	-0.66299500	-4.76129000	-6.34065200
H	-1.90541500	-0.29120300	7.02060300	H	1.08209600	-4.90661500	-5.85968500
H	-2.10489200	0.99922800	5.20263500	H	5.75099200	-6.92928000	-2.84492100
H	-1.70370500	0.18643300	2.90891100	H	8.22429900	-1.59892900	-4.52327900
H	-0.38498300	-0.27401800	3.99309200	H	9.74847700	-2.34708400	-3.86286300
H	-1.57176500	-1.48939500	3.51389600	H	8.32525000	0.22222700	-4.15136600
N	-4.28740400	-0.71365500	3.64309500	H	8.60821700	1.83262700	-4.93923800
C	-5.68423900	-0.98997900	3.39049300	H	0.82202800	2.46948200	7.39469300
C	-5.98500000	-2.47012200	3.12079700	H	2.59412900	2.48889800	7.52758100
O	-7.00874400	-2.78662800	2.50935800	H	6.87473100	6.59035600	-0.25671200
H	-3.63778500	-1.05472400	2.95146900	H	5.06010000	6.71251600	-0.18085900
H	-6.24365800	-0.66203400	4.27074500	H	3.41987300	9.26695100	-1.82342400
H	-6.05195400	-0.42837900	2.52903300	H	4.16032500	7.68244000	-1.48520200
N	-5.10611600	-3.36108700	3.62716100	H	3.33245800	8.68538800	2.95217600
C	-5.27956700	-4.79021000	3.42287300	H	4.38201900	7.98613400	4.25203100
H	-4.28050500	-3.03934800	4.12282800	H	-2.05090000	8.29480600	3.65845500
H	-5.27652300	-5.02860000	2.35423500	H	-2.39067500	7.30919600	2.21140200
C	-8.87115300	-5.12682900	-0.10287900	H	-5.09753600	5.81596000	-4.95630000
C	-7.46578700	-4.79409400	-0.62331100	H	-6.50434200	5.04565300	-4.13517400
C	-7.21457600	-3.30448300	-0.91379000	H	-8.53834800	0.80243700	-5.81664800
C	-5.73578900	-3.05909400	-1.23237900	H	-7.95513600	2.27537700	-6.68398100
C	-8.09566900	-2.76654400	-2.04880600	H	-11.26687200	0.74029200	-4.17539100
H	-9.64111300	-4.82883700	-0.82425500	H	-9.52713900	1.22108000	-4.30803800
H	-6.73161700	-5.12309700	0.12272400	H	-9.06519400	-4.58137900	0.82657800
H	-7.26173800	-5.37613200	-1.53465400	H	-8.97333900	-6.20786400	0.03816000
H	-7.44917400	-2.75297800	0.00317300	H	-4.45312400	-5.32571500	3.90201900
H	-5.55669400	-1.99871400	-1.43369700	H	-6.23723000	-5.12213800	3.83768600
H	-5.09334100	-3.34683000	-0.39558300	H	-3.35390500	-2.44473200	8.17322600
H	-5.42244900	-3.62657500	-2.11837900	H	-1.59302500	-2.20153500	8.34644800
H	-7.88678100	-1.70751900	-2.22717900	H	0.42874600	0.36195800	-6.55139700
H	-7.90280300	-3.31159900	-2.98247900	H	0.66159300	2.04917100	-5.90062600
H	-9.16341800	-2.85479600	-1.82235900	H	3.93003100	-3.39509000	-2.99494900
C	1.22736500	-6.08120800	2.87926800	H	5.13530600	-2.45729900	-3.32371200
O	0.17117500	-6.51666200	3.33957100	O	4.26991400	1.09464300	-5.07897700
N	1.41460000	-4.74996400	2.61358900	H	4.75400700	0.38540200	-4.60995200
C	0.26500100	-3.86641600	2.64282300	H	3.34663100	0.88919600	-4.82565900

O	-3.18371600	2.65919400	3.61712300
H	-3.41653200	2.19928900	2.78306400
H	-3.90048700	2.43183100	4.22423600
O	-3.58181900	1.41008100	1.13890300
H	-3.68857500	0.43947700	1.05176600
